# Acquisition and evolution of the neurotoxin domoic acid biosynthesis gene cluster in *Pseudo-nitzschia* species

**DOI:** 10.1038/s42003-024-07068-7

**Published:** 2024-10-23

**Authors:** Ziyan He, Qing Xu, Yang Chen, Shuya Liu, Huiyin Song, Hui Wang, Chui Pin Leaw, Nansheng Chen

**Affiliations:** 1 CAS Key Laboratory of Marine Ecology and Environmental Sciences, Institute of Oceanology, Chinese Academy of Sciences, Qingdao, 266071 China; 2 Laboratory of Marine Ecology and Environmental Science, Qingdao National Laboratory for Marine Science and Technology, Qingdao, 266200 China; 3 College of Marine Science, University of Chinese Academy of Sciences, 10039 Beijing, China; 4 Center for Ocean Mega-Science, Chinese Academy of Sciences, Qingdao, 266071 China; 5 Hubei Key Laboratory of Tumor Microenvironment and Immunotherapy, College of Basic Medical Sciences, China Three Gorges University, Yichang, 443002 China; 6 Bachok Marine Research Station, Institute of Ocean and Earth Sciences, University of Malaya, 16310 Bachok, Kelantan Malaysia

**Keywords:** Molecular evolution, Ecological genetics

## Abstract

Of the hitherto over 60 taxonomically identified species in the genus of *Pseudo-nitzschia*, 26 have been confirmed to be toxigenic. Nevertheless, the acquisition and evolution of the toxin biosynthesis (*dab*) genes by this extensive group of *Pseudo-nitzschia* species remains unclear. Through constructing chromosome-level genomes of three *Pseudo-nitzschia* species and draft genomes of ten additional *Pseudo-nitzschia* species, putative genomic integration sites for the *dab* genes in *Pseudo-nitzschia* species were explored. A putative breakpoint was observed in syntenic regions in the *dab* gene cluster-lacking *Pseudo-nitzschia* species, suggesting potential independent losses of *dab* genes. The breakpoints between this pair of conserved genes were also identified in some *dab* genes-possessing *Pseudo-nitzschia* species, suggesting that the *dab* gene clusters transposed to other loci after the initial integration. A “single acquisition, multiple independent losses (SAMIL)” model is proposed to explain the acquisition and evolution of the *dab* gene cluster in *Pseudo-nitzschia* species.

## Abstract (web summary)

A “single acquisition, multiple independent losses (SAMIL)” model is proposed to explain the acquisition and evolution of the domoic acid biosynthesis gene cluster in Pseudo-nitzschia species.

## Introduction

Species of the diatom genus *Pseudo-nitzschia* have attracted worldwide attention since the first reported *Pseudo-nitzschia* poisoning incident in 1987 at Prince Edward Island, Canada, which resulted in at least three deaths and 107 cases of illness, and the identification of the neurotoxic glutamate receptor agonist domoic acid (DA) as the toxin synthesized by *P. multiseries*^1,2^. DA synthesized by *Pseudo-nitzschia* species has been found to be transferred in the marine food web during bloom events, which may cause illness or death for marine organisms and humans, leading to what is called amnesic shellfish poisoning (ASP)^2^. An unprecedented bloom of another toxigenic *P. australis* in the spring of 2015 resulted in the largest recorded outbreak of DA along the North American west coast, ranging from the Aleutian Islands of Alaska, USA, to the Baja peninsula, Mexico, and resulted in prolonged closures of razor clam, rock crab, and Dungeness crab fisheries^3^. Similar neurotoxic symptoms have also been observed in animals, including birds and marine mammals^4^. In addition, numerous harmful algal bloom (HAB) events caused by *Pseudo-nitzschia* species have continued to erupt globally, exerting severe impacts on fisheries and the aquaculture industry^5–7^.

*Pseudo-nitzschia* blooms have motivated intensive research on the identification and geographical distribution of new *Pseudo-nitzschia* species, and on the elucidation of mechanisms of DA biosynthesis in *Pseudo-nitzschia* species, which resulted in the identification of key genes of the DA biosynthetic pathway in *P. multiseries* through comparative gene expression analysis^8^. This study successfully identified a gene cluster, *dab*, with four protein-coding genes: *dabA* (terpene cyclase), *dabB* (hypothetical protein), *dabC* (ɑ-dependent dioxygenase), and *dabD* (CYP450), which encode key enzymes catalyzing reactions extending from the substrate glutamate and geranyl pyrophosphate (GPP) to isodomoic acid A in the genome of *P. multiseries*^8^. Following this success, *dab* genes were readily identified in *P. multistriata* through genomic analysis and the search for homologous genes^8^, and in *P. australis* and *P. seriata* through transcriptomic analyses^8–10^. The characterization of the *dab* gene clusters has also facilitated research on the impact of ocean warming and acidification on DA biosynthesis^11^.

*Pseudo-nitzschia* is a genus with over 60 cosmopolitan species, up to 50% of which are capable of synthesizing DA^8,12,13^. This may explain the wide distribution of DA from the Pacific subarctic (58°N) to the Southern Ocean (66°S)^14^. Notably, *Pseudo-nitzschia* species that are capable of producing DA are not necessarily phylogenetically closer. Furthermore, of the 26 DA-producing *Pseudo-nitzschia* species, 24 were found to produce DA in some studies, but not in others^2^, suggesting that DA biosynthesis is not only species-specific but also strain-specific. Thus, whether the *dab* gene cluster was acquired independently in different *Pseudo-nitzschia* species, or it was acquired in the common ancestor of all *Pseudo-nitzschia* species remains undetermined. Ascertaining the acquisition and evolution of the *dab* gene cluster in *Pseudo-nitzschia* species will help explain why certain *Pseudo-nitzschia* species are toxic, while others are not, and why some species are reported to exhibit both toxic and non-toxic traits^2,12,13^.

In this project, we constructed the genomes of 13 *Pseudo-nitzschia* species, including high quality chromosome-level genomes of three species (*P. delicatissima*, *P. multiseries*, and *P. pungens*) and draft-quality genomes of ten species (*P. americana*, *P. brasiliana*, *P. cuspidata*, *P. galaxiae*, *P. hainanensis*, *P. micropora*, *P. multistriata*, *P. sabit*, *Pseudo-nitzschia* sp. (CNS00097), and *Pseudo-nitzschia* sp. (CNS01031)), and identified the *dab* gene clusters. Of the three *Pseudo-nitzschia* species selected for constructing chromosome-level genomes, *P. multiseries* has been proven to possess the *dab* gene cluster and is toxic^8^, while *P. pungens* was shown to be both toxic and non-toxic, depending on the strains^2^, *P. delicatissima*, on the other hand, was found to be toxic, weakly toxic, or non-toxic^15^. Comparative genomic analysis of syntenic regions harboring *dab* gene clusters, coupled with phylogenetic analysis of *dab* genes and structural modeling of the proteins encoded by these *dab* genes, enabled us to identify the putative integration sites of the *dab* gene cluster in the common ancestor of *Pseudo-nitzschia* species. Our findings suggested a “single acquisition, multiple independent losses (SAMIL)” model for the evolution of the *dab* gene cluster in *Pseudo-nitzschia* species.

## Results

### Construction and comparative analysis of chromosome-level genomes of three *Pseudo-nitzschia species*

To obtain high-quality reference genomes for the three *Pseudo-nitzschia* species, we generated 15.06 Gb (~442×), 20.40 Gb (~261×), and 31.87 Gb (~119×) of PacBio data (Supplementary Table 1). These data were assembled to produce the initial versions of the whole genome assemblies, which were 34.06 Mb, 67.09 Mb, and 252.10 Mb in sizes for *P. delicatissima*, *P. pungens*, and *P. multiseries*, respectively. These assembly results were generally consistent with the estimated sizes obtained from genome survey analysis (Supplementary Fig. 1**;** Supplementary Table 2).

**Fig. 1:**
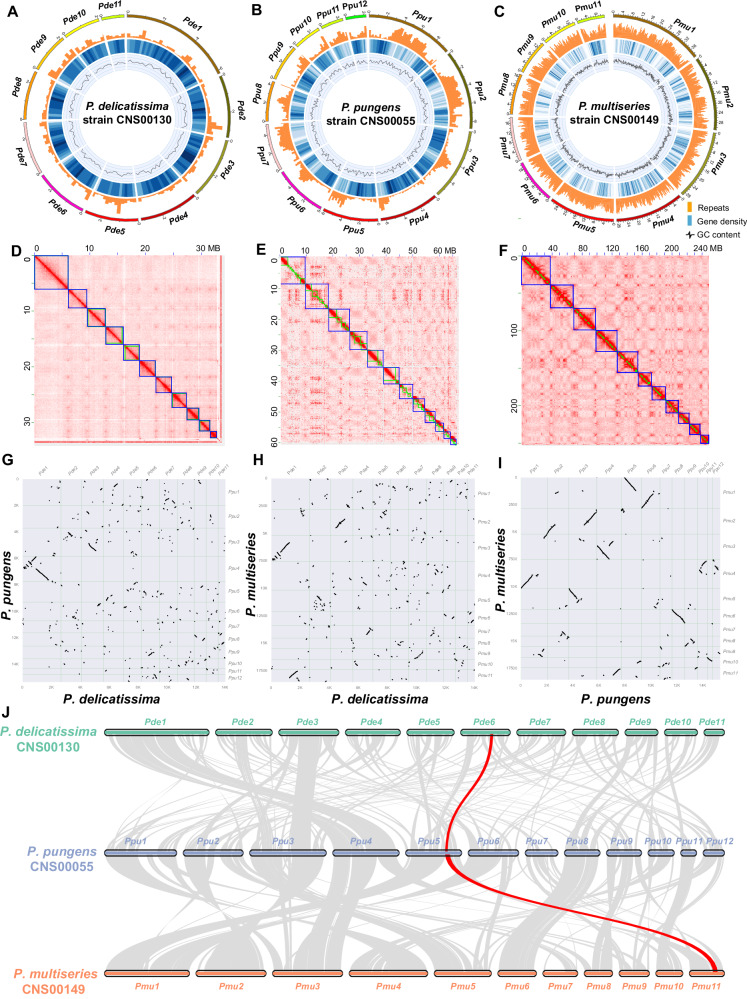
Construction and comparative analysis of chromosome-level genome assemblies of three *Pseudo-nitzschia* species. **A** Genome of *P. delicatissima* (CNS00130) consists of 11 chromosomes (outmost circle). **B** Genome of *P. pungens* (CNS00055) consists of 12 chromosomes. **C** Genome of *P. multiseries* (CNS00140) consists of 11 chromosomes. Hi-C intra-chromosomal contact maps of the genome assemblies in *P. delicatissima* (**D**), *P. pungens* (**E**), and *P. multiseries* (**F**). **G** Comparison between *P. delicatissima* and *P. pungens*. **H** Comparison between *P. delicatissima* and *P.multiseries*. **I** Comparison between *P. pungens* and *P. multiseries*. **J** Syntenic relationships among *P. delicatissima*, *P. multiseries*, and *P. pungens*. The sites linked by the red line indicate the putative original *dab* gene cluster integration site in each genome.

We further carried out Hi-C (High-through chromosome conformation capture) analysis (Supplementary Table 3) to construct chromosome-level assemblies, which were 34.06 Mb, 67.11 Mb, and 252.35 Mb for *P. delicatissima*, *P. pungens*, and *P. multiseries*, respectively (Fig. 1; Table 1). The genome assemblies of *P. delicatissima*, *P. pungens* and *P. multiseries* contained 11, 12, and 11 chromosomes (Supplementary Table 4) and were highly contiguous, with 96.48%, 99.14%, and 94.90% of genome contigs anchored to chromosomes, respectively. The assemblies also had high contig N50 values of 2.90 Mb, 1.66 Mb, and 0.80 Mb for *P. delicatissima*, *P. pungens*, and *P. multiseries*, respectively (Table 1). BUSCO assessment indicated that the genome assemblies were 73.93%, 80.53%, and 81.52% complete for *P. delicatissima*, *P. pungens*, and *P. multiseries*, respectively (Supplementary Table 5).

**Table 1:** Statistics of *Pseudo-nitzschia* genome assembly and annotation

	*P. delicatissima* S06	*P. pungens* S07	*P. multiseries* S08	*P. multistriata*^83^	*P. multiseries*^83^
Sequencing					
Illumina sequencing					
Raw data (Gb)	27.85	44.24	27.98	–	–
Sequencing depth (X)	818	497	105	–	–
PacBio sequencing					
Raw data (Gp)	15.06	20.40	31.87	–	–
Sequencing depth (X)	442	261	119	–	–
Average reads length (bp)	13,343	11,652	13,659	–	–
Genome survey					
Estimated genome size (Mb)	35.37	78.03	266.95	–	–
Heterozygous Ratio (%)	1.37	0.82	0.87	0.18	–
Repeat (%)	11.00	42.10	71.00	–	–
Assembly features					
Genome size (Mb)	34.06	67.11	252.35	59	219
Scaffolds number	60	62	110	1099	–
Contig number	70	161	580	3430	–
Contig N50 (Mb)	2.90	1.66	0.80	138	147
BUSCO assembly (%)	73.93	80.53	81.52	–	–
Genome annotation					
Number of protein-coding genes	14,375	15,472	18,649	12,008	19,703
Average gene length (bp)	1472	1934	1824	2205	1522
Total size of TEs (Mb)	0.59	23.43	173.29	14.75	159.87
TEs in genome (%)	1.73	34.91	68.67	25.00	73.00
Total size of Satellite (Mb)	0.01	0.00	0.67	–	–
Satellite in genome (%)	0.03	0.00	0.26	–	–
Total size of Simple repeat (Mb)	0.00	0.05	3.00	–	–
Simple repeat in genome (%)	0.00	0.07	1.19	–	–
BUSCO annotation (%)	70.96	82.51	83.83	–	–

We explored why the genome sizes of three *Pseudo-nitzschia* species were so different, ranging from 34.06 Mb (*P. delicatissima*) to 252.10 Mb (*P. multiseries*). Annotation of the assembled genomes revealed that *P. multiseries* harbors a greater proportion of repetitive elements (68.67%) compared to *P. delicatissima* (1.73%) and *P. pungens* (34.91%). Among all types of repetitive elements, long terminal repeats (LTRs) showed the highest proportions, accounting for 0.84%, 22.94%, and 58.45% of the genomes of *P. delicatissima*, *P. pungens*, and *P. multiseries*, respectively (Table 2). This suggested that differential levels of LTRs were primarily responsible for the size differences among these three *Pseudo-nitzschia* species. Despite the differences in genome sizes, the numbers of protein-coding genes (PCGs) in these three species were similar, being 14,375, 15,472, and 18,649 in *P. delicatissima*, *P. pungens*, and *P. multiseries*, respectively (Table 1; Supplementary Table 6). Notably, the number of PCGs predicted in *P. multiseries* in this study (18,649) was similar to that annotated in a different *P. multiseries* strain (19,703). Most PCGs of the *P. delicatissima*, *P. pungens*, and *P. multiseries* genomes (93.83%, 91.44%, and 88.49%, respectively) were functionally annotated (Supplementary Table 7).

**Table 2:** Repetitive contents identified in the three *Pseudo-nitzschia* genomes

	*P. delicatissima*		*P. pungens*		*P. multiseries*	
Type	Length (bp)	% in genome	Length (bp)	% in genome	Length (bp)	% in genome
DNA TE	192,168	0.56	1,149,438	1.71	11,246,223	4.46
LINE	118,203	0.35	7,024,138	10.47	22,525,999	8.93
SINE	44,132	0.13	97,712	0.15	11,928	0.00
LTR	287,530	0.84	15,393,159	22.94	147,486,486	58.45
Other	585	0.00	53	0.00	434	0.00
Unknown	0	0.00	3,163,547	4.71	0	0.00
Total	589,444	1.73	23,428,954	34.91	173,294,434	68.67

Although these three species belong to the same genus, their genomes showed extensive differences with varying numbers of chromosomes, indicating many intra-chromosomal fusion or divisions events occurred during speciation. While 11 chromosomes were constructed for *P. delicatissima* and *P. multiseries*, 12 chromosomes were constructed for *P. pungens* (Fig. 1). Pairwise comparative analyses revealed only small-scale syntenic blocks between the genomes of *P. delicatissima* and *P. pungens* (Fig. 1G) and between the genomes of *P. delicatissima* and *P. multiseries* (Fig. 1H), suggesting complex evolutionary relationships with numerous chromosomal exchanges. Nevertheless, relatively large syntenic block sizes were found between genomes of *P. pungens* and *P. multiseries* (Fig. 1I), consistent with their close positions in the phylogenetic tree (Supplementary Fig. 2). In particular, chromosome *Pmu6* of *P. multiseries* and chromosome *Ppu8* of *P. pungens* showed nearly perfect collinearity (Fig. 1I-J). A total of 10,755 collinear gene pairs were found between *P. pungens* and *P. multiseries*, 9076 collinear gene pairs between *P. delicatissima* and *P. pungens*, and 9272 collinear gene pairs between *P. delicatissima* and *P. multiseries* (Fig. 1G–I).

To explore gene family dynamic changes in evolution, PCGs of *P. delicatissima*, *P. pungens*, and *P. multiseries* were compared with those of nine other phytoplankton species, including *Aureococcus anophagefferens*, *Thalassiosira oceanica*, *Thalassiosira pseudonana*, *Skeletonema marinoi*, *Seminavis robusta*, *Phaeodactylum tricornutum*, *Fragilariopsis cylindrus*, *P. multistriata*, and *P. multiseries* (Supplementary Fig. 3A). Phylogenetic analysis using 381 single-copy orthologous genes from these 12 species showed that *Pseudo-nitzschia* species were tightly clustered with *F. cylindrus*, as expected. The divergence time of *Pseudo-nitzschia* species from their nearest node was estimated to be approximately 53.2 million years ago (MYA) (Supplementary Fig. 3A). Comparative analysis of the genes of *P. delicatissima*, *P. pungens*, *P. multiseries*, and *P. multistriata* revealed 7136 shared gene families (Supplementary Fig. 3B).

Comparative analysis of Pfam domains contained in the PCGs of the 12 species revealed that many gene families encoded by the *Pseudo-nitzschia* genomes were different from those in other species (Supplementary Fig. 3C). Notably, the family of genes encoding gametolysin peptidase M11 was substantially expanded in *Pseudo-nitzschia* genomes, with 17, 50, and 33 members in *P. delicatissima*, *P. pungens*, and *P. multiseries*, respectively (Supplementary Fig. 3C, Supplementary Fig. 3D). These gametolysin peptidase M11 domain-containing proteins, which have their origin in the ancestor of green plants and chromists, have been demonstrated to degrade cell wall in *Chlamydomonas*^16^.

### Annotation of *dab* gene clusters and phylogenetic analysis of *dab* genes

**Fig. 2:**
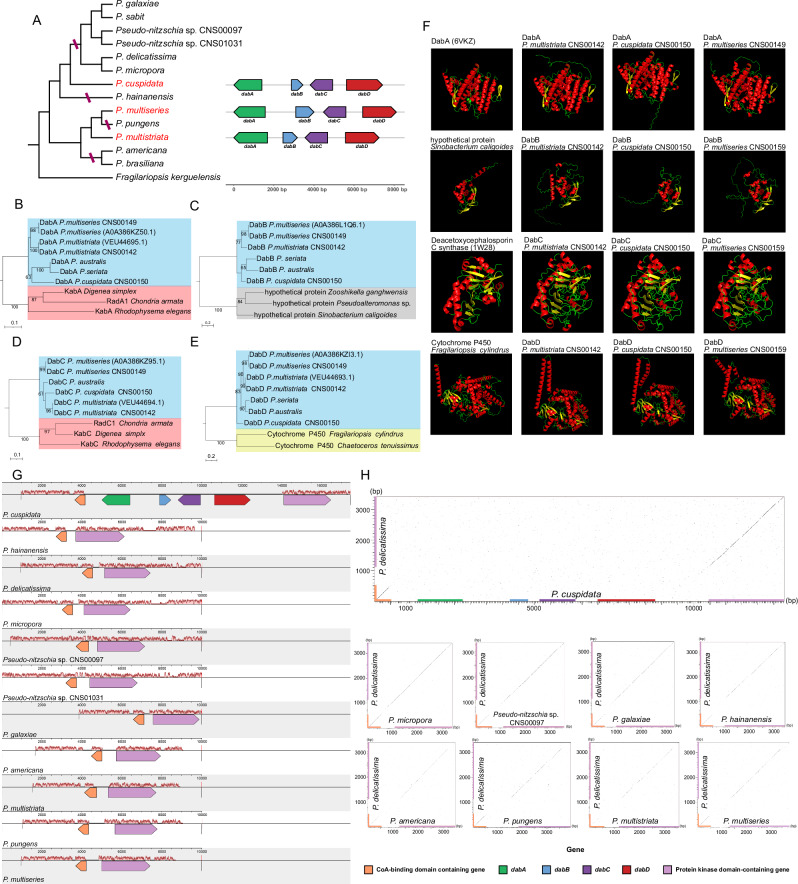
Phylogenetic and syntenic analysis of DA biosynthesis genes. **A** Phylogenetic analysis of *Pseudo-nitzschia* species based on cpDNAs illustrating the positioning of the *Pseudo-nitzschia* species (shown in red) that possess the *dab* gene clusters (which are displayed to the right of the species names). **B** Phylogenetic tree displaying *Pseudo-nitzschia* DabA proteins and KabA proteins. **C** Phylogenetic tree displaying *Pseudo-nitzschia* DabB proteins and hypothetical proteins that showed the highest similarities. **D** Phylogenetic tree displaying *Pseudo-nitzschia* DabC proteins and proteins that showed the highest similarities. **E** Phylogenetic tree displaying *Pseudo-nitzschia* DabD proteins and Cytochrome CYP450 proteins that showed the highest similarities. **F** AlphaFold2-predicted 3D structures of DabA, DabB, DabC and DabD proteins identified in *Pseudo-nitzschia* species and related species. **G**. Comparative synteny analysis between the putative “original” integration site of the *dab* gene cluster in *Pseudo-nitzschia* species. Only the *P. cuspidata* genome contains a *dab* gene cluster between the gene pairs. **H**. Dot-plot illustration of the breakpoints of the “original” integration site of the *dab* gene cluster.

From the three chromosome-level *Pseudo-nitzschia* genomes constructed in this study, the *dab* gene cluster was identified only in *P. multiseries* (Fig. 2A**;** Supplementary Table 8), which is the first *Pseudo-nitzschia* species known to possess the *dab* gene cluster^8^. The *dab* gene cluster in *P. multiseries* consisted of four genes: *dabA*, *dabB*, *dabC*, and *dabD*, as reported previously^8^. However, the *dab* gene cluster was not identified in either *P. delicatissima* or *P. pungens* (Fig. 2A**;** Supplementary Table 8), despite previous studies showing the presence of both toxic and non-toxic strains in these two *Pseudo-nitzschia* species^2,8,15^. Searches for individual genes with similarity to those in the *dab* gene cluster did not yield meaningful hits in the assemblies of *P. delicatissima* and *P. pungens*. The *dab* gene cluster was identified in the draft genomes of *P. multistriata* strains in this study; this species has previously been shown to possess the *dab* gene cluster^8^. The cluster was also detected in the assembly of *P. cuspidata* (Fig. 2A**;** Supplementary Table 8), a species previously reported as DA-producing^2,17^. No *dab* gene clusters were found in the draft genomes of other *Pseudo-nitzschia* species investigated in this study, despite some being reported as DA producers^18^. Interestingly, the *Pseudo-nitzschia* species found to possess the *dab* gene cluster in this study (*P. multiseries*, *P. multistriata*, and *P. cuspidata*), as well as two other species (*P. australis* and *P. seriata*) identified through transcriptomic analysis, were distributed throughout the phylogenetic tree of *Pseudo-nitzschia* species (Supplementary Fig. 2), suggesting that the acquisition and evolution of the *dab* gene cluster are intricate.

Chemical analysis performed on 13 strains of nine *Pseudo-nitzschia* species (*P. multiseries*, *P. multistriata*, *P. delicatissima*, *P. americana*, *P. micropora*, *P. pungens*, *P. galaxiae*, *P. brasiliana*, and *Pseudo-nitzschia* sp. (CNS00097)) revealed that DA was detected only in the strain of *P. multiseries*. No DA was detected in the *P. multistriata* strains, even though five *P. multistriata* strains were analyzed (CNS00142, CNS00781, CNS00965, CNS01237, CNS01424). DA analysis was not performed for strains of *P. cuspidata*, *P. hainanensis*, *P. sabit*, and *Pseudo-nitzschia* sp. CNS01031 due to their unavailability, as these strains unfortunately died off during the study period (Supplementary Table 8).

### Acquisition and evolution of *dab* gene cluster

To determine how *dab* gene clusters were acquired by *Pseudo-nitzschia* species, we hypothesized that the *dab* gene cluster was acquired by a common ancestor of *Pseudo-nitzschia* species and was subsequently lost independently in different species. Based on the phylogenetic positions of *Pseudo-nitzschia* species that possess the *dab* gene cluster (Supplementary Fig. 2), we predicted that the phylogenetic relationships of the *dab* genes would align with the phylogenetic relationships of these species. To test this hypothesis, we conducted phylogenetic analyses of chloroplast genomes (cpDNAs) to infer the phylogenetic relationships (Fig. 2A), as rDNA molecular markers were relatively short and some species exhibited cryptic lineages with ambiguous relationships^19^. Separate phylogenetic analysis of the *dab* genes was also performed. As expected, the phylogenetic inferences for DabA (Fig. 2B), DabB (Fig. 2C), DabC (Fig. 2D), and DabD (Fig. 2E) were highly coherent with the phylogenetic relationships of the *Pseudo-nitzschia* species harboring the *dab* gene cluster (Fig. 2A**;** Supplementary Fig. 3). These results suggested that the *dab* gene cluster in these three *Pseudo-nitzschia* species most likely originated from a common ancestor that gained the dab genes via a horizontal gene transfer (HGT) event. In fact, the proteins encoded by *dabB* exhibited high similarity to proteins found in prokaryotic organisms (Fig. 2C), supporting the hypothesis of HGT. Furthermore, modeling of the 3D structures of the proteins encoded by *dabB* and *dabC* revealed high structural similarity to proteins found in prokaryotic organisms (Fig. 2F), further supporting the gene recruitment from prokaryotic donors via HGT^20^. Proteins encoded by *dabA* identified in *P. multistriata* (CNS00142), *P. multiseries* (CNS00149), and *P. cuspidata* (CNS00150) in this study showed high structural similarity to DabA of *P. multiseries* reported previously^20^, and proteins encoded by *dabD* identified in this study showed high structural similarity to Cytochrome P450 of *Fragilariopsis cylindrus* (Fig. 2F), indicating a high conservation of this gene in diatoms.

The hypothesis further predicted that the *dab* gene clusters would be present in homologous integration sites (i.e., sharing breakpoints) in the genomes of all *Pseudo-nitzschia* species that possess the *dab* gene clusters, and that deletion events (i.e., *dab* gene cluster losses) would be evident in the genomes of species that do not possess the *dab* gene clusters. Our comparative analysis of the genomes of *P. multiseries*, *P. multistriata*, and *P. cuspidata*, however, indicated that the genomic segments harboring the *dab* gene clusters were not homologous (Supplementary Fig. 4). We therefore anticipated that the *dab* gene clusters might have moved away from their “original” integration sites due to genome rearrangements, as all *Pseudo-nitzschia* species have undergone extensive shuffling and transposition (Fig. 1J).

We then searched for the putative “original” integration sites of the *dab* gene cluster in *Pseudo-nitzschia* genomes, including those with and without the *dab* gene clusters. To trace the loss of the *dab* gene cluster, we aligned the genome scaffold sequences containing the *dab* gene from *P. multiseries*, *P. multistriata*, and *P. cuspidata* with the genomic sequences of other *Pseudo-nitzschia* using BLAST. Our results showed that the *dab* gene cluster in *P. cuspidata* was flanked by two genes, *Pde10317* (a CoA-binding protein) and *Pde10588* (a protein kinase) (Fig. 2G, H). The upstream gene, *Pde10317*, encodes a CoA-binding protein. BLAST analysis indicated that homologous genes of *Pde10317* can be found in all *Pseudo-nitzschia* species and many closely related species, including genera such as *Nitzschia*, *Fragilariopsis*, *Phaeodactylum*, and *Cylindrotheca*. Conversely, *Pde10588* encodes a protein kinase, with homologs present in essentially all eukaryotes.

Through comparison with the SWISS-PROT database, it was found that the CoA-binding domain-containing gene exhibits homology with the *Escherichia coli yccU* (Percent Identity = 57.1%, Alignment Length = 137)^21,22^. Interestingly, this gene pair was successfully identified in all 13 *Pseudo-nitzschia* genomes analyzed in this project as well as in many other genomes (Fig. 2), suggesting that the putative original integration sites of the *dab* gene clusters remained intact in these genomes. Pairwise comparison of the genomic regions harboring the original integration sites revealed that the genomic regions between the gene pair were rather different (Supplementary Fig. 5), suggesting that the losses of the *dab* gene cluster were species-specific and varied among the genomes.

## Discussion

HABs not only impact marine ecosystems by generating overwhelming biomass^23,24^, but also pose risk to humans and other life forms by producing deadly toxins^25^. The discovery of key genes in the DA biosynthetic pathway in *P. multiseries* in 2018^8^ represented a milestone in understanding the mechanisms underlying the impact of ocean warming and acidification on DA production, and also paved the way for identifying DA biosynthesis genes in other algal species^26,27^. For example, kainic acid biosynthesis (*kab*) gene clusters were successfully identified in the genomes of two known kainic acid producers, *Digenea simplex* and *Palmaria palmata*, through homology-based searches. This was based on the observation that kainic acid and domoic acid share structural similarities (both compounds are commonly referred to as kainoids)^28^. Similarly, the *dab* biosynthesis gene cluster was found in the red alga *Chondria armata*, the seaweed from which DA was first characterized. Phylogenetic analysis suggested that the core DA biosynthesis genes were acquired through horizontal gene transfer^29^.

To facilitate the identification of *dab* genes across various *Pseudo-nitzschia* species, we assembled chromosome-level genomes for three species (*P. multiseries*, *P. delicatissima*, and *P*. *pungens*) and draft genomes for ten additional species (including two undescribed species), encompassing both toxigenic and non-toxic *Pseudo-nitzschia* species^2^. Insights gained from these genomes through comparative genomics offer valuable understanding of the evolutionary processes underlying the divergence in toxin production capabilities. Additionally, this information sheds light on how genomic context affects gene expression strength and its potential link to disruptions in genes or regulatory elements. Furthermore, these findings provide a point of reference for future transcriptomic studies and enhance our ability to investigate the genetic basis of toxin production in *Pseudo*-*nitzschia* species.

### The “single acquisition, multiple independent losses (SAMIL)” model of *dab* gene cluster evolution in *Pseudo-nitzschia* species

Through mining of these genomes, we identified the DA biosynthesis *dab* gene cluster in only three *Pseudo-nitzschia* species. We proposed a putative original integration site and breakpoints for the *dab* gene cluster in species that lack DA metabolism. Surprisingly, the *dab* gene cluster-containing *Pseudo-nitzschia* species was low, accounting for only 20% (3 out of 13) of the species analyzed in this study. This is unexpected given that up to 50% of *Pseudo*-*nitzschia* species are known to synthesize DA^8,12,13^. Nevertheless, numerous studies have reported both toxic and non-toxic strains w *Pseudo-nitzschia* species^2^. It is possible that some species might have been erroneously annotated in earlier studies due to their morphological resemblance to toxigenic *Pseudo-nitzschia* species, particularly those identified solely through light microscopy^2^. The challenge of accurate species identification, especially in earlier morphology-based studies, has been recognized as a significant issue in accurately identifying *Pseudo-nitzschia* species^30,31^.

**Fig. 3:**
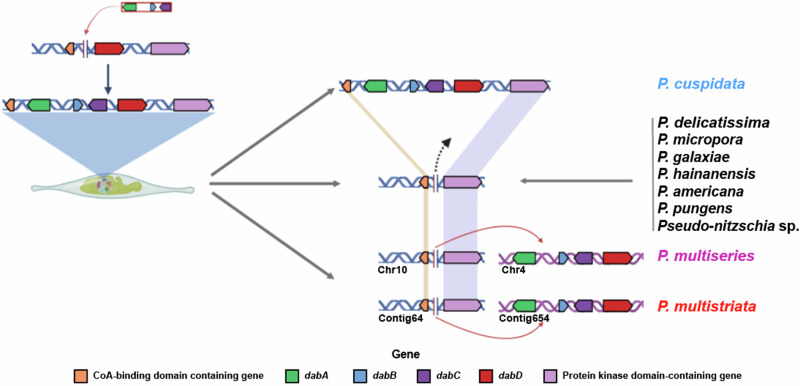
The SAMIL (Single acquisition, multiple independent losses) model of the *dab* gene cluster evolution in *Pseudo-nitzschia* lineages.

Based on the results of this study, we propose a “single acquisition, multiple independent losses (SAMIL)” model to explain the evolution of the *dab* gene cluster in *Pseudo-nitzschia*. The model suggests that the common ancestor of all *Pseudo-nitzschia* species acquired the *dab* gene cluster either via HGT from prokaryotes or through endosymbiosis with red algae (Fig. 3). Notably, since the sequence of CYP450 encoded by *dabD* is closest to other diatom P450s (Fig. 2E), we propose that the ancestral gene cluster gained via HGT contained only *dabA*, *dabB*, and *dabC*, with *dabD* being laterally acquired or “hijacked” for DA biosynthesis^29^. After this acquisition, the *dab* gene cluster underwent independent evolutionary changes in different *Pseudo-nitzschia* lineages. Under neutral or negative selection, the *dab* gene cluster may have been lost from the genomes of some non-DA-producing *Pseudo-nitzschia* lineages (e.g., *P*. *sabit*, *P*. *americana*). In addition, intraspecific variation in DA metabolism within “toxic” lineages may result from recent evolutionary events (such as pseudogenization and gene flow), likely driven by adaptations to environmental stresses (geographical or physiological adaptations^10^). These may have led to the loss of the *dab* gene cluster in certain strains/populations, ultimately affecting their DA metabolism (*P. delicatissima*, *P*. *pungens*) study.

The location of the *dab* gene cluster integration in *P. cuspidata* may represent a putative alternative insertion site. Our comparative analysis of the genomic locations of *dab* gene cluster-containing *Pseudo-nitzschia* species and those lacking the *dab* gene cluster identified a potential “original” *dab* gene cluster insertion site in *P. cuspidata*. This original insertion site is flanked by two highly conserved genes: a CoA-binding protein and a protein kinase (Fig. 3). Notably, *dabD* is also flanked by these two genes. Our analysis further suggested that the *dab* gene cluster in *Pseudo-nitzschia* species might have undergone genome rearrangements (e.g., segmental or dispersal duplications), as evidenced by the different genomic locations of the *dab* gene clusters in *P*. *multiseries*, *P*. *multistriata*, and *P*. *cuspidata* (Fig. 3). Nevertheless, it cannot be ruled out that the original *dab* gene cluster integration site might be located at other genomic regions, such as the shared region between *P. multiseries* and *P. multistriata* (in blue, Supplementary Fig. 4). Additional data from more *dab* gene cluster-containing *Pseudo-nitzschia* species are needed to accurately pinpoint the bona fide original *dab* gene cluster integration site. Figure 3 was created with BioRender.com.

### The *dab* gene cluster and differential DA biosynthesis in *Pseudo-nitzschia* species

Research has shown that varying toxic characteristics can exist among different strains of the same *Pseudo-nitzschia* species, with some species comprised both toxic and non-toxic strains. This variability may be due to the inherent genetic diversity within *Pseudo-nitzschia* species^32^. Intraspecific variation in toxicity can be attributed to differences in the presence or absence of key biosynthetic genes within the *dab* gene clusters, or the presence or absence of entire *dab* gene clusters, as well as variations in regulatory elements controlling toxin production. In this study, we observed variability in the presence or absence of the entire *dab* gene cluster among different *Pseudo-nitzschia* species. However, no genomic differences were observed regarding the presence or absence of *dab* genes or the entire *dab* gene cluster among different strains of the same species. Of the 13 *Pseudo-nitzschia* species analyzed, seven (*P. brasiliana*, *P. cuspidata*, *P. delicatissima*, *P. galaxiae*, *P. multiseries*, *P. multistriata*, and *P. pungens*) were reported as toxigenic^2,18^. Despite this, the *dab* gene cluster was only identified in strains of three *Pseudo-nitzschia* species (*P. cuspidata*, *P. multiseries*, and *P. multistriata*). It is possible that strains of *P. brasiliana*, *P. delicatissima*, *P. galaxiae*, and *P. pungens* analyzed in this study lack the *dab* gene cluster, but it may exist in strains of these species from other geographical regions. Therefore, the presence of the *dab* gene cluster may be both species- and strain-specific.

Strains of *Pseudo-nitzschia* that carry the *dab* gene cluster may not necessarily produce DA. The toxicity of *Pseudo-nitzschia* species is influenced by a variety of ecological factors, both biotic and abiotic, which affect transcriptional and translational regulations, as suggested previously^11,33,34^. For instance, grazing by copepods and *Calanus* copepodite has been reported to induce toxin production in various *Pseudo-nitzschia* species^35^. Abiotic factors such as temperature, salinity, light exposure, pH, and concentrations of macronutrients and trace elements also significantly affect the toxin production capabilities of *Pseudo-nitzschia* species^36^. Even if all strains of a particular *Pseudo-nitzschia* species carry the *dab* gene cluster, different strains may still exhibit varying levels of DA production due to the differential regulation of the *dab* gene cluster at the transcriptional or translational levels. In fact, under similar culture conditions in this study, while strains of *P. multiseries* were found to produce DA, *P. multistriata* strains did not, despite possessing the *dab* gene cluster. This highlights the complex interplay between genetic diversity and environmental factors in shaping the toxicity of *Pseudo-nitzschia* species. Further research is needed to understand the molecular mechanisms governing toxin production at both the species and strain levels. Such insights are crucial for predicting and managing the potential impacts of harmful algal blooms on marine ecosystems and public health.

## Conclusion

Our genomic analyses have enabled the identification of the *dab* gene cluster in *P. cuspidata*. The *dab* gene cluster identified in this study and previous studies^8^ suggests a possible shared evolutionary origin in *Pseudo*-*nitzschia*. The complex evolutionary trajectory of the *dab* gene clusters in *Pseudo-nitzschia* can be succinctly summarized using the SAMIL model, which illustrates the dynamic gains and losses of DA biosynthetic capabilities throughout evolutionary history.

## Methods

### *Pseudo-nitzschia* strain isolation and identification

Strains of *Pseudo-nitzschia* analyzed in this study were isolated from various coastal regions in China, including Qinhuangdao, Hebei Province; Jiaozhou Bay, Shandong Province; Nan’ao Island and Qinzhou Bay, Guangdong Province (Supplementary Table 8). Cells were isolated using micropipette and subsequently cultured in L1 medium (1‰ volume fraction Na_2_SiO_3_⋅9H_2_O was added). Cultures were maintained at 19 °C, under cool white fluorescent illumination with an intensity of 30 μmol·m^−2^·s^−1^ and a 12:12 photoperiod. Strains were identified based on their morphological features and the molecular sequences of the nuclear-encoded ITS (ITS1-5.8S-ITS2) region of the ribosomal RNA gene. The identity of some strains were described previously, including CNS00043, CNS00055, CNS00089, CNS00110, CNS00141, CNS00153, CNS00154, CNS00155, and CNS00156^37^, CNS00133^38^, CNS00135^39^, and CNS00090, CNS00097, CNS00130, CNS00138, CNS00142, CNS00159 and CNS00150^40^. This study identified eleven *Pseudo-nitzschia* species, including *P. americana* (CNS00108, CNS00138 and CNS00151), *P. brasiliana* (CNS01029), *P. cuspidata* (CNS00150), *P. delicatissima* (CNS00130 and CNS00135), *P. galaxiae* (CNS01037 and CNS01103), *P. hainanensis* (CNS00090), *P. micropora* (CNS00133 and CNS01024), *P. multiseries* (CNS00149, CNS00159 and CNS00771), *P. multistriata* (CNS00107, CNS00142, CNS00781, CNS00965, CNS01237 and CNS01424), *P. pungens* (CNS00043, CNS00055, CNS00089, CNS00110, CNS00141, CNS00153, CNS00154, CNS00155, CNS00156, CNS00973, CNS01028, CNS01042 and CNS01238) and *P. sabit* (CNS00609), plus two previously undescribed species *Pseudo-nitzschia* sp. strains CNS00097 and CNS01031 (Supplementary Table 9).

### DNA extraction, sequencing, and draft genome assemblies

During the exponential growth phase, healthy algal cells were harvested for DNA extraction by centrifugation at 8000 rpm for 5 min. Total DNA was extracted using the DNAsecure Plant Kit (Tiangen Biotech, Beijing, China) following the manufacturers’ instructions. DNA concentration was quantified with the Qubit® DNA Assay Kit using a Qubit® 3.0 Flurometer (Invitrogen, USA). Genomic DNA from each sample (0.2 µg) was fragmented to approximately 350 bp by sonication (Covaris S220, Covaris, USA). The DNA fragments were then harvested, end-polished, A-tailed, and ligated with adapters for Illumina sequencing, followed by PCR amplification. PCR products were purified with the AMPure XP system (Beckman Coulter, Beverly, USA). Qualified libraries were sequenced on the NovaSeq 6000 PE150 platform (Illumina, San Diego, CA, USA) at Novogene (Beijing, China). Draft genomes were assembled using SPAdes v3.14.0^41^ and Platanus-allee v2.2.2^42^, with default parameters. Sequencing data and draft genome assembly statistics are provided in Supplementary Table 10.

### DNA/RNA extraction and sequencing for chromosome-level genomes

The methods for DNA/RNA extraction and sequencing for the three chromosome-level genomes followed procedures described in our previous study^43^. Algal cells were processed by first powdering them in liquid nitrogen. The powdered cells were then mixed with lysis buffer and RNase A, followed by incubation. After centrifugation, the supernatants were subjected to magnetic-bead-based DNA purification and washing. High-quality DNA was obtained and sequenced using both PacBio long-reads and MGI short-reads technologies. Libraries were prepared with specific kits and sequenced on the MGISEQ-2000-PE150 and PacBio Sequel SMRT Cell platforms.

For Hi-C analysis, algal samples were cross-linked, fragmented, and labeled with biotin. DNA fragments were captured using magnetic beads, processed and sequenced on the MGISEQ-2000-PE150 platform to analyze chromatin loci proximity.

Total RNA was extracted using CTAB method. Samples were ground, mixed with CTAB lysis buffer, and subjected to several centrifugation steps. Total RNA with high quality was extracted through cetyltrimethylammonium bromide (CTAB) methods for transcriptome sequencing through MGI and PacBio platform. The mRNA sequencing library (DNBSEQ) was constructed and detected by DNF-471 Standard Sensitivity RNA Analysis Kit (AATI), BGI Optimal two-module mRNA library kit (BGI), BGI Plug-In Adapter Kit (BGI) and Qubit® ssDNA Assay Kit (Invitrogen). The sequencing library (DNBSEQ) was constructed and detected by MGIEasy Universal DNA Library Prep Set (MGI), Qubit™ dsDNA BR Assay Kit (Invitrogen) and Qubit® ssDNA Assay Kit (Invitrogen), and the sequencing strategy was MGISEQ-2000-PE150. The PacBio continuous long reads (CLR) sequencing library was constructed and detected by the SMRTbell Express Template Prep Kit 2.0 (PacBio), Qubit dsDNA HS Assay Kit 2.0 (Invitrogen) and HS Large Fragment 50 KB Analysis Kit (Agilent Technologies). The sequencing strategy was PacBio Sequel SMRT Cell 1 M.

### Genome size estimation and genome assembly

To estimate the sizes of the genomes of the *Pseudo-nitzschia* strains, K-mer analysis was performed using Illumina DNA sequencing data and Jellyfish (v 2.1.4)^44^. For each dataset, k-mers were counted and aggregated (jellyfish count option), and histograms were generated with the “-histo” command. The resulting histograms were then used to estimate genome length and heterozygosity using GenomeScope v 2.0^45^. The genome sizes, repetitive contents, and heterozygosity levels of three *Pseudo-nitzschia* species *P. delicatissima*, *P. pungens*, and *P. multiseries* were estimated by carrying out genome survey analysis using Illumina DNA sequencing results of 27.85 Gb, 44.24 Gb, and 27.98 Gb for *P. delicatissima*, *P. pungens*, and *P. multiseries*, respectively (Supplementary Table 11). The genome sizes of 35 Mb, 78 Mb, and 267 Mb were estimated for *P. delicatissima*, *P. pungens*, and *P. multiseries*, respectively (Supplementary Fig. 1**;** Supplementary Table 2); the heterozygosity levels of *P. delicatissima*, *P. pungens* and *P. multiseries* were estimated to be 1.37%, 0.82% and 0.87%, respectively; the repetitive contents were estimated to be 11.00%, 42.10% and 71.00%, respectively (Supplementary Table 2). Thus, the genome sizes of the three *Pseudo-nitzschia* species varied substantially.

For whole genome assemblies, long reads generated from PacBio Sequel platform were assembled using Mecat2^46^ to generate initial versions of the genome assemblies, which were subsequently polished using Pilon^47^. The contigs were assembled into chromosomes through Hi-C analysis using Juicer^48^ and 3D-DNA^49^ by default parameters. These genome assemblies were further visualized, and error corrected using JucieBox^50^. The completeness of the assembled genomes was evaluated using BUSCO v3 (eukaryota_odb9)^51^.

### Genome annotation

Repeat sequences in the genome of *Pseudo-nitzschia* species were annotated using a comprehensive analysis combining homology-based and de novo prediction methods. The homology-based approach utilized the RepBase v21.12 library^52^, employing RepeatMasker v4.0.7 (http://www.repeatmasker.org/). This strategy enabled the identification of sequences sharing similarity with known repetitive elements. For de novo prediction, the RepeatScout tool^53^, Piler^54^, and LTR_FINDER v1.07^55^ were employed to construct a de novo repeat sequence library. Subsequently, RepeatMasker was applied to predict de novo repeat elements within the genome using the constructed library. To identify tandem repeat sequences, Tandem Repeats Finder v4.09^26^ was used.

The genome sequences were used for homology-based, de novo, and transcriptome-based gene predictions. First, the homologous proteins from seven species including *Arabidopsis thaliana*, *Fragilariopsis cylindrus*, *Phaeodactylum tricornutum*, *Seminavis robusta*, *Thalassiosira pseudonana*, *P. multistriata*, and *Skeletonema marinoi*, were used to identify proteins in the repeat masked *Pseudo-nitzschia* species genome reference sequence with MAKER software (v.2.31.8)^27^. From the homology predictions, a subset of 2000 well-supported genes were selected as a training set. De novo gene prediction software, Augustus^56^ and SNAP^57^, were then trained using this set to improve accuracy in predicting gene structures. To enhance annotation accuracy, RNA-seq and Iso-Seq transcriptomic data were incorporated. RNA-seq data were aligned to the genome using HISAT2 (v2.1.0)^58^, and transcripts were assembled using StringTie (v1.3.4d)^59^. PASA pipeline (https://github.com/PASApipeline/PASApipeline) was subsequently employed for correction and refinement of the transcript information, resulting in a high confidence set of transcripts. The homology-based, de novo, and transcriptome-based were integrated using Maker for a second round of gene structure prediction and consolidation.

Protein-Protein BLAST v2.2.31 was then used to assess putative protein functions in each *Pseudo-nitzschia* species by comparing the protein sequences given by MAKER to the protein sequences from the annotated genomes. The predicted protein coding genes were functionally annotated based on several publicly available databases including Swissprot^60^, TrEMBL (http://www.uniprot.org/), InterPro^61^, GO^62^, KEGG^63^, and NR (http://www.ncbi.nlm.nih.gov/protein/) databases.

The tRNA sequences in the genome were identified utilizing the tRNAscan-SE 1.3.1 software^64^. To annotate rRNA sequences, we performed a BLASTN search against rRNA sequences of a set of closely related species. The alignment results were used to identify and annotate rRNA sequences within the genome. The miRNA and snRNA was predicted using Rfam (v1.0.4)^65^.

### Collinearity analysis

To investigate the differences in chromosome numbers among the three species *P. delicatissima*, *P. pungens*, and *P. multiseries*, MCscanX in JCVI (python -m jcvi.compara.catalog ortholog)^66^ was utilized for pairwise comparisons between the species. To reveal the collinearity relationship between *P. delicatissima*, *P. pungens*, and *P. multiseries*, genome-wide synteny analysis was performed using the MCScanX pipeline within JCVI utility libraries (python -m jcvi.graphics.karyotype)^66^.

### Gene family and evolutionary analysis

Gene families were identified between three *Pseudo-nitzschia* species and other nine species (*A. anophagefferens*, *T. oceanica*, *T. pseudonana*, *S. marinoi*, *S. robusta*, *P. tricornutum*, *F. cylindrus*, *P. multistriata*, and *P. multiseries*) using Orthofinder2^67^. Each of the gene sets from the 12 species was filtered using a condition where, if there were multiple alternatively spliced transcripts in a gene, only the longest transcript was retained. The similarity of protein sequences was assessed by all-versus-all BLASTP with an E-value 1e-6.

A total of 381 single-copy orthologous genes were identified. Protein alignments for individual orthogroup were done by MAFFT^68^. The alignments were processed to remove sites with over 50% gaps and remove sequences shorter than 50% of the alignment length. To infer the species tree, we used both concatenation and multispecies coalescent approach. The concatenated dataset included all the 381 loci and was analyzed using IQ-Tree^69^ with ModelFinder model selection. To assess branch supports, we carried out ultrafast bootstrap and SH-aLRT analyses (both with 1000 replicates).

To estimate the divergence time of different species, the mcmctree program from PAML (v.4.9)^70^ was used. Calibration points for the divergence analysis were obtained from the TimeTree database (http://www.timetree.org/). The resulting phylogenetic tree was presented with 95% highest posterior density (HPD) interval.

Gene family contraction and expansion analysis were performed using the CAFE5^71^ software based on gene family clustering data. A stochastic birth and death model were proposed in CAFE to estimate the λ value.

Pfam domains for the 12 species were identified using InterProscan^72^ and visualized using R packages *pheatmap* and *ggplot2*. Phylogenetic trees of genes, including PF05548 domains, were reconstructed using IQ-TREE^69^ with 1000 bootstrap replications.

### Annotation of DA biosynthesis gene clusters

For the identification of *dab* genes in the chromosome-level genome assemblies of *P. delicatissima*, *P. pungens* and *P. multiseries*, protein sequences of DabA, DabB, DabC, and DabD from *P. multiseries*^8^ were used as queries to search for the candidate genes, *dabA*, *dabB*, *dabC*, and *dabD*.

For other *Pseudo-nitzschia* species, the draft genomes were assembled using SPAdes v3.14.0^41^ and Platanus-allee v2.2.2^42^, with default parameters. BLAST was utilized to probe for *dab* genes with queries mentioned above. Gene annotation was performed using Genewise^73,74^ and ORFfinder (https://www.ncbi.nlm.nih.gov/orffinder/), with the amino acid sequences of *dab* genes from *P. multiseries* serving as a reference. To ensure that no *dab* genes were missed due to incomplete assembly results, we also used the gene sequences or transcript sequences of *dabA*, *dabB*, *dabC*, and *dabD* as queries, using the BWA v0.7.17^75^ MEM algorithm to search for potential genomic sequences containing *dab* genes in assembled genomes.

### Phylogenetic analysis of DA biosynthesis genes

In the phylogenetic analysis of *dab* genes, other than obtaining the published *dab* gene sequences from different *Pseudo-nitzschia* species^8–10^, we also incorporated *radA*, *radC*, *kabA*, and *kabC* from the red algae^28,29^. Protein sequences of the assembled transcripts were obtained by using the TransDecoder program (https://github.com/TransDecoder/TransDecoder). Furthermore, sequences were also acquired by querying the NCBI NR database using *dab* genes as references. Chloroplast genomes (cpDNAs) and ITS (ITS1-5.8S-ITS2) sequences of *Pseudo-nitzschia* were obtained from NCBI database and our previous study^40^. Sequence alignments were performed using MUSCLE^76^, followed by gap removal with trimAl^77^. Maximum Likelihood (ML) trees were constructed with IQ-Tree^69^, incorporating ModelFinder for optimal model selection. Branch supports were evaluated through ultrafast bootstrap, SH-aLRT (both with 1000 replicates), and an Approximate Bayes test. Protein structures were predicted using ColabFold v1.5.2^78^ and visualized with Pymol^79^. Collinearity analysis was performed using Mauve^80^ and Dotter^81^.

### Toxin analysis

The cultivation parameters for *Pseudo-nitzschia* strains followed the conditions described above. Algal cells were harvested during the late-exponential growth phase, approximately 21 days post-inoculation. For DA quantification, cell counts were performed by subsampling 5 mL of algal cells and preserved in Lugol’s iodine solution, samples were kept at 4 °C for subsequent microscopic counts. The collection, preparation, and analytical conditions of different samples refer to the previous study^82^. The DA detection was conducted at the Institute of Oceanology, Chinese Academy of Sciences, using ultra-performance liquid chromatography-electron spray ionization-quadrupole-time of flight-mass spectrometry (UPLC-ESI-Q-TOF-MS) (Bruker, Germany). The DA standard was sourced from the National Marine Environmental Monitoring Center (Standard Material Number: GBW(E)100782). A total of 13 *Pseudo-nitzschia* strains were tested for DA production (Supplementary Table 8), including *P. multiseries* (CNS00159), *P. multistriata* (CNS00142, CNS00781, CNS00965, CNS01237, CNS01424), *P. delicatissima* (CNS00130), *P. americana* (CNS00138), *P. micropora* (CNS00133), *P. pungens* (CNS00141), *P. galaxiae* (CNS01103), *P. brasiliana* (CNS01029), and *Pseudo-nitzschia* sp. (CNS01031).

### Reporting summary

Further information on research design is available in the Nature Portfolio Reporting Summary linked to this article.

## Supplementary information

Supplementary Figs. and tables

Reporting Summary

## Data Availability

The sequencing results (raw data) have been submitted to NCBI, and the BioProject number is PRJNA1054348. The assembled genome has been deposited in the NCBI assembly with the accession number GCA_037355735.1, GCA_037355745.1 and GCA_037355755.1. The genome annotation information has been uploaded to Figshare: 10.6084/m9.figshare.27254001.
