# Supplementary material for: Acquisition and evolution of the neurotoxin domoic acid biosynthesis gene cluster in Pseudo-nitzschia species
Source: Commun Biol. 2024 Oct 23;7:1378. doi: 10.1038/s42003-024-07068-7 (PMC11499653; doi:10.1038/s42003-024-07068-7)
Supplement: Supplementary file 1 — Supplementary Figs. and tables [file 42003_2024_7068_MOESM1_ESM.pdf]

Acquisition and evolution of the neurotoxin domoic acid biosynthesis gene cluster in *Pseudo-nitzschia* species

Ziyan He[a,b,c,d*], Qing Xu[a,b,d,e*], Yang Chen[a,b,c,d], Shuya Liu[a,b,d], Huiyin Song[a,b,d], Hui Wang[a,b,d], Chui Pin Leaw[f], Nansheng Chen[a,b,d #]

[a]CAS Key Laboratory of Marine Ecology and Environmental Sciences, Institute of Oceanology, Chinese Academy of Sciences, Qingdao 266071, China

[b]Laboratory of Marine Ecology and Environmental Science, Qingdao National Laboratory for Marine Science and Technology, Qingdao 266200, China

[c]College of Marine Science, University of Chinese Academy of Sciences, Beijing 10039, China

[d]Center for Ocean Mega-Science, Chinese Academy of Sciences, Qingdao 266071, China

[e]College of Basic Medical Sciences, China Three Gorges University, Yichang 443000, China

[f]Bachok Marine Research Station, Institute of Ocean and Earth Sciences, University of Malaya, 16310 Bachok, Kelantan, Malaysia

*Equal contribution

[#]Corresponding author

E-mail address: chenn@qdio.ac.cn (N. Chen)

Supplementary documents

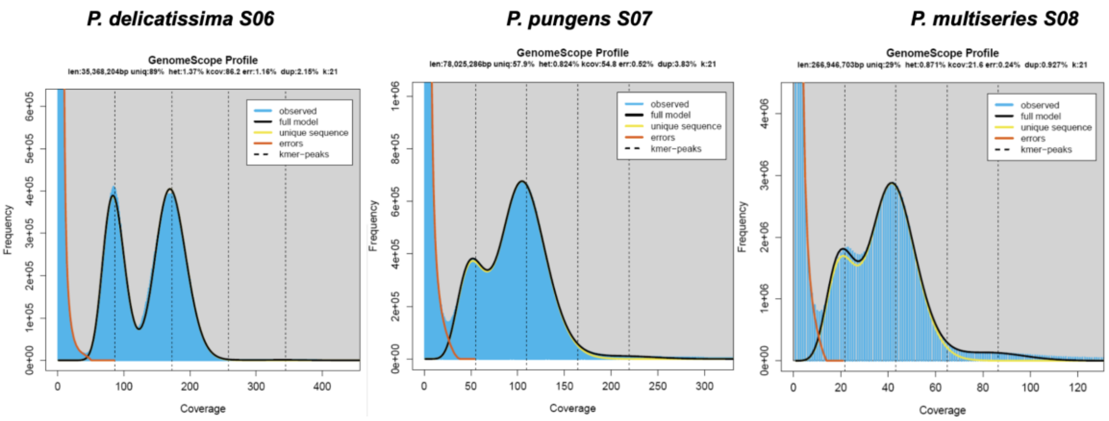

Figure S1. Genome survey analysis of the *Pseudo-nitzschia* species whose whole genomes were constructed in this study.

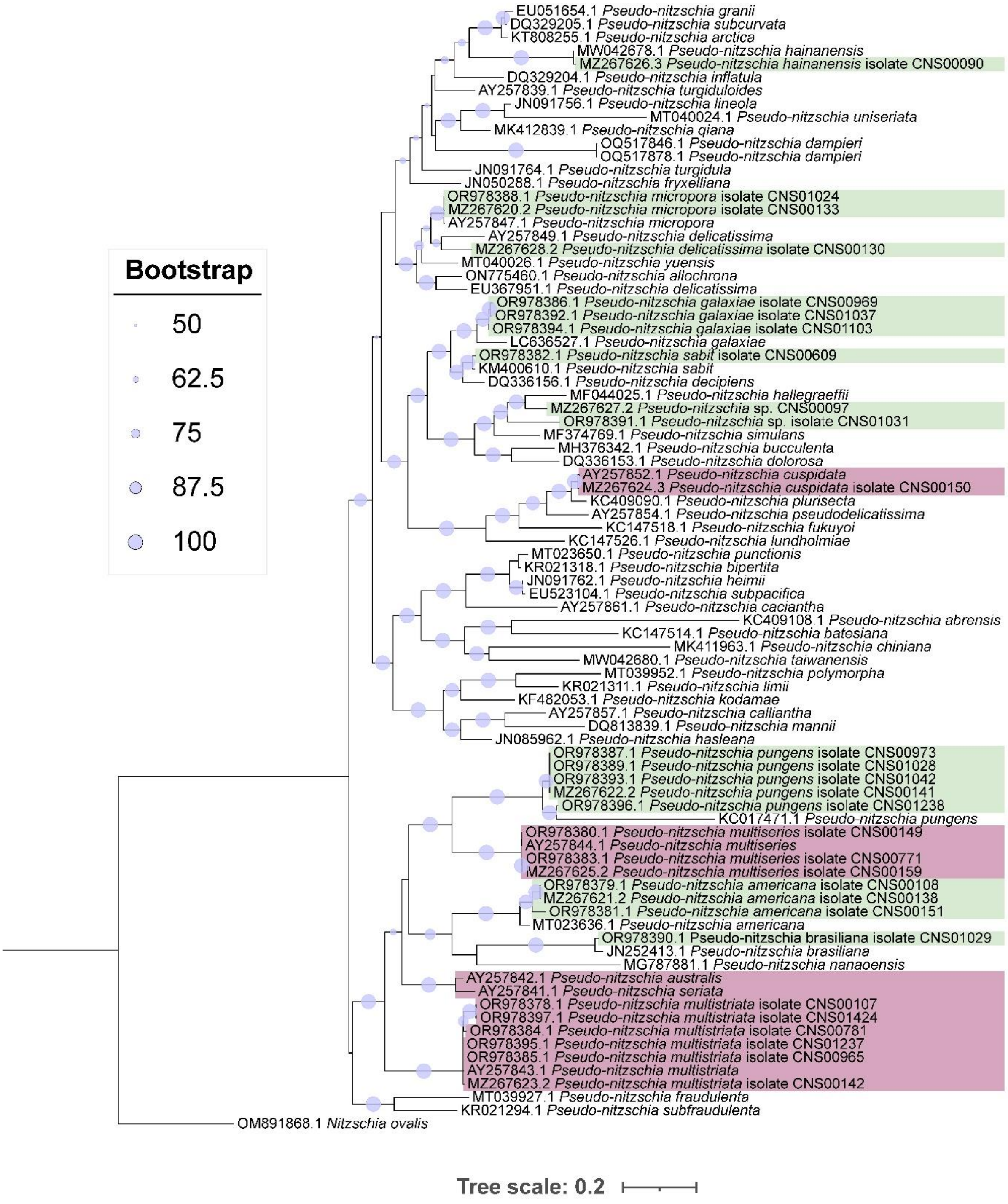

Bootstrap
50
62.5
75
87.5
100
EU051654.1 Pseudo-nitzschia granii
DQ329205.1 Pseudo-nitzschia subcurvata
KT808255.1 Pseudo-nitzschia arctica
MW042678.1 Pseudo-nitzschia hainanensis
MZ267626.3 Pseudo-nitzschia hainanensis isolate CNS00090
DQ329204.1 Pseudo-nitzschia inflatula
AY257839.1 Pseudo-nitzschia turgiduloides
JN091756.1 Pseudo-nitzschia lineola
MT040024.1 Pseudo-nitzschia uniseriata
MK412839.1 Pseudo-nitzschia qiana
OQ517846.1 Pseudo-nitzschia dampieri
OQ517878.1 Pseudo-nitzschia dampieri
JN091764.1 Pseudo-nitzschia turgidula
JN050288.1 Pseudo-nitzschia fryxelliana
OR978388.1 Pseudo-nitzschia micropora isolate CNS01024
MZ267620.2 Pseudo-nitzschia micropora isolate CNS00133
AY257847.1 Pseudo-nitzschia micropora
AY257849.1 Pseudo-nitzschia delicatissima
MZ267628.2 Pseudo-nitzschia delicatissima isolate CNS00130
MT040026.1 Pseudo-nitzschia yuensis
ON775460.1 Pseudo-nitzschia allochrona
EU367951.1 Pseudo-nitzschia delicatissima
OR978386.1 Pseudo-nitzschia galaxiae isolate CNS00969
OR978392.1 Pseudo-nitzschia galaxiae isolate CNS01037
OR978394.1 Pseudo-nitzschia galaxiae isolate CNS01103
LC636527.1 Pseudo-nitzschia galaxiae
OR978382.1 Pseudo-nitzschia sabit isolate CNS00609
KM400610.1 Pseudo-nitzschia sabit
DQ336156.1 Pseudo-nitzschia decipiens
MF044025.1 Pseudo-nitzschia hallegraeffii
MZ267627.2 Pseudo-nitzschia sp. CNS00097
OR978391.1 Pseudo-nitzschia sp. isolate CNS01031
MF374769.1 Pseudo-nitzschia simulans
MH376342.1 Pseudo-nitzschia bucculenta
DQ336153.1 Pseudo-nitzschia dolorosa
AY257852.1 Pseudo-nitzschia cuspidata
MZ267624.3 Pseudo-nitzschia cuspidata isolate CNS00150
KC409090.1 Pseudo-nitzschia plurisecta
AY257854.1 Pseudo-nitzschia pseudodelicatissima
KC147518.1 Pseudo-nitzschia fukuyoi
KC147526.1 Pseudo-nitzschia lundholmiae
MT023650.1 Pseudo-nitzschia punctionis
KR021318.1 Pseudo-nitzschia bipertita
JN091762.1 Pseudo-nitzschia heimii
EU523104.1 Pseudo-nitzschia subpacifica
AY257861.1 Pseudo-nitzschia caciantha
KC409108.1 Pseudo-nitzschia abrensis
KC147514.1 Pseudo-nitzschia batesiana
MK411963.1 Pseudo-nitzschia chiniana
MW042680.1 Pseudo-nitzschia taiwanensis
MT039952.1 Pseudo-nitzschia polymorpha
KR021311.1 Pseudo-nitzschia limii
KF482053.1 Pseudo-nitzschia kodamae
AY257857.1 Pseudo-nitzschia calliantha
DQ813839.1 Pseudo-nitzschia mannii
JN085962.1 Pseudo-nitzschia hasleana
OR978387.1 Pseudo-nitzschia pungens isolate CNS00973
OR978389.1 Pseudo-nitzschia pungens isolate CNS01028
OR978393.1 Pseudo-nitzschia pungens isolate CNS01042
MZ267622.2 Pseudo-nitzschia pungens isolate CNS00141
OR978396.1 Pseudo-nitzschia pungens isolate CNS01238
KC017471.1 Pseudo-nitzschia pungens
OR978380.1 Pseudo-nitzschia multiseries isolate CNS00149
AY257844.1 Pseudo-nitzschia multiseries
OR978383.1 Pseudo-nitzschia multiseries isolate CNS00771
MZ267625.2 Pseudo-nitzschia multiseries isolate CNS00159
OR978379.1 Pseudo-nitzschia americana isolate CNS00108
MZ267621.2 Pseudo-nitzschia americana isolate CNS00138
OR978381.1 Pseudo-nitzschia americana isolate CNS00151
MT023636.1 Pseudo-nitzschia americana
OR978390.1 Pseudo-nitzschia brasiliana isolate CNS01029
JN252413.1 Pseudo-nitzschia brasiliana
MG787881.1 Pseudo-nitzschia nanaoensis
AY257842.1 Pseudo-nitzschia australis
AY257841.1 Pseudo-nitzschia seriata
OR978378.1 Pseudo-nitzschia multistriata isolate CNS00107
OR978397.1 Pseudo-nitzschia multistriata isolate CNS01424
OR978384.1 Pseudo-nitzschia multistriata isolate CNS00781
OR978395.1 Pseudo-nitzschia multistriata isolate CNS01237
OR978385.1 Pseudo-nitzschia multistriata isolate CNS00965
AY257843.1 Pseudo-nitzschia multistriata
MZ267623.2 Pseudo-nitzschia multistriata isolate CNS00142
MT039927.1 Pseudo-nitzschia fraudulenta
KR021294.1 Pseudo-nitzschia subfraudulenta
OM891868.1 Nitzschia ovalis
Tree scale: 0.2

Figure S2 Maximum Likelihood (ML) trees based on ITS1-5.8S-ITS2 sequences. Only bootstrap values >50 % and aBayes support >0.7 are marked with circles. The bootstrap values are denoted by the relative size of the circles. *Pseudo-nitzschia* species that possess the *dab* gene cluster in previous studies and this study are highlighted in purple, whereas those without *dab* genes in this study are indicated in green.

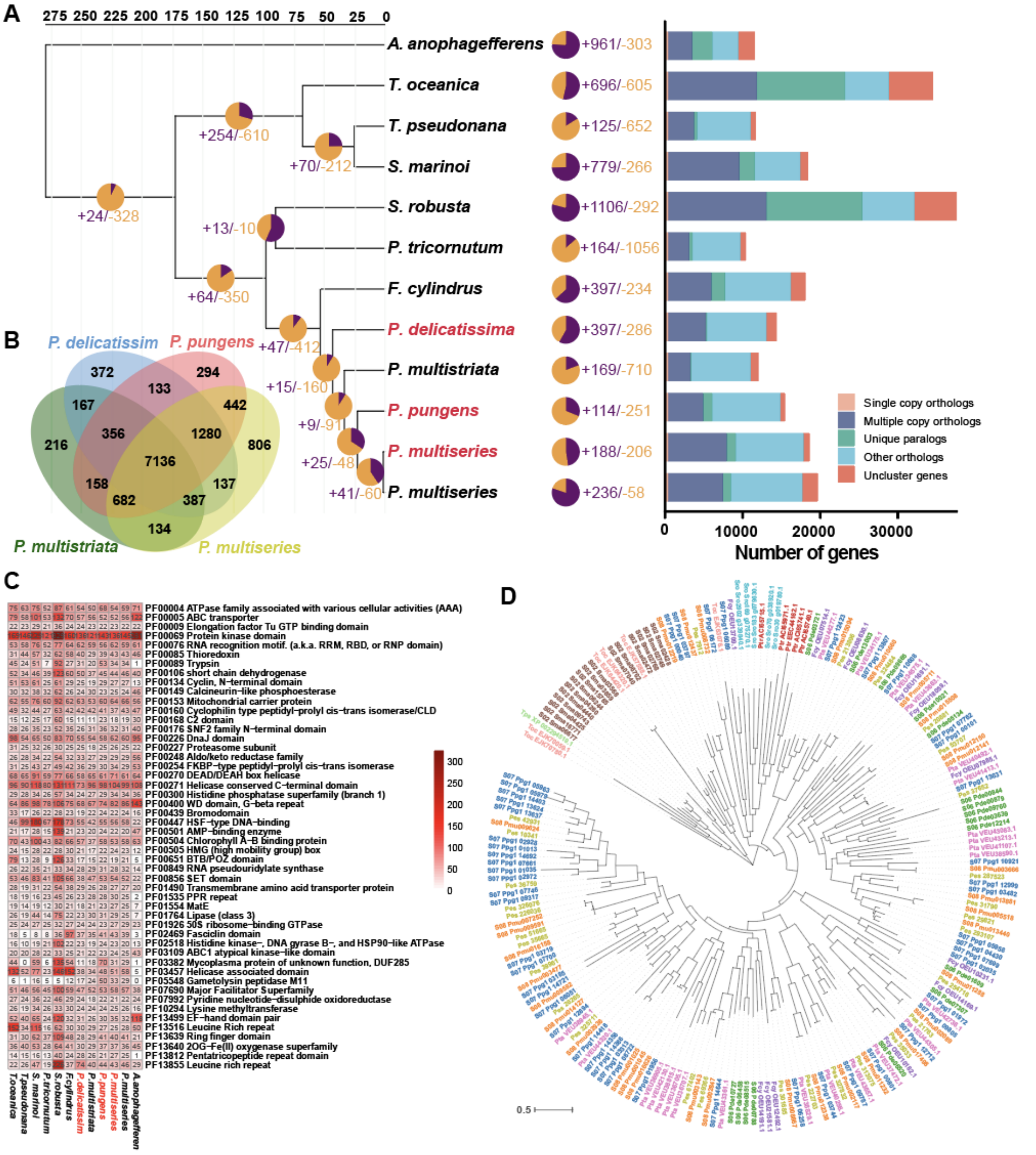

Figure S3 Comparative analysis and evolution of gene families

A. Phylogenetic analysis of three *Pseudo-nitzschia* species with nine other phytoplankton species. B. Comparative analysis of gene families of four *Pseudo-nitzschia* species. C. Comparative analysis of Pfam gene families of three Pseudo-nitzschia species with nine other phytoplankton species. D. Phylogenetic analysis of genes including PF05548 domains.

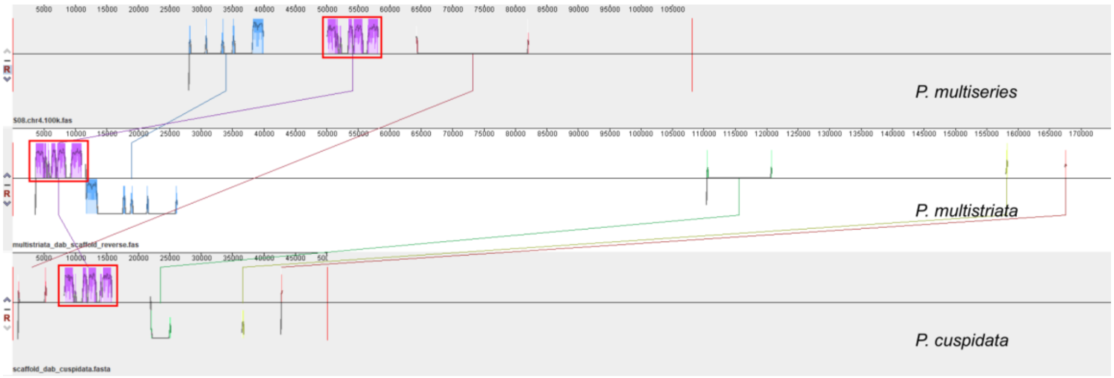

Figure S4. Comparative analysis of genomic segments harboring the *dab* gene clusters in the genome of the three *Pseudo-nitzschia* species, including *P*. *multiseries* (strain CNS00149, Chr4), *P*. *multistriata* (Accession: CAACVS010000654.1), *P*. *cuspidata* (strain CNS00150). Sequences of *dab* gene clusters were colored in purple and marked with red box.

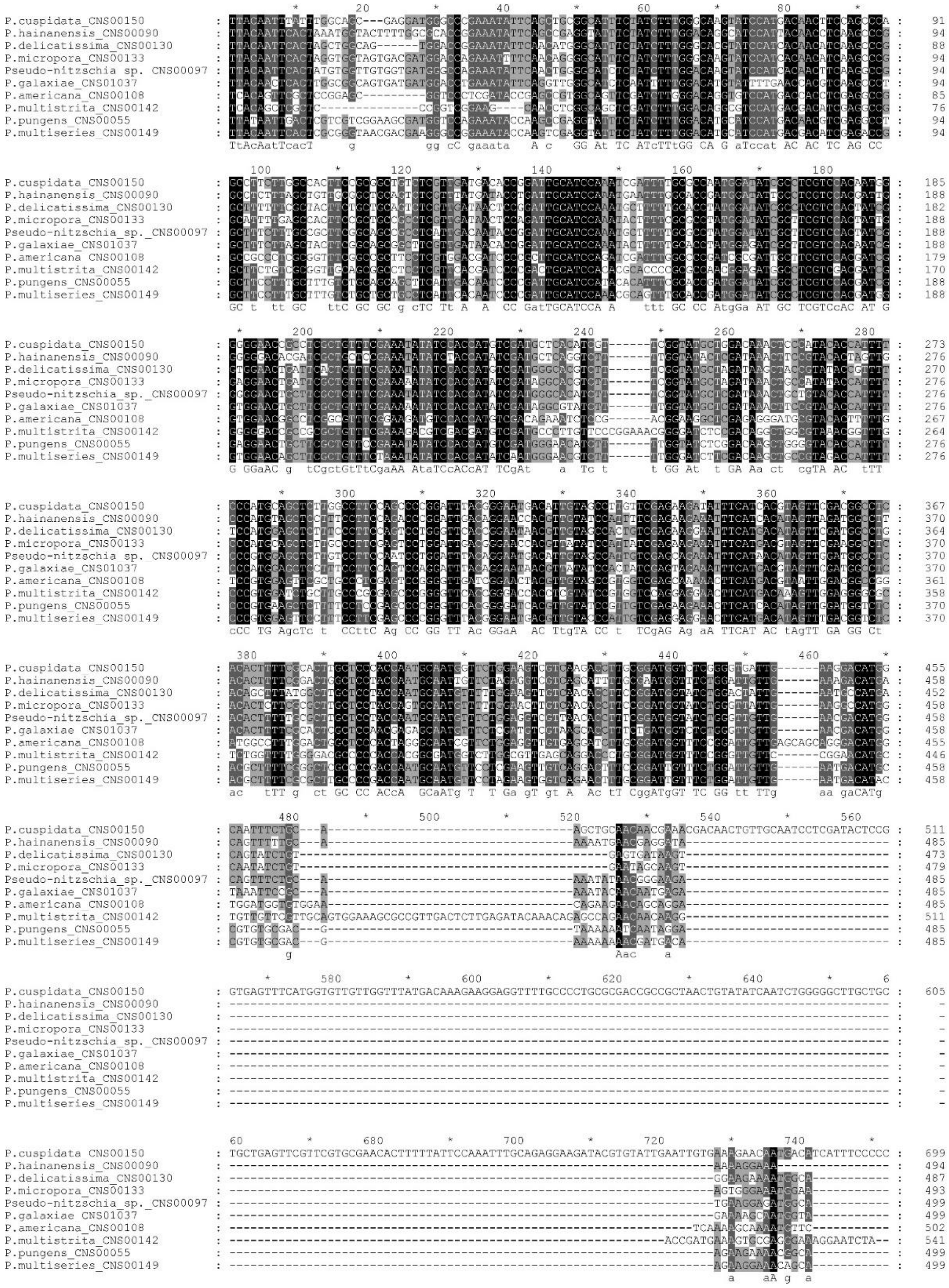
P.cuspidata_CNS00150
P.hainanensis_CNS00090
P.delicatissima_CNS00130
P.micropora_CNS00133
Pseudo-nitzschia_sp._CNS00097
P.galaxiae_CNS01037
P.americana_CNS00108
P.multistrita_CNS00142
P.pungens_CNS00055
P.multiseries_CNS00149

```
                                      760         *        780         *        800         *        820         *        840
P.cuspidata_CNS00150          : CCGCATTGTTTCCCTCTTTTCATCGGAATCGCCTGGTAGTAGAATTTTTGTTTTTCCGTGGTACACGTGCGTACCCCATGCGCGAGGGGCTGC :  793
P.hainanensis_CNS00090        : ------------------------------------------------------------------ATAAGCTC-------------------- :  502
P.delicatissima_CNS00130      : ------------------------------------------------------------------ATCGGTGC-------------------- :  495
P.micropora_CNS00133          : ------------------------------------------------------------------AGTGGAGC-------------------- :  501
Pseudo-nitzschia_sp._CNS00097 : ------------------------------------------------------------------ATCGGTGA-------------------- :  507
P.galaxiae_CNS01037           : ------------------------------------------------------------------ATGGGTAC-------------------- :  507
P.americana_CNS00108          : ------------------------------------------------------------------TTTGGTAG-------------------- :  510
P.multistrita_CNS00142        : ------------------------------------------------------------------GCTGGGAA-------------------- :  549
P.pungens_CNS00055            : ------------------------------------------------------------------ATCGGGAA-------------------- :  507
P.multiseries_CNS00149        : ------------------------------------------------------------------ATGTGGAA-------------------- :  507
                                                                                                   t gG

                                  *        860         *        880         *        900         *        920         *        940
P.cuspidata_CNS00150          : ATACTCTTATCCCCCGTAAAACTCTCATCTCTTGTCCTCGACAGAAAAAAGTAGGCGAAACGAAACGGAATTTTTTGACTTGTCGTTGGCGGT :  887
P.hainanensis_CNS00090        : ---------------------------------------------------------------------------------------------- :    -
P.delicatissima_CNS00130      : --------------------------------------------------------------AAAG---------------------------- :  499
P.micropora_CNS00133          : --------------------------------------------------------------AAAG---------------------------- :  505
Pseudo-nitzschia_sp._CNS00097 : --------------------------------------------------------------AAAA---------------------------- :  511
P.galaxiae_CNS01037           : --------------------------------------------------------------AAAC---------------------------- :  511
P.americana_CNS00108          : ---------------------------------------------------------------------------------------------- :    -
P.multistrita_CNS00142        : ---------------------------------------------------------------------------------------------- :    -
P.pungens_CNS00055            : --------------------------------------------------------------GAAT---------------------------- :  511
P.multiseries_CNS00149        : ---------------------------------------------------------------------------------------------- :    -

                                        *        960         *        980         *       1000         *       1020         *
P.cuspidata_CNS00150          : TTTCGTTGCTTGTGGTATGCTGGAGGGCAAGATGGTCTCCAGTCTTCTAGTAGAGTAGTTTAATCGAAATAAATTTAAAATAAAGATTCATGAT :  981
P.hainanensis_CNS00090        : ---------------------------------------------------------------------------------------------- :    -
P.delicatissima_CNS00130      : ---------------------------------------------------------------------------------------------- :    -
P.micropora_CNS00133          : ---------------------------------------------------------------------------------------------- :    -
Pseudo-nitzschia_sp._CNS00097 : ---------------------------------------------------------------------------------------------- :    -
P.galaxiae_CNS01037           : ---------------------------------------------------------------------------------------------- :    -
P.americana_CNS00108          : ---------------------------------------------------------------------------------------------- :    -
P.multistrita_CNS00142        : ---------------------------------------------------------------------------------------------- :    -
P.pungens_CNS00055            : ---------------------------------------------------------------------------------------------- :    -
P.multiseries_CNS00149        : ---------------------------------------------------------------------------------------------- :    -

                                   1040         *       1060         *       1080         *       1100         *       1120
P.cuspidata_CNS00150          : GTAATAATAGTAGTGTAGATGCTGTTGTGGTTTTGGATAGTGTACTAGATTAATTGTTATTTTTTCCCAAATATTGTTTTTTTCTACATCACGC : 1075
P.hainanensis_CNS00090        : ---------------------------------------------------------------------------------------------- :    -
P.delicatissima_CNS00130      : ---------------------------------------------------------------------------------------------- :    -
P.micropora_CNS00133          : ---------------------------------------------------------------------------------------------- :    -
Pseudo-nitzschia_sp._CNS00097 : ---------------------------------------------------------------------------------------------- :    -
P.galaxiae_CNS01037           : ---------------------------------------------------------------------------------------------- :    -
P.americana_CNS00108          : ---------------------------------------------------------------------------------------------- :    -
P.multistrita_CNS00142        : ---------------------------------------------------------------------------------------------- :    -
P.pungens_CNS00055            : ---------------------------------------------------------------------------------------------- :    -
P.multiseries_CNS00149        : ---------------------------------------------------------------------------------------------- :    -

                                  *       1140         *       1160         *       1180         *       1200         *       1220
P.cuspidata_CNS00150          : GTTCTGTCGTCCGGGAAAGACTGCTTTTCTCCAGCAGCAGCGTTGAGAACCTGCCTGGATCTTTCTTTGTATTTAGTGAT-GTCCTGTAATCTT : 1168
P.hainanensis_CNS00090        : --------------------------------------------------------------------------AATGGCTGTGGT-------- :  514
P.delicatissima_CNS00130      : --------------------------------------------------------------------------ATTGACGATCCGGTA----- :  514
P.micropora_CNS00133          : --------------------------------------------------------------------------ATTGACAGTCCAGTA----- :  520
Pseudo-nitzschia_sp._CNS00097 : --------------------------------------------------------------------------ATGGATAATCGTGCA----- :  526
P.galaxiae_CNS01037           : --------------------------------------------------------------------------AGTGACAGTCCTGTA----- :  526
P.americana_CNS00108          : -------------------------------------------------------------------------TAATGACGCTTGGATG----- :  526
P.multistrita_CNS00142        : --------------------------------------------------------------------------AAAGACTCTCGTCGGC---- :  565
P.pungens_CNS00055            : --------------------------------------------------------------------------AACGGTAATGGGGTG----- :  526
P.multiseries_CNS00149        : -------------------------------------------------------------------------TAGGATCGTTAAAGTGTTT-- :  526
                                                                                                          A  g    T

                                        *       1240         *       1260         *       1280         *       1300         *
P.cuspidata_CNS00150          : TTGTTTTGTGAGTAGTGATGTGTTATTTTGTGATATTCTCCAACCAATAATCCAAGTCTGCCATGGAAGACTGATCCGACAACATCCGACGTCG : 1262
P.hainanensis_CNS00090        : ---------------------------------------------------------------------------------------------- :    -
P.delicatissima_CNS00130      : ---------------------------------------------------------------------------------------------- :    -
P.micropora_CNS00133          : ---------------------------------------------------------------------------------------------- :    -
Pseudo-nitzschia_sp._CNS00097 : ---------------------------------------------------------------------------------------------- :    -
P.galaxiae_CNS01037           : ---------------------------------------------------------------------------------------------- :    -
P.americana_CNS00108          : ---------------------------------------------------------------------------------------------- :    -
P.multistrita_CNS00142        : ---------------------------------------------------------------------------------------------- :    -
P.pungens_CNS00055            : ---------------------------------------------------------------------------------------------- :    -
P.multiseries_CNS00149        : ---------------------------------------------------------------------------------------------- :    -

                                 1320         *       1340         *       1360         *       1380         *       1400         *
P.cuspidata_CNS00150          : TCGAAGTTGGAAAATTCGGTTTTCATTCCGTCTCTTGCTCCGTGCAAGCCAAGCCGTATACTTCTGTTCAAGCAAGCATCCTTTCATGAAGCTT : 1356
P.hainanensis_CNS00090        : ------------------ATTTGCAT-------------------------------------------------------------------- :  522
P.delicatissima_CNS00130      : ------------------ACTTTCAT-------------------------------------------------------------------- :  522
P.micropora_CNS00133          : ------------------ACTTTCAT-------------------------------------------------------------------- :  528
Pseudo-nitzschia_sp._CNS00097 : ------------------ATTTTCAT-------------------------------------------------------------------- :  534
P.galaxiae_CNS01037           : ------------------GTTTTCAT-------------------------------------------------------------------- :  534
P.americana_CNS00108          : ------------------GTGTGCAT-------------------------------------------------------------------- :  534
P.multistrita_CNS00142        : ------------------CTCTGTAT-------------------------------------------------------------------- :  573
P.pungens_CNS00055            : ------------------ATTCGCAT-------------------------------------------------------------------- :  534
P.multiseries_CNS00149        : ------------------ATTCGCAT-------------------------------------------------------------------- :  534
                                                   ttt cAT

                                       1420         *       1440         *       1460         *       1480         *       1500
P.cuspidata_CNS00150          : AAAGCTTCTCGGAAAGGGATTTGGTCATCTCCTCCTCCAAGTATGCCTTCATCAATGGCTTGTGGTCACGAGCGTAGCCGTTCTCGACGCAGCA : 1450
P.hainanensis_CNS00090        : ---------------------------------------------------------------------------------------------- :    -
P.delicatissima_CNS00130      : ---------------------------------------------------------------------------------------------- :    -
P.micropora_CNS00133          : ---------------------------------------------------------------------------------------------- :    -
Pseudo-nitzschia_sp._CNS00097 : ---------------------------------------------------------------------------------------------- :    -
P.galaxiae_CNS01037           : ---------------------------------------------------------------------------------------------- :    -
P.americana_CNS00108          : ---------------------------------------------------------------------------------------------- :    -
P.multistrita_CNS00142        : ---------------------------------------------------------------------------------------------- :    -
P.pungens_CNS00055            : ---------------------------------------------------------------------------------------------- :    -
P.multiseries_CNS00149        : ---------------------------------------------------------------------------------------------- :    -
```

```
                                     *      1520         *      1540         *      1560         *      1580         *      16
P.cuspidata_CNS00150          : CACCTCGTGCAAGACATACCCAATCAAGGATTCTTCTCGGGTCTTGATCATGCGGGCAAGTGCAAGTCGTTCTTCCTCGTCGGCCGCCGATGCG : 1544
P.hainanensis_CNS00090        : ---------------------------------------------------------GATTGTCAC---------------------------- :  531
P.delicatissima_CNS00130      : ---------------------------------------------------------TTTTGTAGC---------------------------- :  531
P.micropora_CNS00133          : ---------------------------------------------------------TGTTGAAAC---------------------------- :  537
Pseudo-nitzschia sp. CNS00097 : ---------------------------------------------------------GCTTGCCAAC--------------------------- :  544
P.galaxiae_CNS01037           : ---------------------------------------------------------AATTGCAAGTG-------------------------- :  545
P.americana_CNS00108          : ---------------------------------------------------------------------------------------------- :    -
P.multistrita_CNS00142        : ---------------------------------------------------------CGTCACAAGCATGCTGC-------------------- :  590
P.pungens_CNS00055            : ---------------------------------------------------------TGTTGCT------------------------------ :  541
P.multiseries_CNS00149        : ---------------------------------------------------------AGTCGCTGTG--------------------------- :  544
                                                                                           t g

                                00         *      1620         *      1640         *      1660         *      1680         *
P.cuspidata_CNS00150          : AGAATCTCCTTTTTGAGCTCCAAAAAGGCTTGCATCTTTTCGTAAGCGTGGCCGACGACAAGATCGCAGGAGGCGGCAAAGTTCAAACTTGGGT : 1638
P.hainanensis_CNS00090        : ---------------------------------------------------------------------------------------------- :    -
P.delicatissima_CNS00130      : ---------------------------------------------------------------------------------------------- :    -
P.micropora_CNS00133          : ---------------------------------------------------------------------------------------------- :    -
Pseudo-nitzschia_sp._CNS00097 : ---------------------------------------------------------------------------------------------- :    -
P.galaxiae_CNS01037           : ---------------------------------------------------------------------------------------------- :    -
P.americana_CNS00108          : ---------------------------------------------------------------------------------------------- :    -
P.multistrita_CNS00142        : ---------------------------------------------------------------------------------------------- :    -
P.pungens_CNS00055            : ---------------------------------------------------------------------------------------------- :    -
P.multiseries_CNS00149        : ---------------------------------------------------------------------------------------------- :    -

                                    1700         *      1720         *      1740         *      1760         *      1780
P.cuspidata_CNS00150          : CGAGGTCCATGAGAATAAAGACCGAGTTGAAGGGATCGTCGTCGGTTCTCTCGCGCTCGCTTGTGCACACGTCGTTGAAGTAGGTGATGATCTC : 1732
P.hainanensis_CNS00090        : ---------------------------------------------------------------------------------------------- :    -
P.delicatissima_CNS00130      : ---------------------------------------------------------------------------------------------- :    -
P.micropora_CNS00133          : ---------------------------------------------------------------------------------------------- :    -
Pseudo-nitzschia_sp._CNS00097 : ---------------------------------------------------------------------------------------------- :    -
P.galaxiae_CNS01037           : ---------------------------------------------------------------------------------------------- :    -
P.americana_CNS00108          : ---------------------------------------------------------------------------------------------- :    -
P.multistrita_CNS00142        : ---------------------------------------------------------------------------------------------- :    -
P.pungens_CNS00055            : ---------------------------------------------------------------------------------------------- :    -
P.multiseries_CNS00149        : ---------------------------------------------------------------------------------------------- :    -

                                   *      1800         *      1820         *      1840         *      1860         *      1880
P.cuspidata_CNS00150          : GGAACAACGATTTTCCAATTCCATAAAGAGCGGGTCGGTGTACGCCTTGTAGGTGAAATGTTCGTGCGTCAAGTCCGAGACGGCGTGGGCCATG : 1826
P.hainanensis_CNS00090        : ---------------------------------------------------------------------------------------------- :    -
P.delicatissima_CNS00130      : ---------------------------------------------------------------------------------------------- :    -
P.micropora_CNS00133          : ---------------------------------------------------------------------------------------------- :    -
Pseudo-nitzschia sp. CNS00097 : ---------------------------------------------------------------------------------------------- :    -
P.galaxiae_CNS01037           : ---------------------------------------------------------------------------------------------- :    -
P.americana_CNS00108          : ---------------------------------------------------------------------------------------------- :    -
P.multistrita_CNS00142        : ---------------------------------------------------------------------------------------------- :    -
P.pungens_CNS00055            : ---------------------------------------------------------------------------------------------- :    -
P.multiseries_CNS00149        : ---------------------------------------------------------------------------------------------- :    -

                                         *      1900         *      1920         *      1940         *      1960         *
P.cuspidata_CNS00150          : GGGCTGGCGGTAACCTTGTCACGGAATTCGTCGTATTCTTGGAGGGATAGAGTGAAACTCTTGTCACCTCGGATGTGGATCTCATCCTTGACAC : 1920
P.hainanensis_CNS00090        : ---------------------------------------------------------------------------------------------- :    -
P.delicatissima_CNS00130      : ---------------------------------------------------------------------------------------------- :    -
P.micropora_CNS00133          : ---------------------------------------------------------------------------------------------- :    -
Pseudo-nitzschia_sp._CNS00097 : ---------------------------------------------------------------------------------------------- :    -
P.galaxiae_CNS01037           : ---------------------------------------------------------------------------------------------- :    -
P.americana_CNS00108          : ---------------------------------------------------------------------------------------------- :    -
P.multistrita_CNS00142        : ---------------------------------------------------------------------------------------------- :    -
P.pungens_CNS00055            : ---------------------------------------------------------------------------------------------- :    -
P.multiseries_CNS00149        : ---------------------------------------------------------------------------------------------- :    -

                                  1980         *      2000         *      2020         *      2040         *      2060
P.cuspidata_CNS00150          : CCTTGATGTATCGGATGACTTCCCAGAGAACTTGGGTAATGTTCTTGGTGGGGGCGAATTGTTCGGCAATGTAGGCGCAAATGGTCTTGATGAG : 2014
P.hainanensis_CNS00090        : ---------------------------------------------------------------------------------------------- :    -
P.delicatissima_CNS00130      : ---------------------------------------------------------------------------------------------- :    -
P.micropora_CNS00133          : ---------------------------------------------------------------------------------------------- :    -
Pseudo-nitzschia_sp._CNS00097 : ---------------------------------------------------------------------------------------------- :    -
P.galaxiae_CNS01037           : ---------------------------------------------------------------------------------------------- :    -
P.americana_CNS00108          : ---------------------------------------------------------------------------------------------- :    -
P.multistrita_CNS00142        : ---------------------------------------------------------------------------------------------- :    -
P.pungens_CNS00055            : ---------------------------------------------------------------------------------------------- :    -
P.multiseries_CNS00149        : ---------------------------------------------------------------------------------------------- :    -

                                 *      2080         *      2100         *      2120         *      2140         *      2160
P.cuspidata_CNS00150          : GGGCATTTGCGCGGGAGTTGCGGCTTTGTATTTGTCGGCCAAAACAATCTTGCCGTCAACCTTTTGGAAGTAGGGCTGGTAAAATTCCAAGAAA : 2108
P.hainanensis_CNS00090        : ---------------------------------------------------------------------------------------------- :    -
P.delicatissima_CNS00130      : ---------------------------------------------------------------------------------------------- :    -
P.micropora_CNS00133          : ---------------------------------------------------------------------------------------------- :    -
Pseudo-nitzschia_sp._CNS00097 : ---------------------------------------------------------------------------------------------- :    -
P.galaxiae_CNS01037           : ---------------------------------------------------------------------------------------------- :    -
P.americana_CNS00108          : ---------------------------------------------------------------------------------------------- :    -
P.multistrita_CNS00142        : ---------------------------------------------------------------------------------------------- :    -
P.pungens_CNS00055            : ---------------------------------------------------------------------------------------------- :    -
P.multiseries_CNS00149        : ---------------------------------------------------------------------------------------------- :    -

                                       *      2180         *      2200         *      2220         *      2240         *
P.cuspidata_CNS00150          : TCCCAAATGCGATTCAAGTCTTCCTCGTCGTCGGGTTCGAAGACACTCAAGAATTCGTTCAAGTAAATCACAATGTCGTCTTCGAGAATGTCGA : 2202
P.hainanensis_CNS00090        : ---------------------------------------------------------------------------------------------- :    -
P.delicatissima_CNS00130      : ---------------------------------------------------------------------------------------------- :    -
P.micropora_CNS00133          : ---------------------------------------------------------------------------------------------- :    -
Pseudo-nitzschia_sp._CNS00097 : ---------------------------------------------------------------------------------------------- :    -
P.galaxiae_CNS01037           : ---------------------------------------------------------------------------------------------- :    -
P.americana_CNS00108          : ---------------------------------------------------------------------------------------------- :    -
P.multistrita_CNS00142        : ---------------------------------------------------------------------------------------------- :    -
P.pungens_CNS00055            : ---------------------------------------------------------------------------------------------- :    -
P.multiseries_CNS00149        : ---------------------------------------------------------------------------------------------- :    -
```

```
                                 2260       *      2280       *      2300       *      2320       *      2340       *
P.cuspidata_CNS00150            : CGAGGTCGGATTGAATCTCCTCTTCGGGAAGCTTGAGGTGGGCGGTTTGGTCATTGATGTAGTACATCATGGAATAAAAATACGCGACAATTTG :  2296
P.hainanensis_CNS00090          : ---------------------------------------------------------------------------------------------- :     -
P.delicatissima_CNS00130        : ---------------------------------------------------------------------------------------------- :     -
P.micropora_CNS00133            : ---------------------------------------------------------------------------------------------- :     -
Pseudo-nitzschia sp. CNS00097   : ---------------------------------------------------------------------------------------------- :     -
P.galaxiae_CNS01037             : ---------------------------------------------------------------------------------------------- :     -
P.americana_CNS00108            : ---------------------------------------------------------------------------------------------- :     -
P.multistrita_CNS00142          : ---------------------------------------------------------------------------------------------- :     -
P.pungens_CNS00055              : ---------------------------------------------------------------------------------------------- :     -
P.multiseries_CNS00149          : ---------------------------------------------------------------------------------------------- :     -

                                        2360       *      2380       *      2400       *      2420       *      2440
P.cuspidata_CNS00150            : CGTATCGCCATAGCTTGAGGCAGGAGTGCAAAGCGCAGAGGTTTTCTCGCTGTTGATGCGGTTGTATTTCTCCATGGTGGTATCGTACATCGAA :  2390
P.hainanensis_CNS00090          : ---------------------------------------------------------------------------------------------- :     -
P.delicatissima_CNS00130        : ---------------------------------------------------------------------------------------------- :     -
P.micropora_CNS00133            : ---------------------------------------------------------------------------------------------- :     -
Pseudo-nitzschia_sp._CNS00097   : ---------------------------------------------------------------------------------------------- :     -
P.galaxiae_CNS01037             : ---------------------------------------------------------------------------------------------- :     -
P.americana CNS00108            : ---------------------------------------------------------------------------------------------- :     -
P.multistrita_CNS00142          : ---------------------------------------------------------------------------------------------- :     -
P.pungens_CNS00055              : ---------------------------------------------------------------------------------------------- :     -
P.multiseries_CNS00149          : ---------------------------------------------------------------------------------------------- :     -

                                      *      2460       *      2480       *      2500       *      2520       *      25
P.cuspidata_CNS00150            : GCACGCTCGTCACGTGAACCGTAGAGATCCATGTAGTGGGTGCTGATCTTGGGCTCGAATGGGCAGTAGAACTTGACGTCGATGCTTTCGCCAG :  2484
P.hainanensis_CNS00090          : ------------------------------------------------------------------------------CTGGTTTGTGAAGAG :   547
P.delicatissima_CNS00130        : ------------------------------------------------------------------------------TCAAT----------- :   536
P.micropora_CNS00133            : ------------------------------------------------------------------------------TCGAT----------- :   542
Pseudo-nitzschia_sp._CNS00097   : ------------------------------------------------------------------------------TTGAT----------- :   549
P.galaxiae_CNS01037             : ------------------------------------------------------------------------------TCGATCG--------- :   552
P.americana_CNS00108            : ------------------------------------------------------------------------------TCTAT----------- :   539
P.multistrita CNS00142          : ------------------------------------------------------------------------------TCGATC---------- :   596
P.pungens_CNS00055              : ------------------------------------------------------------------------------TCGAGTACCTA----- :   552
P.multiseries_CNS00149          : ------------------------------------------------------------------------------GAGATTT--------- :   551
                                                                                                              t gat

                                  40       *      2560       *      2580       *      2600       *      2620       *
P.cuspidata_CNS00150            : CGCACATCGTCTTGATGGGATAATAGTAGTCTCCCTTGTCGTAGAACTTCCCCTTCAAGAAGTCGACCATCAAGTACAGGTCCTTGGGTTCGGT :  2578
P.hainanensis_CNS00090          : GGAT------------------------------------------------------------------------------------------ :   551
P.delicatissima_CNS00130        : ---------------------------------------------------------------------------------------------- :     -
P.micropora_CNS00133            : ---------------------------------------------------------------------------------------------- :     -
Pseudo-nitzschia sp. CNS00097   : ---------------------------------------------------------------------------------------------- :     -
P.galaxiae_CNS01037             : ---------------------------------------------------------------------------------------------- :     -
P.americana_CNS00108            : ---------------------------------------------------------------------------------------------- :     -
P.multistrita_CNS00142          : ---------------------------------------------------------------------------------------------- :     -
P.pungens_CNS00055              : ---------------------------------------------------------------------------------------------- :     -
P.multiseries_CNS00149          : ---------------------------------------------------------------------------------------------- :     -

                                     2640       *      2660       *      2680       *      2700       *      2720
P.cuspidata CNS00150            : CAACTTGAGCCCGGCGTCTTTGATGCGGGAGAGGGCTTCGGCGGGGCTTTCTTCCGTAATGGAACTTGGGGTGCTCAATGCATTGAGCGAGATC :  2672
P.hainanensis_CNS00090          : ---------------------------------------------------------------------------------------------- :     -
P.delicatissima_CNS00130        : ---------------------------------------------------------------------------------------------- :     -
P.micropora_CNS00133            : ---------------------------------------------------------------------------------------------- :     -
Pseudo-nitzschia_sp._CNS00097   : ---------------------------------------------------------------------------------------------- :     -
P.galaxiae CNS01037             : ---------------------------------------------------------------------------------------------- :     -
P.americana_CNS00108            : ---------------------------------------------------------------------------------------------- :     -
P.multistrita_CNS00142          : ---------------------------------------------------------------------------------------------- :     -
P.pungens_CNS00055              : ---------------------------------------------------------------------------------------------- :     -
P.multiseries_CNS00149          : ---------------------------------------------------------------------------------------------- :     -

                                     *      2740       *      2760       *      2780       *      2800       *      2820
P.cuspidata_CNS00150            : TGGTTCTTCGAGAGGGATCGAGGAGTGAATGCCTGAAGAGAAGTCACGACGACGACAACGAAAAGGCGTGTTAGTGAAAAATTCATTAAAAGGA :  2766
P.hainanensis_CNS00090          : ---------------------------------------------------------------------------------------------- :     -
P.delicatissima_CNS00130        : ---------------------------------------------------------------------------------------------- :     -
P.micropora_CNS00133            : ---------------------------------------------------------------------------------------------- :     -
Pseudo-nitzschia_sp._CNS00097   : ---------------------------------------------------------------------------------------------- :     -
P.galaxiae_CNS01037             : ---------------------------------------------------------------------------------------------- :     -
P.americana_CNS00108            : ---------------------------------------------------------------------------------------------- :     -
P.multistrita_CNS00142          : ---------------------------------------------------------------------------------------------- :     -
P.pungens_CNS00055              : ---------------------------------------------------------------------------------------------- :     -
P.multiseries_CNS00149          : ---------------------------------------------------------------------------------------------- :     -

                                        *      2840       *      2860       *      2880       *      2900       *
P.cuspidata_CNS00150            : AATGACCTCAAAGTTTATCCATGGTAAAGAAGATTCCTTTTTATTTCACTTACATTCTGTGGAGATACAGTGAAGGCATTGAGAGAAGTCACGA :  2860
P.hainanensis_CNS00090          : ---------------------------------------------------------------------------------------------- :     -
P.delicatissima_CNS00130        : ---------------------------------------------------------------------------------------------- :     -
P.micropora_CNS00133            : ---------------------------------------------------------------------------------------------- :     -
Pseudo-nitzschia sp. CNS00097   : ---------------------------------------------------------------------------------------------- :     -
P.galaxiae_CNS01037             : ---------------------------------------------------------------------------------------------- :     -
P.americana_CNS00108            : ---------------------------------------------------------------------------------------------- :     -
P.multistrita_CNS00142          : ---------------------------------------------------------------------------------------------- :     -
P.pungens_CNS00055              : ---------------------------------------------------------------------------------------------- :     -
P.multiseries_CNS00149          : ---------------------------------------------------------------------------------------------- :     -

                                    2920       *      2940       *      2960       *      2980       *      3000
P.cuspidata CNS00150            : CGAGGACGACGAGGACGACGAGGACGACGACGACAAGTCATTTTAGTAAAAAATTAATAAAAAGGAAATAACCTAAACGTTT----CAACATC :  2949
P.hainanensis_CNS00090          : ------------------------------------------------------------------------------TTG----TGGAGTC :   561
P.delicatissima_CNS00130        : ------------------------------------------------------------------------------ATG----CAAAGCG :   546
P.micropora_CNS00133            : ------------------------------------------------------------------------------TTG----CGAAGCG :   552
Pseudo-nitzschia_sp._CNS00097   : ------------------------------------------------------------------------------TTG----TGAACCG :   559
P.galaxiae CNS01037             : ------------------------------------------------------------------------------TTG----CAATGTG :   562
P.americana_CNS00108            : ------------------------------------------------------------------------------CTG----CTAGAGA :   549
P.multistrita_CNS00142          : ------------------------------------------------------------------------------CTGGCATACAGTATC :   611
P.pungens_CNS00055              : ------------------------------------------------------------------------------TTGTGCACCAGAACA :   567
P.multiseries_CNS00149          : ------------------------------------------------------------------------------CTG----TGGAATC :   561
                                                                                                               Tg
```

```
                                 *      3020         *      3040         *      3060         *      3080         *      3100
P.cuspidata_CNS00150           : TTCCCATTCTTTATCCAGAATGGTAAAGAAGATTACTTTTTATTTCACTTACATTCTGTGGAGATACAGTAAAGGCATTGACCGCACCAATGGT :  3043
P.hainanensis_CNS00090         : GAGCTGCC-------------------------------------------------------------------------------------- :   569
P.delicatissima_CNS00130       : TCGTTGCC-------------------------------------------------------------------------------------- :   554
P.micropora_CNS00133           : TCA------------------------------------------------------------------------------------------- :   555
Pseudo-nitzschia_sp._CNS00097  : TCGTTGCA-------------------------------------------------------------------------------------- :   567
P.galaxiae_CNS01037            : TTCCCAGT-------------------------------------------------------------------------------------- :   570
P.americana_CNS00108           : TGATTGTT-------------------------------------------------------------------------------------- :   557
P.multistrita_CNS00142         : TTCTCTCC-------------------------------------------------------------------------------------- :   619
P.pungens_CNS00055             : ACAATGTT-------------------------------------------------------------------------------------- :   575
P.multiseries_CNS00149         : TCAAAATA-------------------------------------------------------------------------------------- :   569
                                 t

                                       *      3120         *      3140         *      3160         *      3180         *
P.cuspidata_CNS00150           : GGCGAGAGTTGCGAGAATAGGTGCTGCGAGCTTCATTTCGATGCGATTTTCACTCTTTCAAACTCAGAATATTTATTTTATGCGAAGAGTT :  3137
P.hainanensis_CNS00090         : ---------------------------------------------------------------------------------------------- :     -
P.delicatissima_CNS00130       : ---------------------------------------------------------------------------------------------- :     -
P.micropora_CNS00133           : ---------------------------------------------------------------------------------------------- :     -
Pseudo-nitzschia_sp._CNS00097  : ---------------------------------------------------------------------------------------------- :     -
P.galaxiae_CNS01037            : ---------------------------------------------------------------------------------------------- :     -
P.americana_CNS00108           : ---------------------------------------------------------------------------------------------- :     -
P.multistrita_CNS00142         : ---------------------------------------------------------------------------------------------- :     -
P.pungens_CNS00055             : ---------------------------------------------------------------------------------------------- :     -
P.multiseries_CNS00149         : ---------------------------------------------------------------------------------------------- :     -

                                3200         *      3220         *      3240         *      3260         *      3280         *
P.cuspidata_CNS00150           : GAAGGTAAGGGAATATCCGTTTAGATTCTCCGACGAGGATCCTCGCTAAGGCAGGTGAAAAATAATGCGGTAAAAGATAATTCAGAATGCTTTT :  3231
P.hainanensis_CNS00090         : ---------------------------------------------------------------------------------------------- :     -
P.delicatissima_CNS00130       : ---------------------------------------------------------------------------------------------- :     -
P.micropora_CNS00133           : ---------------------------------------------------------------------------------------------- :     -
Pseudo-nitzschia_sp._CNS00097  : ---------------------------------------------------------------------------------------------- :     -
P.galaxiae_CNS01037            : ---------------------------------------------------------------------------------------------- :     -
P.americana_CNS00108           : ---------------------------------------------------------------------------------------------- :     -
P.multistrita_CNS00142         : ---------------------------------------------------------------------------------------------- :     -
P.pungens_CNS00055             : ---------------------------------------------------------------------------------------------- :     -
P.multiseries_CNS00149         : ---------------------------------------------------------------------------------------------- :     -

                                      3300         *      3320         *      3340         *      3360         *      3380
P.cuspidata_CNS00150           : CGGGCACTTCCGACGATGCTTTTCGTATTTTTGGGCACTTCGAATGGCCACGTCAGAAGAATGCGCGGCGGCCGGATATTTTGGGAGAATATGA :  3325
P.hainanensis_CNS00090         : ---------------------------------------------------------------------------------------------- :     -
P.delicatissima_CNS00130       : ---------------------------------------------------------------------------------------------- :     -
P.micropora_CNS00133           : ---------------------------------------------------------------------------------------------- :     -
Pseudo-nitzschia_sp._CNS00097  : ---------------------------------------------------------------------------------------------- :     -
P.galaxiae_CNS01037            : ---------------------------------------------------------------------------------------------- :     -
P.americana_CNS00108           : ---------------------------------------------------------------------------------------------- :     -
P.multistrita_CNS00142         : ---------------------------------------------------------------------------------------------- :     -
P.pungens_CNS00055             : ---------------------------------------------------------------------------------------------- :     -
P.multiseries_CNS00149         : ---------------------------------------------------------------------------------------------- :     -

                                     *      3400         *      3420         *      3440         *      3460         *      34
P.cuspidata_CNS00150           : TTCGTAAGAGTGGGGCACACTGACTTTCGGGTTCACTGACAAAGGAGAAGATATTCGAGAACAAGCGACGTTGATTCAGATATGCAAACAAGAA :  3419
P.hainanensis_CNS00090         : ---------GTCAGAATTACCGATGGATATTGGCAGCAAAAAGGGAAGT--------------------------------------------- :   609
P.delicatissima_CNS00130       : -----------GGTATGCAACATTTGATCGTTTCGATAGAAAAG-------------------------------------------------- :   587
P.micropora_CNS00133           : --------------------------ATCGTTTCAATAGAGAAGTATGG------------------------------------TAACAGAAA :   587
Pseudo-nitzschia_sp._CNS00097  : -------GAGGTGTGCGTAACAGTGGATGTTTTCGATGCAACAATAAGTTCTTTTCTGTTTCAATCA------------------AAACAGAAA :   636
P.galaxiae_CNS01037            : -----------------------------GGTTCAATG-----------------------------------------------AAATAATAA :   588
P.americana_CNS00108           : -------TGTCCGTAGAGTGCGAATCTCAAATGCAATAGCAGAATATTGCAA---------------------------------AACCGATAA :   611
P.multistrita_CNS00142         : -------------------CGATCTGGTTTGTGCAGAAACCAGA-------------------------------------------------- :   644
P.pungens_CNS00055             : -----------------------GATATGCTATCGATGGCTGGT-----------------------------------------AAATGAAAG :   605
P.multiseries_CNS00149         : ---ACGAGATTGTGGATACCGGTACGATACTATCGATGGTCGATTGTGTTGATGATGGCAGTGG---------------------GAATGATGA :   639
                                                                  C  t

                                80         *      3500         *      3520         *      3540         *      3560         *
P.cuspidata_CNS00150           : TCGTGGCATATTGTACTGCCCCTATTAGTGGGACACTACGAGATCCGATCCTCCGTGCTACTGGGGTCTTTTTGAAGTCTCGAAAAAAATCAGA :  3513
P.hainanensis_CNS00090         : ---------------------------------------------------------------------------------------------- :     -
P.delicatissima_CNS00130       : ----AGCTGCTTCCC------------------------------------------------------------------------------- :   598
P.micropora_CNS00133           : TGTTGTCTGCTTTCC------------------------------------------------------------------------------- :   602
Pseudo-nitzschia_sp._CNS00097  : ACATGTTGGACTTTCTTCTTCC------------------------------------------------------------------------ :   658
P.galaxiae_CNS01037            : TAGTG----------------------------------------------------------------------------------------- :   593
P.americana_CNS00108           : TAGGTGTATGATATCCTG---------------------------------------------------------------------------- :   629
P.multistrita_CNS00142         : ---------------------------------------------------------------------------------------------- :     -
P.pungens_CNS00055             : CGGTGAGGGAATATTGT----------------------------------------------------------------------------- :   622
P.multiseries_CNS00149         : TGATGTTGTTGT---------------------------------------------------------------------------------- :   651

                                    3580         *      3600         *      3620         *      3640         *      3660
P.cuspidata_CNS00150           : CCTCGCAAATCATATTGAAATCATGCGCAGTATCTAAGTTTCGTGCTTCTTTTTGAAAGGCAATACCGATTTATTATCCAAAGAACCTCGATGG :  3607
P.hainanensis_CNS00090         : ------------------------------------TCATCTGAGTT----------------------------------------------- :   620
P.delicatissima_CNS00130       : ------------------------------------AGTTCTGAGTT----------------------------------------------- :   609
P.micropora_CNS00133           : ------------------------------------AATTTCGGCTT----------------------------------------------- :   613
Pseudo-nitzschia_sp._CNS00097  : ------------------------------------ACACTTGGCTT----------------------------------------------- :   669
P.galaxiae_CNS01037            : ------------------------------------AGTTCCGTCCT----------------------------------------------- :   604
P.americana_CNS00108           : ------------------------------------TTGTCCGA-------------------------------------------------- :   637
P.multistrita_CNS00142         : ------------------------------------TAGTACGAAAT----------------------------------------------- :   655
P.pungens_CNS00055             : ------------------------------------TTCTTTGAGCC----------------------------------------------- :   633
P.multiseries_CNS00149         : ------------------------------------TCATCCGAGTTGC--------------------------------------------- :   664
                                                                        t  G   t

                                   *      3680         *      3700         *      3720         *      3740         *      3760
P.cuspidata_CNS00150           : CTTGCGAATAACAACCTCCACGGCTTGGGAATACATAATAACCTCCCGGCTAATAACCTCCACGGCTTGGGAATACATAATAACCTCCACGGCT :  3701
P.hainanensis_CNS00090         : ---------------------------------------------------------------------------------------------- :     -
P.delicatissima_CNS00130       : ---------------------------------------------------------------------------------------------- :     -
P.micropora_CNS00133           : ---------------------------------------------------------------------------------------------- :     -
Pseudo-nitzschia_sp._CNS00097  : ---------------------------------------------------------------------------------------------- :     -
P.galaxiae_CNS01037            : ---------------------------------------------------------------------------------------------- :     -
P.americana_CNS00108           : ---------------------------------------------------------------------------------------------- :     -
P.multistrita_CNS00142         : ---------------------------------------------------------------------------------------------- :     -
P.pungens_CNS00055             : ---------------------------------------------------------------------------------------------- :     -
P.multiseries_CNS00149         : ---------------------------------------------------------------------------------------------- :     -
```

```
                                     *      3780         *      3800         *      3820         *      3840         *
P.cuspidata_CNS00150          : TCGGAACGGAATAACTGCAACCACGAAGTGCCGTGTATCCAAGCTGAACCTCAACCCGAAACGAATGGTAGCAGATGGGATACGAAAAAAGCTG : 3795
P.hainanensis_CNS00090        : ---------------------------------------------------------------------------------------------- :    -
P.delicatissima_CNS00130      : ---------------------------------------------------------------------------------------------- :    -
P.micropora_CNS00133          : ---------------------------------------------------------------------------------------------- :    -
Pseudo-nitzschia sp. CNS00097 : ---------------------------------------------------------------------------------------------- :    -
P.galaxiae_CNS01037           : ---------------------------------------------------------------------------------------------- :    -
P.americana_CNS00108          : ---------------------------------------------------------------------------------------------- :    -
P.multistrita_CNS00142        : ---------------------------------------------------------------------------------------------- :    -
P.pungens_CNS00055            : ---------------------------------------------------------------------------------------------- :    -
P.multiseries_CNS00149        : ---------------------------------------------------------------------------------------------- :    -

                                 3860         *      3880         *      3900         *      3920         *      3940
P.cuspidata_CNS00150          : ACATCAGTGCCGACCAAAACAGATGCGACAAGATTTCTAGTGACGTTTAATTCCGAAAA-AGCAGCTGTAGGGGTTCC--GAAAAACAGGTTTT : 3886
P.hainanensis_CNS00090        : ---------------------------------------------------------------------------------------------- :    -
P.delicatissima_CNS00130      : -----------------------------------------------------------------GGAAA-AAGCTCCACAAACGAG------- :  630
P.micropora_CNS00133          : -----------------------------------------------------------------GGAAAGGATCTTCTCAAACGAA------- :  635
Pseudo-nitzschia_sp._CNS00097 : -----------------------------------------CGCTGCCGAGGCCGAGAAAGATATATATGGGATCGTCGTGCAAAAC------- :  715
P.galaxiae_CNS01037           : -----------------------------------------------------------------------TGTTTTCGTCGAAAAG------- :  620
P.americana_CNS00108          : ------------------------------------------------------------GAAGATGACAGGACTATGAACTACGAG------- :  664
P.multistrita_CNS00142        : ----------------------------------------------------------------ACTGTCGAATCATGGGAAACAAC------- :  678
P.pungens_CNS00055            : --------------------ATGAGTAAGATTACTACCGTAACTGGCAACTTGCTGGCATGAAGTTGATAAAAGTTCTGAAAAGTTG------- :  700
P.multiseries_CNS00149        : ------------------------------------------------------------GAAAGTTTCAAAGTTATCGGGAAAGAA------- :  691
                                                                                          a  a

                                 *      3960         *      3980         *      4000         *      4020         *      4040
P.cuspidata_CNS00150          : CGAATCCGAATTGAAAAATAACCGGTGAATTGTATCCAAAGCAGAAGCTAGCTAGAGAAACGATGAAAGACAGCAGAATGGGATACGGTACCA : 3980
P.hainanensis_CNS00090        : ---------------------------------------------------------------------------------------------- :    -
P.delicatissima_CNS00130      : ---------------------------------------------------------------------------------------------- :    -
P.micropora_CNS00133          : ---------------------------------------------------------------------------------------------- :    -
Pseudo-nitzschia_sp._CNS00097 : ---------------------------------------------------------------------------------------------- :    -
P.galaxiae_CNS01037           : ---------------------------------------------------------------------------------------------- :    -
P.americana_CNS00108          : ---------------------------------------------------------------------------------------------- :    -
P.multistrita_CNS00142        : ---------------------------------------------------------------------------------------------- :    -
P.pungens_CNS00055            : ---------------------------------------------------------------------------------------------- :    -
P.multiseries_CNS00149        : ---------------------------------------------------------------------------------------------- :    -

                                        *      4060         *      4080         *      4100         *      4120         *
P.cuspidata_CNS00150          : GTCCGTTGGTGTTGAGAACTACCGTGAGTAAATTGTATCATCATTCGTTCTGTGGTGACGTGCATCGTGCACCTGTCAAGTTCTTCGTTTCGTC : 4074
P.hainanensis_CNS00090        : ---------------------------------------------------------------------------------------------- :    -
P.delicatissima_CNS00130      : ---------------------------------------------------------------------------------------------- :    -
P.micropora_CNS00133          : ---------------------------------------------------------------------------------------------- :    -
Pseudo-nitzschia sp. CNS00097 : ---------------------------------------------------------------------------------------------- :    -
P.galaxiae_CNS01037           : ---------------------------------------------------------------------------------------------- :    -
P.americana_CNS00108          : ---------------------------------------------------------------------------------------------- :    -
P.multistrita_CNS00142        : ---------------------------------------------------------------------------------------------- :    -
P.pungens_CNS00055            : ---------------------------------------------------------------------------------------------- :    -
P.multiseries_CNS00149        : ---------------------------------------------------------------------------------------------- :    -

                                4140         *      4160         *      4180         *      4200         *      4220         *
P.cuspidata CNS00150          : GCTTTTCGATCAGGAATATCTAACGGCCTCCCCTTGGCTTTTTTCTGTGCAGGCGATGAAGAGAAAAGCACGCTGGATGAGAGTGTACTTGAT : 4168
P.hainanensis_CNS00090        : -------------------------------------------------------------AAAAAGGGCGCGGT------------------- :  634
P.delicatissima_CNS00130      : --------------------------------------------------AAAATTAGAAAGCAAAAAATAATCT------------------- :  655
P.micropora_CNS00133          : ------------------------------------------------------TTATGGAAAAAATGGTACGAG------------------- :  656
Pseudo-nitzschia_sp._CNS00097 : -------------------------------------------------GGAGAGGAAGGAAGGAAAATCAGATA------------------- :  741
P.galaxiae CNS01037           : ---------------------------------------------------------CGAAGGAAAAGGCGATTT------------------- :  638
P.americana_CNS00108          : ------------------------------------------------------------TATGAATGGCAAGTT------------------- :  679
P.multistrita_CNS00142        : -------------------------------------------------GGAACGTACCATACGAAACGTACCGT------------------- :  704
P.pungens_CNS00055            : -----------------------------------------------------CCAACGAAGAAATCCGTATCCT------------------- :  722
P.multiseries_CNS00149        : ---------------------------------------------------------------------------------------------- :    -
                                                                                aa

                                       4240         *      4260         *      4280         *      4300         *      4320
P.cuspidata_CNS00150          : CGGTCGGGTTCCTTGAGGAACAGCGCAGTTGGAAATGTTTTGGCTTTCTTCGTTGGTGCCGCTTTCATGGCCGCGGCAATGTATGGTTCAGCAA : 4262
P.hainanensis_CNS00090        : ---------------------------------------------------------------------------------------------- :    -
P.delicatissima_CNS00130      : ---------------------------------------------------------------------------------------------- :    -
P.micropora_CNS00133          : ---------------------------------------------------------------------------------------------- :    -
Pseudo-nitzschia_sp._CNS00097 : ---------------------------------------------------------------------------------------------- :    -
P.galaxiae_CNS01037           : ---------------------------------------------------------------------------------------------- :    -
P.americana_CNS00108          : ---------------------------------------------------------------------------------------------- :    -
P.multistrita_CNS00142        : ---------------------------------------------------------------------------------------------- :    -
P.pungens_CNS00055            : ---------------------------------------------------------------------------------------------- :    -
P.multiseries_CNS00149        : ---------------------------------------------------------------------------------------------- :    -

                                     *      4340         *      4360         *      4380         *      4400         *      44
P.cuspidata_CNS00150          : ATGGCGTTACTTCCTCTAGCACGTCGGCTGTAGCTGCTATTGATTTCGCAATCAACCCAACCCGTCCAGAAGTAGATGCTCGTCAAAACTATAC : 4356
P.hainanensis_CNS00090        : ---------------------------------------------------------------------------------------------- :    -
P.delicatissima_CNS00130      : ---------------------------------------------------------------------------------------------- :    -
P.micropora_CNS00133          : ---------------------------------------------------------------------------------------------- :    -
Pseudo-nitzschia sp. CNS00097 : ---------------------------------------------------------------------------------------------- :    -
P.galaxiae_CNS01037           : ---------------------------------------------------------------------------------------------- :    -
P.americana_CNS00108          : ---------------------------------------------------------------------------------------------- :    -
P.multistrita_CNS00142        : ---------------------------------------------------------------------------------------------- :    -
P.pungens_CNS00055            : ---------------------------------------------------------------------------------------------- :    -
P.multiseries_CNS00149        : ---------------------------------------------------------------------------------------------- :    -

                                20          *      4440         *      4460         *      4480         *      4500         *
P.cuspidata CNS00150          : CGATGGAGGCTGGGGTAATAAAACCAGAAATGATGGTTGGCCAAGTTTTCATTTTGAAGTCTTTCACTTCTGCCCTCACTTGATTAACTTCATG : 4450
P.hainanensis_CNS00090        : ---------------------------------------------------------------------------------------------- :    -
P.delicatissima_CNS00130      : ---------------------------------------------------------------------------------------------- :    -
P.micropora_CNS00133          : ---------------------------------------------------------------------------------------------- :    -
Pseudo-nitzschia_sp._CNS00097 : ---------------------------------------------------------------------------------------------- :    -
P.galaxiae CNS01037           : ---------------------------------------------------------------------------------------------- :    -
P.americana_CNS00108          : ---------------------------------------------------------------------------------------------- :    -
P.multistrita_CNS00142        : ---------------------------------------------------------------------------------------------- :    -
P.pungens_CNS00055            : ---------------------------------------------------------------------------------------------- :    -
P.multiseries_CNS00149        : ---------------------------------------------------------------------------------------------- :    -
```

```
                                        4520         *       4540         *       4560         *       4580         *       4600
P.cuspidata_CNS00150          : TACGAGAACCAGGTTCATCCACAATCCAGAGTCGACGAAAATAATATCATCTATGATGCACAGCACCCTGGTGCTATTGCCGAAGATGGTTTTG : 4544
P.hainanensis_CNS00090        : ---------------------------------------------------------------------------------------------- :    -
P.delicatissima_CNS00130      : ---------------------------------------------------------------------------------------------- :    -
P.micropora_CNS00133          : ---------------------------------------------------------------------------------------------- :    -
Pseudo-nitzschia sp. CNS00097 : ---------------------------------------------------------------------------------------------- :    -
P.galaxiae_CNS01037           : ---------------------------------------------------------------------------------------------- :    -
P.americana_CNS00108          : ---------------------------------------------------------------------------------------------- :    -
P.multistrita_CNS00142        : ---------------------------------------------------------------------------------------------- :    -
P.pungens_CNS00055            : ---------------------------------------------------------------------------------------------- :    -
P.multiseries_CNS00149        : ---------------------------------------------------------------------------------------------- :    -

                                     *       4620         *       4640         *       4660         *       4680         *       4700
P.cuspidata_CNS00150          : CCGAGGTGTTGCAACAGACACTCCCCAAGTTTTACGACTATCCCGTAGGATACAGACTCAACGACCGCCCTGCAGAAATTTCTGCGTATTGGAC : 4638
P.hainanensis_CNS00090        : ---------------------------------------------------------------------------------------------- :    -
P.delicatissima_CNS00130      : ---------------------------------------------------------------------------------------------- :    -
P.micropora_CNS00133          : ---------------------------------------------------------------------------------------------- :    -
Pseudo-nitzschia sp._CNS00097 : ---------------------------------------------------------------------------------------------- :    -
P.galaxiae_CNS01037           : ---------------------------------------------------------------------------------------------- :    -
P.americana_CNS00108          : ---------------------------------------------------------------------------------------------- :    -
P.multistrita_CNS00142        : ---------------------------------------------------------------------------------------------- :    -
P.pungens_CNS00055            : ---------------------------------------------------------------------------------------------- :    -
P.multiseries_CNS00149        : ---------------------------------------------------------------------------------------------- :    -

                                            *       4720         *       4740         *       4760         *       4780         *
P.cuspidata_CNS00150          : CCTGCACATGCGAATCGCCCGCGGTAATCAAGACTCCAAGTGGCATGGCTTTGCCTCCTCTCTTCCCGAAGGCATAAGCCGAATTTACGTGAAG : 4732
P.hainanensis_CNS00090        : ---------------------------------------------------------------------------------------------- :    -
P.delicatissima_CNS00130      : ---------------------------------------------------------------------------------------------- :    -
P.micropora_CNS00133          : ---------------------------------------------------------------------------------------------- :    -
Pseudo-nitzschia sp._CNS00097 : ---------------------------------------------------------------------------------------------- :    -
P.galaxiae_CNS01037           : ---------------------------------------------------------------------------------------------- :    -
P.americana_CNS00108          : ---------------------------------------------------------------------------------------------- :    -
P.multistrita_CNS00142        : ---------------------------------------------------------------------------------------------- :    -
P.pungens_CNS00055            : ---------------------------------------------------------------------------------------------- :    -
P.multiseries_CNS00149        : ---------------------------------------------------------------------------------------------- :    -

                                   4800         *       4820         *       4840         *       4860         *       4880
P.cuspidata_CNS00150          : AAGCCCCTGCGTTGGAATGGTGATCAGGTCGCATTGTTCGGCAGCCAAACTGGCTACAACATTCTTATCTACTAATGGTTTAACTGAATAACGT : 4826
P.hainanensis_CNS00090        : ---------------------------------------------------------------------------------------------- :    -
P.delicatissima_CNS00130      : ---------------------------------------------------------------------------------------------- :    -
P.micropora_CNS00133          : ---------------------------------------------------------------------------------------------- :    -
Pseudo-nitzschia sp._CNS00097 : ---------------------------------------------------------------------------------------------- :    -
P.galaxiae_CNS01037           : ---------------------------------------------------------------------------------------------- :    -
P.americana_CNS00108          : ---------------------------------------------------------------------------------------------- :    -
P.multistrita_CNS00142        : ---------------------------------------------------------------------------------------------- :    -
P.pungens_CNS00055            : ---------------------------------------------------------------------------------------------- :    -
P.multiseries_CNS00149        : ---------------------------------------------------------------------------------------------- :    -

                                 *       4900         *       4920         *       4940         *       4960         *       4980
P.cuspidata_CNS00150          : GCTATCTACTCTAGAAGTATCTATTTGAAAATGTTGTCTTAGAATCGAAAGAAGATCCGTACGGGGTTGGAACTAATGAATGCTGTTTCGATAC : 4920
P.hainanensis_CNS00090        : ---------------------------------------------------------------------------------------------- :    -
P.delicatissima_CNS00130      : ---------------------------------------------------------------------------------------------- :    -
P.micropora_CNS00133          : ---------------------------------------------------------------------------------------------- :    -
Pseudo-nitzschia sp._CNS00097 : ---------------------------------------------------------------------------------------------- :    -
P.galaxiae_CNS01037           : ---------------------------------------------------------------------------------------------- :    -
P.americana_CNS00108          : ---------------------------------------------------------------------------------------------- :    -
P.multistrita_CNS00142        : ---------------------------------------------------------------------------------------------- :    -
P.pungens_CNS00055            : ---------------------------------------------------------------------------------------------- :    -
P.multiseries_CNS00149        : ---------------------------------------------------------------------------------------------- :    -

                                        *       5000         *       5020         *       5040         *       5060         *
P.cuspidata_CNS00150          : CCCCGTTTAAAATCGCAGGTAATCATCATCACGGAACCTAAACTTATACGCTGATCGACTGGTAGAGTACCGTACTGTCATTTTA-TAATTTGC : 5013
P.hainanensis_CNS00090        : ----------------------------------------------------------------------------GCGACTGGATTTAGTTGT :  652
P.delicatissima_CNS00130      : --------------------------------------------------------------------------TGACAACTGGA-GAAGTCGA :  674
P.micropora_CNS00133          : ---------------------------------------------------------------------------TACAAGTACG-TATTTTTT :  674
Pseudo-nitzschia sp._CNS00097 : -----------------------------------------------------------------------------TGGGTTCA-TACATAGG :  757
P.galaxiae_CNS01037           : -------------------------------------------------------------------------------AGTTCG-CAAGTACT :  652
P.americana_CNS00108          : -------------------------------------------------------------------------------CTTCAGTTGTTTTGT :  694
P.multistrita_CNS00142        : -------------------------------------------------------------------------------ATCGTA-CGTATTGT :  718
P.pungens_CNS00055            : -------------------------------------------------GTCAGTCCCCTGCAAGAATAATTCATTACCGGTACA-TAACCTTA :  766
P.multiseries_CNS00149        : ----------------------------------------------------------------------------GAAAATCGT-CACGTCCC :  708
                                                                                                 t        t

                                5080         *       5100         *       5120         *       5140         *       5160         *
P.cuspidata_CNS00150          : AAATCTTCCGCCATTGAACATCCTCCTCCTTAACGAAACGAGATGGGCAACCCAATAATTTATTTCTTCATGTCATAATTAATTCGTTTTATTA : 5107
P.hainanensis_CNS00090        : ACG---------------------------------------------------------TCGTACT--------------------------- :  662
P.delicatissima_CNS00130      : ACG--TATTGGCATCAGCGATAAAAATTGGTAGGTTAATAAT------------------CGTTACC--------------------------- :  721
P.micropora_CNS00133          : ACA---------------CACCATATTCGAGAGGGTAGGAC-------------------TATTACT--------------------------- :  707
Pseudo-nitzschia sp._CNS00097 : AAGTATATTTAAAGTACATATTATAATAGATACTTACGAGGAGATGAAAA----------TTGTACT--------------------------- :  814
P.galaxiae_CNS01037           : TCG---------------------------------------------------------TATCTCT--------------------------- :  662
P.americana_CNS00108          : ACTCGTACGAGTATGG------ATAGACAAAAGGTGCGTGAAAACAATTTCTTCCGAGTTTTTCACG--------------------------- :  755
P.multistrita_CNS00142        : ATG-----GGCTATGGGCAATCTTCATCGGAAAAAGCTAAGCGTCGGCGCG---------TGTTGCA--------------------------- :  771
P.pungens_CNS00055            : CCGATAATAAACTCTAACCCTTATTATCGACGATTATTCCAAAAA---------------TGTTTTT--------------------------- :  818
P.multiseries_CNS00149        : ACC---------------ATTTTCATTCCTAGTA--------------AACCTTAACCTTTATTACG--------------------------- :  746
                                a                                                           t tt c

                                      5180         *       5200         *       5220         *       5240         *       5260
P.cuspidata_CNS00150          : TTACTTATATACTACGGCCGTAAACGGATTAAATCATGGAGTTCATACGTTACCTTAATCTGCGTAATATCCGGTAATGAACTTGTTCTTGACA : 5201
P.hainanensis_CNS00090        : ---------------------------------------------------------------------------------------------- :    -
P.delicatissima_CNS00130      : ---------------------------------------------------------------------------------------------- :    -
P.micropora_CNS00133          : ---------------------------------------------------------------------------------------------- :    -
Pseudo-nitzschia sp._CNS00097 : ---------------------------------------------------------------------------------------------- :    -
P.galaxiae_CNS01037           : ---------------------------------------------------------------------------------------------- :    -
P.americana_CNS00108          : ---------------------------------------------------------------------------------------------- :    -
P.multistrita_CNS00142        : ---------------------------------------------------------------------------------------------- :    -
P.pungens_CNS00055            : ---------------------------------------------------------------------------------------------- :    -
P.multiseries_CNS00149        : ---------------------------------------------------------------------------------------------- :    -
```

```
                                   *      5280        *      5300        *      5320        *      5340        *       53
P.cuspidata_CNS00150         : AAGTCCCAAGTAGTTCCCTCAGGGTCATTCACGTTCAAGCCG-ATGGAATCGAAGAATGGGCACGAGTCAGTGGTCTCGTCTGGGTTAGGATTG :  5294
P.hainanensis_CNS00090       : ------------------------GCGTGTATC------------------------------------------------------------- :   671
P.delicatissima_CNS00130     : -----------------GTACCCTACGTGTACG--TAGGATA-ATGGTGCAATAATCTGGAGATGA---------------------------- :   767
P.micropora_CNS00133         : -----------------------CTCGTGTAAGATTAGGGTTAT-------------------------------------------------- :   728
Pseudo-nitzschia sp. CNS00097 : -----------------CTGAAGGGTACGTACAACTGTACTAA--------------------------------------------------- :   840
P.galaxiae_CNS01037          : --------------CCAATGTAGGACCTCTAGG------------------------------------------------------------- :   681
P.americana_CNS00108         : -----------CGACGCGCCAACGGTTTGTGGATTTGCAAAACCTAACGC-------------------------------------------- :   794
P.multistrita_CNS00142       : ------------ACCTAACGTATGACTTATGATTTGTACCTAC--------------------------------------------------- :   802
P.pungens_CNS00055           : -----------------TTGGACAACGTACA----TGGTACAAGTGATACGTAAGATTACATGTGCGTACGAGTACCCGTCTGTGAACTTC--- :   888
P.multiseries_CNS00149       : --------------AAGTTCGAAGAACTGTGCCCTTCAGATA-ATAATTCGAC----------------------------------------- :   784
                                                          t t

                               60        *      5380        *      5400        *      5420        *      5440        *
P.cuspidata_CNS00150         : ATTTGCTTGGAGGGGATAATGATCTCGGCAGGTTCGTAAAAGGTGATCAAGGAGGATCTTTCACTTCCCTCAAACAGTTCCTTGGGTGGCTTGT :  5388
P.hainanensis_CNS00090       : ---------------------------------------------------------------------------------------------- :     -
P.delicatissima_CNS00130     : ---------------------------------------------------------------------------------------------- :     -
P.micropora_CNS00133         : ---------------------------------------------------------------------------------------------- :     -
Pseudo-nitzschia_sp._CNS00097 : ---------------------------------------------------------------------------------------------- :     -
P.galaxiae_CNS01037          : ---------------------------------------------------------------------------------------------- :     -
P.americana_CNS00108         : ---------------------------------------------------------------------------------------------- :     -
P.multistrita_CNS00142       : ---------------------------------------------------------------------------------------------- :     -
P.pungens_CNS00055           : ---------------------------------------------------------------------------------------------- :     -
P.multiseries_CNS00149       : ---------------------------------------------------------------------------------------------- :     -

                                   5460        *      5480        *      5500        *      5520        *      5540
P.cuspidata_CNS00150         : CCACGCCGTGAAGGGCTGACGGAAGGTAGTTCTTGGTGAGGAATGACAAACTTTGACCAATGAAGACCAAATAGCTGTTGCGAATTCCCGGAAC :  5482
P.hainanensis_CNS00090       : ---------------------------------------------------------------------------------------------- :     -
P.delicatissima_CNS00130     : --------------------------------------------------------CAAATAGAA----------------------------- :   776
P.micropora_CNS00133         : --------------------------------------------------------TAGATAGAA----------------------------- :   737
Pseudo-nitzschia_sp._CNS00097 : --------------------------------------------------------TACGTATAATACTGGTTTTTCTACGACT---------- :   869
P.galaxiae_CNS01037          : --------------------------------------------------------TAGGTAGGT----------------------------- :   690
P.americana_CNS00108         : --------------------------------------------------------AAGGTAGCA----------------------------- :   803
P.multistrita_CNS00142       : --------------------------------------------------------CGTGTGGCA----------------------------- :   811
P.pungens_CNS00055           : --------------------------------------------------------GAAATAATG----------------------------- :   897
P.multiseries_CNS00149       : --------------------------------------------------------CTGGTACTG----------------------------- :   793
                                                                                            t

                                   *      5560        *      5580        *      5600        *      5620        *      5640
P.cuspidata_CNS00150         : GGGAACCTTTTTCCCCTCGCCGGTCTCCACGAAAAGACCCTTGAAGCCATTGTCGGCAGGAACTTGATTGGCCAAAGTGATGAAACTAGTATCC :  5576
P.hainanensis_CNS00090       : ---------------------------------------------------------------------------------------------- :     -
P.delicatissima_CNS00130     : ---------------------------------------------------------------------------------------------- :     -
P.micropora_CNS00133         : ---------------------------------------------------------------------------------------------- :     -
Pseudo-nitzschia sp. CNS00097 : ---------------------------------------------------------------------------------------------- :     -
P.galaxiae_CNS01037          : ---------------------------------------------------------------------------------------------- :     -
P.americana_CNS00108         : ---------------------------------------------------------------------------------------------- :     -
P.multistrita_CNS00142       : ---------------------------------------------------------------------------------------------- :     -
P.pungens_CNS00055           : ---------------------------------------------------------------------------------------------- :     -
P.multiseries_CNS00149       : ---------------------------------------------------------------------------------------------- :     -

                                       *      5660        *      5680        *      5700        *      5720        *
P.cuspidata CNS00150         : AAGTGTGGAACAAGACGATGAGTGAGTTCTTCCTCGTCCGATTGCTCGACCTGTGGATAGATGCGGTTGGTGAAAAACATCTTCTTCGAATTGA :  5670
P.hainanensis_CNS00090       : ---------------------------------------------------------------------------------------------- :     -
P.delicatissima_CNS00130     : ---------------------------------------------------------------------------------------------- :     -
P.micropora_CNS00133         : ---------------------------------------------------------------------------------------------- :     -
Pseudo-nitzschia_sp._CNS00097 : ---------------------------------------------------------------------------------------------- :     -
P.galaxiae CNS01037          : ---------------------------------------------------------------------------------------------- :     -
P.americana_CNS00108         : ---------------------------------------------------------------------------------------------- :     -
P.multistrita_CNS00142       : ---------------------------------------------------------------------------------------------- :     -
P.pungens_CNS00055           : ---------------------------------------------------------------------------------------------- :     -
P.multiseries_CNS00149       : ---------------------------------------------------------------------------------------------- :     -

                                 5740        *      5760        *      5780        *      5800        *      5820
P.cuspidata_CNS00150         : TAGTACTCTCGTACTCCGCTGGATCGAAATCGGGGAAACGAAGCTTGAGAGCCTTGAAAATAGTGCGAATAACCTTCTCGGCAACATTGAAGGT :  5764
P.hainanensis_CNS00090       : ---------------------------------------------------------------------------------------------- :     -
P.delicatissima_CNS00130     : ---------------------------------------------------------------------------------------------- :     -
P.micropora_CNS00133         : ---------------------------------------------------------------------------------------------- :     -
Pseudo-nitzschia_sp._CNS00097 : ---------------------------------------------------------------------------------------------- :     -
P.galaxiae_CNS01037          : ---------------------------------------------------------------------------------------------- :     -
P.americana_CNS00108         : ---------------------------------------------------------------------------------------------- :     -
P.multistrita_CNS00142       : ---------------------------------------------------------------------------------------------- :     -
P.pungens_CNS00055           : ---------------------------------------------------------------------------------------------- :     -
P.multiseries_CNS00149       : ---------------------------------------------------------------------------------------------- :     -

                                *      5840        *      5860        *      5880        *      5900        *      5920
P.cuspidata_CNS00150         : CTCCGTATGATGGGCGTCGTGGGCCTTGGTGAATTCCTCGCAAGGAGTCCTGTTCACGACCTCAGGTCCTCTCCAGAAGTACTTCACGACGTGA :  5858
P.hainanensis_CNS00090       : ---------------------------------------------------------------------------------------------- :     -
P.delicatissima_CNS00130     : ---------------------------------------------------------------------------------------------- :     -
P.micropora_CNS00133         : ---------------------------------------------------------------------------------------------- :     -
Pseudo-nitzschia sp. CNS00097 : ---------------------------------------------------------------------------------------------- :     -
P.galaxiae_CNS01037          : ---------------------------------------------------------------------------------------------- :     -
P.americana_CNS00108         : ---------------------------------------------------------------------------------------------- :     -
P.multistrita_CNS00142       : ---------------------------------------------------------------------------------------------- :     -
P.pungens_CNS00055           : ---------------------------------------------------------------------------------------------- :     -
P.multiseries_CNS00149       : ---------------------------------------------------------------------------------------------- :     -

                                      *      5940        *      5960        *      5980        *      6000        *
P.cuspidata CNS00150         : TCCTTTTGTGGCTTGCCCAAATTTCCTCCGGCAATGGAGGAGCCTTGGAAGGGAACGTATCCGCCATTTTGGAAGTGGGTGTCGGTGTAATACT :  5952
P.hainanensis_CNS00090       : ---------------------------------------------------------------------------------------------- :     -
P.delicatissima_CNS00130     : ---------------------------------------------------------------------------------------------- :     -
P.micropora_CNS00133         : ---------------------------------------------------------------------------------------------- :     -
Pseudo-nitzschia_sp._CNS00097 : ---------------------------------------------------------------------------------------------- :     -
P.galaxiae CNS01037          : ---------------------------------------------------------------------------------------------- :     -
P.americana_CNS00108         : ---------------------------------------------------------------------------------------------- :     -
P.multistrita_CNS00142       : ---------------------------------------------------------------------------------------------- :     -
P.pungens_CNS00055           : ---------------------------------------------------------------------------------------------- :     -
P.multiseries_CNS00149       : ---------------------------------------------------------------------------------------------- :     -
```

```
                                6020         *      6040         *      6060         *      6080         *      6100         *
P.cuspidata_CNS00150          : TCATCCTCTCTTCCTCGGTAGTGGTGGAAATAAACTCTCTGAATGCGTTGTAAACGTTGATGGAGACCTCT----------TCAGGCACTCCAT :  6036
P.hainanensis_CNS00090        : ---------------------------------------------------------------------------------GCTGATAGTCTGT :   684
P.delicatissima_CNS00130      : --------------------------------------ACTAAAAAATGTCCCCCGATAATGCCTTCACGA----------TCTGATAGCCC-- :   821
P.micropora_CNS00133          : -------------------------------------ACTAAATAATGTCTAT------ATGCAGTCCTAA----------TCCAAAAAACTAC :   778
Pseudo-nitzschia sp. CNS00097 : -------------------------------------ACAAAGTTCACTCGATTCATGTATGTATCCATTG----------TGTGGAAGCAAAC :   916
P.galaxiae_CNS01037           : -----------------------------------------------ACCTAGACGTAGTCGGAGGTCACAACCTAAA---ACTGGTAC----- :   729
P.americana_CNS00108          : ---------------------------------------TGCATGTCAAGTACTCGTAGTGACATATCAAG----------TACGACTACCGTA :   848
P.multistrita_CNS00142        : ----------------------------------CTCCCTTACATACCTTTACTCGCGCAGGGAGACATCG----------CTCGAAAG----- :   856
P.pungens_CNS00055            : ------------------------------------------ATACGCTCGATGAATTGATGACATTCTCTGCTCTAT---TACAATAGCCC-T :   945
P.multiseries_CNS00149        : ---------------------------------------TACTTCCTACGTACCGGTAGTTGTAATTTTTGTTTACTCTGTACAAAAGCCCAT :   848
                                                                                   a         g                         a

                                      6120         *      6140         *      6160         *      6180         *      6200
P.cuspidata_CNS00150          : GGTTCACAATGTAGAAAAGTCCCGATTTGCGGATATCAACCAAAAACTGTTCCAACTCCTCTTTCGTTTCGACCTTGCTGATGTCAATCGCAGG :  6130
P.hainanensis_CNS00090        : CGTC------------------------------------------------------------------------------------------ :   688
P.delicatissima_CNS00130      : --TACATGATACCT-------------------------------------------------------------------------------- :   833
P.micropora_CNS00133          : AGTATGCCGTGCCT-------------------------------------------------------------------------------- :   792
Pseudo-nitzschia_sp._CNS00097 : ---------------------------------------------------------------------------------------------- :     -
P.galaxiae_CNS01037           : ---------------------------------------------------------------------------------------------- :     -
P.americana_CNS00108          : GTGTACGAGTACGATACAAACGAGT--------------------------------------------------------------------- :   873
P.multistrita_CNS00142        : ---------------------------------------------------------------------------------------------- :     -
P.pungens_CNS00055            : TTTCAATGGTACTGTACGAATTCCACC------------------------------------------------------------------- :   972
P.multiseries_CNS00149        : GTTCGATGGTACGAAC------------------------------------------------------------------------------ :   864

                                     *      6220         *      6240         *      6260         *      6280         *      63
P.cuspidata_CNS00150          : GATCTCCTCGGGGAGAACATCTCTCGGGAACCACTTCAACTTGTCGGCTTCGAGCGGAGGGAATCGTCTCTCCAAAGTTTGCCCTTGTTGACC :  6224
P.hainanensis_CNS00090        : -------------------------------------------------------------------------------------------GCC :   691
P.delicatissima_CNS00130      : -------------------------------------------------------------------------------------------ACG :   836
P.micropora_CNS00133          : -------------------------------------------------------------------------------------------ACA :   795
Pseudo-nitzschia_sp._CNS00097 : -------------------------------------------------------------------------------------------ACA :   919
P.galaxiae_CNS01037           : -------------------------------------------------------------------------------------------ACG :   732
P.americana_CNS00108          : -------------------------------------------------------------------------------------------ACG :   876
P.multistrita_CNS00142        : -------------------------------------------------------------------------------------------ACT :   859
P.pungens_CNS00055            : -------------------------------------------------------------------------------------------GCA :   975
P.multiseries_CNS00149        : -------------------------------------------------------------------------------------------ACT :   867
                                                                                                           aC

                                00         *      6320         *      6340         *      6360         *      6380         *
P.cuspidata_CNS00150          : TGGATGTTA-----------------TCCTCGGTAGGGGTCAAATC--GGTGGTTTCGTTGTTTACAACTTGCGCAGTCATCTTTACGGAGTAA :  6299
P.hainanensis_CNS00090        : CTGTTGCT---------------------TTTGCAGTCATG-------GATTTTTCCGTTG--------------------------------- :   724
P.delicatissima_CNS00130      : AAAGAGGTA--------------------TGTGCAGTAGCC-------GACTTTTCCGTTG--------------------------------- :   870
P.micropora_CNS00133          : CGATGGGTA-----------------TGTTGTGCGGAAGTC-------GACTTTTCCGTTG--------------------------------- :   832
Pseudo-nitzschia_sp._CNS00097 : AGGGTGGTT---------------------------------------GACTTTTCCGTTG--------------------------------- :   941
P.galaxiae_CNS01037           : TTAACGTT------------------TTTCTACCGCACGAAATTTC--GTCTTTACCGTTG--------------------------------- :   773
P.americana_CNS00108          : TGATACGT---------------------GCATCGGTCGTTGTTTC--ATTTTTTTCATTC--------------------------------- :   914
P.multistrita_CNS00142        : TCATCGGTAACAAA---------------TTTCCGAGCACG-------AGCATTACCAATC--------------------------------- :   898
P.pungens_CNS00055            : TTGTTGGTAAGTACTAAGTACTCTAGAGTTTAACGGGAAGCGATACGAGACGTAATCGGTG--------------------------------- :  1036
P.multiseries_CNS00149        : TCGACGGT-------------TGGCAAATCTGAAAGAAAGCGAAAC--AGTATGTGCATTG--------------------------------- :   913
                                     g T                                            tt  C tTg

                                   6400          *     6420          *     6440          *     6460          *     6480
P.cuspidata_CNS00150          : AAGGGAACGTTAGTTTAAAATCAGCAATGCGAGTAGAGTCTGTTGACGGTTGCGTCAATTTCAATGCGAAAGGTTATGCGAGAAAACGTTCCGA :  6393
P.hainanensis_CNS00090        : ---------------------------------------------------------------------------------------------- :     -
P.delicatissima_CNS00130      : ---------------------------------------------------------------------------------------------- :     -
P.micropora_CNS00133          : ---------------------------------------------------------------------------------------------- :     -
Pseudo-nitzschia_sp._CNS00097 : ---------------------------------------------------------------------------------------------- :     -
P.galaxiae_CNS01037           : ---------------------------------------------------------------------------------------------- :     -
P.americana_CNS00108          : ---------------------------------------------------------------------------------------------- :     -
P.multistrita_CNS00142        : ---------------------------------------------------------------------------------------------- :     -
P.pungens_CNS00055            : ---------------------------------------------------------------------------------------------- :     -
P.multiseries_CNS00149        : ---------------------------------------------------------------------------------------------- :     -

                                   *      6500         *      6520         *      6540         *      6560         *      6580
P.cuspidata_CNS00150          : AAGCTCCCAAAACTTGCACAAGAATTCCAAGTACGAGGTATGACTCTTACCCTTAATGAAATCTTTTTATTTCTTACCGGCATTACCGGCATT :  6487
P.hainanensis_CNS00090        : ---------------------------------------------------------------------------------------------- :     -
P.delicatissima_CNS00130      : ---------------------------------------------------------------------------------------------- :     -
P.micropora_CNS00133          : ---------------------------------------------------------------------------------------------- :     -
Pseudo-nitzschia_sp._CNS00097 : ---------------------------------------------------------------------------------------------- :     -
P.galaxiae_CNS01037           : ---------------------------------------------------------------------------------------------- :     -
P.americana_CNS00108          : ---------------------------------------------------------------------------------------------- :     -
P.multistrita_CNS00142        : ---------------------------------------------------------------------------------------------- :     -
P.pungens_CNS00055            : ---------------------------------------------------------------------------------------------- :     -
P.multiseries_CNS00149        : ---------------------------------------------------------------------------------------------- :     -

                                         *      6600         *      6620         *      6640         *      6660         *
P.cuspidata_CNS00150          : TGATCCTACTGCCAAAAGATTCATTCAAGATTACGTGAATAGCCTCGCGCTATAAAATCTTCGTTTGAACCGAGGTGGAAACTGACGTGTTATC :  6581
P.hainanensis_CNS00090        : ---------------------------------------------------------------------------------------------- :     -
P.delicatissima_CNS00130      : ---------------------------------------------------------------------------------------------- :     -
P.micropora_CNS00133          : ---------------------------------------------------------------------------------------------- :     -
Pseudo-nitzschia_sp._CNS00097 : ---------------------------------------------------------------------------------------------- :     -
P.galaxiae_CNS01037           : ---------------------------------------------------------------------------------------------- :     -
P.americana_CNS00108          : ---------------------------------------------------------------------------------------------- :     -
P.multistrita_CNS00142        : ---------------------------------------------------------------------------------------------- :     -
P.pungens_CNS00055            : ---------------------------------------------------------------------------------------------- :     -
P.multiseries_CNS00149        : ---------------------------------------------------------------------------------------------- :     -

                                 6680          *     6700          *     6720          *     6740          *     6760
P.cuspidata_CNS00150          : TCGAGACACGTCCGCTAAAAATTTCGCACGTAGTGCTCCCCTAGCACTGGTACTGGAACATACGTATGTCATGTTTTCGTACGGCGAACAATCT :  6675
P.hainanensis_CNS00090        : ---------------------------------------------TCCGATTT----------------------------------------- :   732
P.delicatissima_CNS00130      : -------------------------------------------AAAATGATTC----------------------------------------- :   880
P.micropora_CNS00133          : -------------------------------------------AAAATGATTC----------------------------------------- :   842
Pseudo-nitzschia_sp._CNS00097 : ------------------------------------------GAGACGGAAAC----------------------------------------- :   952
P.galaxiae_CNS01037           : -------------------------------------------AGACGAAAGT----------------------------------------- :   783
P.americana_CNS00108          : ------------------------------GCTTTCTTTCCACACGACGATGC----------------------------------------- :   937
P.multistrita_CNS00142        : ------------------------AGTACCACACTCTAATCACTTACCGGTAC----------------------------------------- :   927
P.pungens_CNS00055            : ----------------------------CAGTAGGTGGAAGACATACCGGTAC----------------------------------------- :  1061
P.multiseries_CNS00149        : ----------CCTCTGGATATTGTAAAGTTATTATATTTTTCACTACCGGTAC----------------------------------------- :   956
                                                                             a  g t c
```

```
                                    *      6780         *      6800         *      6820         *      6840         *      6860
P.cuspidata_CNS00150          : TCCTATGAAATCCGCGGAATTCCCCGACTGCGCCCAAAGTTGTACTTGCAGTAGAGAGAACAATTGAAAATGCGGACTAATTCTCTTCCTCAAG :  6769
P.hainanensis_CNS00090        : ---------------------------------------------------------------------------------------------- :     -
P.delicatissima_CNS00130      : ---------------------------------------------------------------------------------------------- :     -
P.micropora_CNS00133          : ---------------------------------------------------------------------------------------------- :     -
Pseudo-nitzschia sp. CNS00097 : ---------------------------------------------------------------------------------------------- :     -
P.galaxiae_CNS01037           : ---------------------------------------------------------------------------------------------- :     -
P.americana_CNS00108          : ---------------------------------------------------------------------------------------------- :     -
P.multistrita_CNS00142        : ---------------------------------------------------------------------------------------------- :     -
P.pungens_CNS00055            : ---------------------------------------------------------------------------------------------- :     -
P.multiseries_CNS00149        : ---------------------------------------------------------------------------------------------- :     -

                                          *      6880         *      6900         *      6920         *      6940         *
P.cuspidata_CNS00150          : GTGAAAAGATCGTTGCAGTGATATCGCCCATTTGAGAAGAGCTCGTACGAACGCGTGCCGTGCGCAGCACGAGGGAGGTTCGGGAGCGAGAACC :  6863
P.hainanensis_CNS00090        : ---------------------------------------------------------------------------------------------- :     -
P.delicatissima_CNS00130      : ---------------------------------------------------------------------------------------------- :     -
P.micropora_CNS00133          : ---------------------------------------------------------------------------------------------- :     -
Pseudo-nitzschia_sp._CNS00097 : ---------------------------------------------------------------------------------------------- :     -
P.galaxiae_CNS01037           : ---------------------------------------------------------------------------------------------- :     -
P.americana CNS00108          : ---------------------------------------------------------------------------------------------- :     -
P.multistrita_CNS00142        : ---------------------------------------------------------------------------------------------- :     -
P.pungens_CNS00055            : ---------------------------------------------------------------------------------------------- :     -
P.multiseries_CNS00149        : ---------------------------------------------------------------------------------------------- :     -

                                6960         *      6980         *      7000         *      7020         *      7040         *
P.cuspidata_CNS00150          : CGACGAAGAGACGAACGATATGCATAGGATGCGAAGATCTCGTCGTGAATTCGACGTTTTTGTCTTATTTACACTCCATTTCAAGAGCAGCTT :  6957
P.hainanensis_CNS00090        : ---------------------------------------------------------------------------------------------- :     -
P.delicatissima_CNS00130      : ---------------------------------------------------------------------------------------------- :     -
P.micropora_CNS00133          : ---------------------------------------------------------------------------------------------- :     -
Pseudo-nitzschia_sp._CNS00097 : ---------------------------------------------------------------------------------------------- :     -
P.galaxiae_CNS01037           : ---------------------------------------------------------------------------------------------- :     -
P.americana_CNS00108          : ---------------------------------------------------------------------------------------------- :     -
P.multistrita_CNS00142        : ---------------------------------------------------------------------------------------------- :     -
P.pungens_CNS00055            : ---------------------------------------------------------------------------------------------- :     -
P.multiseries_CNS00149        : ---------------------------------------------------------------------------------------------- :     -

                                      7060         *      7080         *      7100         *      7120         *      7140
P.cuspidata_CNS00150          : CCACTCCTGCTCACTCGTAATCAAGATGACGCTTGTAACATCTATTCTTTTTGCCGGGCTGGCCGTAGCCGGAAAGGTTGGATCGAACTATTAC :  7051
P.hainanensis_CNS00090        : ---------------------------------------------------------------------------------------------- :     -
P.delicatissima_CNS00130      : ---------------------------------------------------------------------------------------------- :     -
P.micropora_CNS00133          : ---------------------------------------------------------------------------------------------- :     -
Pseudo-nitzschia sp. CNS00097 : ---------------------------------------------------------------------------------------------- :     -
P.galaxiae_CNS01037           : ---------------------------------------------------------------------------------------------- :     -
P.americana_CNS00108          : ---------------------------------------------------------------------------------------------- :     -
P.multistrita_CNS00142        : ---------------------------------------------------------------------------------------------- :     -
P.pungens_CNS00055            : ---------------------------------------------------------------------------------------------- :     -
P.multiseries_CNS00149        : ---------------------------------------------------------------------------------------------- :     -

                                    *      7160         *      7180         *      7200         *      7220         *      72
P.cuspidata CNS00150          : GACTTCATATCTTCCCCTCGGGATGACGAAGTCGGGTTTGTTGAAACCATCAACATTGGAATGGCAATTGCCTACGCCACTCTTGGTTTTGTCG :  7145
P.hainanensis_CNS00090        : ---------------------------------------------------------------------------------------------- :     -
P.delicatissima_CNS00130      : ---------------------------------------------------------------------------------------------- :     -
P.micropora_CNS00133          : ---------------------------------------------------------------------------------------------- :     -
Pseudo-nitzschia_sp._CNS00097 : ---------------------------------------------------------------------------------------------- :     -
P.galaxiae CNS01037           : ---------------------------------------------------------------------------------------------- :     -
P.americana_CNS00108          : ---------------------------------------------------------------------------------------------- :     -
P.multistrita_CNS00142        : ---------------------------------------------------------------------------------------------- :     -
P.pungens_CNS00055            : ---------------------------------------------------------------------------------------------- :     -
P.multiseries_CNS00149        : ---------------------------------------------------------------------------------------------- :     -

                                40         *      7260         *      7280         *      7300         *      7320         *
P.cuspidata_CNS00150          : TGTCCGCTATTTTTAGCTTCGCATGGAGCTACTATGAGTTTCGGTTTTCTCCCCTGCGAGATGCCCCTGGTTTTGGACCGAAATCGT-TTGTCT :  7238
P.hainanensis_CNS00090        : ---------------------------------------------------------------------------------------------- :     -
P.delicatissima_CNS00130      : ---------------------------------------------------------------------------------------------A :   881
P.micropora_CNS00133          : ---------------------------------------------------------------------------------------------A :   843
Pseudo-nitzschia_sp._CNS00097 : ---------------------------------------------------------------------------------------------A :   953
P.galaxiae_CNS01037           : ---------------------------------------------------------------------------------------------- :     -
P.americana_CNS00108          : -------------------------------------------------------------------------------AGTACAGTCTAGCTG :   952
P.multistrita_CNS00142        : ----------------------------------------------------------------------------------AATTATCAGTAA :   939
P.pungens_CNS00055            : ----------------------------------------GACTACCTCGTACCATAGCCTTTTCTCGTTTCCGTCCCCTAGGTAAGTCGGTGG :  1115
P.multiseries_CNS00149        : ---------------------------------GTACTTGTCCGTACAAAAAATAACAATCTTTCTCTACATTCACCGTGATATTAT-TAGTGT :  1016

                                     7340         *      7360         *      7380         *      7400         *      7420
P.cuspidata_CNS00150          : ACGGTATGTTCTACTTGCATATACGAACTCTTTTCGTCGTCGAAAATAAATTAATTTTTCTTTCGTCGTCGAAAATCAATATTTTTTTTCGTTT :  7332
P.hainanensis_CNS00090        : ---GTGAA-------------------------------------------------------------------------------------- :   737
P.delicatissima_CNS00130      : ATGATGGA-------------------------------------------------------------------------------------- :   889
P.micropora_CNS00133          : ATGATGGA-------------------------------------------------------------------------------------- :   851
Pseudo-nitzschia_sp._CNS00097 : TTGATGAA-------------------------------------------------------------------------------------- :   961
P.galaxiae_CNS01037           : ---------------------------------------------------------------------------------------------- :     -
P.americana_CNS00108          : ACAGTCTG-------------------------------------------------------------------------------------- :   960
P.multistrita_CNS00142        : ACGAGGAA-------------------------------------------------------------------------------------- :   947
P.pungens_CNS00055            : ATAGTACG-------------------------------------------------------------------------------------- :  1123
P.multiseries_CNS00149        : ACGGTACA-------------------------------------------------------------------------------------- :  1024
                                    t

                                      *      7440         *      7460         *      7480         *      7500         *      7520
P.cuspidata CNS00150          : GTCATCTAAAATGAATTCTTTTTCATTCTCCAATTTTCTAGGTATGTTCTTTGAATTTCTAGAAGCGCCTTTCATGGAACCTCCAATCAAAGCT :  7426
P.hainanensis_CNS00090        : ---------------------------------------------------------------------------------------------- :     -
P.delicatissima_CNS00130      : ---------------------------------------------------------------------------------------------- :     -
P.micropora_CNS00133          : ---------------------------------------------------------------------------------------------- :     -
Pseudo-nitzschia_sp._CNS00097 : ---------------------------------------------------------------------------------------------- :     -
P.galaxiae CNS01037           : ---------------------------------------------------------------------------------------------- :     -
P.americana_CNS00108          : ---------------------------------------------------------------------------------------------- :     -
P.multistrita_CNS00142        : ---------------------------------------------------------------------------------------------- :     -
P.pungens_CNS00055            : ---------------------------------------------------------------------------------------------- :     -
P.multiseries_CNS00149        : ---------------------------------------------------------------------------------------------- :     -
```

```
                                        *       7540         *       7560         *       7580         *       7600         *
P.cuspidata_CNS00150          : CTCAAGAAGCTACGGGAAGGAGGCAAGGATGTCCCGTTTTTGGCCTACACGACTTTGTTTGGAAGTCAAAGACTCATGGTGTTGGACTGCGACT :  7520
P.hainanensis_CNS00090        : ---------------------------------------------------------------------------------------------- :     -
P.delicatissima_CNS00130      : ---------------------------------------------------------------------------------------------- :     -
P.micropora_CNS00133          : ---------------------------------------------------------------------------------------------- :     -
Pseudo-nitzschia_sp._CNS00097 : ---------------------------------------------------------------------------------------------- :     -
P.galaxiae_CNS01037           : ---------------------------------------------------------------------------------------------- :     -
P.americana_CNS00108          : ---------------------------------------------------------------------------------------------- :     -
P.multistrita_CNS00142        : ---------------------------------------------------------------------------------------------- :     -
P.pungens_CNS00055            : ---------------------------------------------------------------------------------------------- :     -
P.multiseries_CNS00149        : ---------------------------------------------------------------------------------------------- :     -

                                   7620         *       7640         *       7660         *       7680         *       7700
P.cuspidata_CNS00150          : TGGTCAAGCACATCTGGACATCGCCATCTGGCAGAGACCCCATGCGATACCCCAAGCACTACATTTATTTCAGGGAGGTAGTTGGAGACGGTCT :  7614
P.hainanensis_CNS00090        : ---------------------------------------------------------------------------------------------- :     -
P.delicatissima_CNS00130      : ---------------------------------------------------------------------------------------------- :     -
P.micropora_CNS00133          : ---------------------------------------------------------------------------------------------- :     -
Pseudo-nitzschia_sp._CNS00097 : ---------------------------------------------------------------------------------------------- :     -
P.galaxiae_CNS01037           : ---------------------------------------------------------------------------------------------- :     -
P.americana_CNS00108          : ---------------------------------------------------------------------------------------------- :     -
P.multistrita_CNS00142        : ---------------------------------------------------------------------------------------------- :     -
P.pungens_CNS00055            : ---------------------------------------------------------------------------------------------- :     -
P.multiseries_CNS00149        : ---------------------------------------------------------------------------------------------- :     -

                                  *       7720         *       7740         *       7760         *       7780         *       7800
P.cuspidata_CNS00150          : GGTGGTAGTTGAGGGGACAGAATGGAGTCGTCATCGTCGCATTGTCCAGCCGGCATTCCAATCTGTTTTTCTCAAAGACGCTATCAGTGCCGTT :  7708
P.hainanensis_CNS00090        : ---------------------------------------------------------------------------------------------- :     -
P.delicatissima_CNS00130      : ------------------------------CAATCGT--------------------------------------------------------- :   896
P.micropora_CNS00133          : ------------------------------CAATCGT--------------------------------------------------------- :   858
Pseudo-nitzschia_sp._CNS00097 : --------------------------------ATCGTTTTGTCGT------------------------------------------------- :   974
P.galaxiae_CNS01037           : ---------------------------------------------------------------------------------------------- :     -
P.americana_CNS00108          : ---------------------------------------------------------------------------------------------- :     -
P.multistrita_CNS00142        : ---------------------------------------------------------------------------------------------- :     -
P.pungens_CNS00055            : ------------------------------TAACCGC--------------------------------------------------------- :  1130
P.multiseries_CNS00149        : ---------------------------TCGTAATCTT--------------------------------------------------------- :  1034

                                        *       7820         *       7840         *       7860         *       7880         *
P.cuspidata_CNS00150          : GTGCCTCCCTTGGCGGAGAATCTCGTGAAAGTGTGGAAGAAGGCTGCCGGAGCGACGATCAACATGAACGCCCACCTTTCGCTTGTAACTCTTG :  7802
P.hainanensis_CNS00090        : ---------------------------------------------------------------------------------------------- :     -
P.delicatissima_CNS00130      : ---------------------------------------------------------------------------------------------- :     -
P.micropora_CNS00133          : ---------------------------------------------------------------------------------------------- :     -
Pseudo-nitzschia_sp._CNS00097 : ---------------------------------------------------------------------------------------------- :     -
P.galaxiae_CNS01037           : ---------------------------------------------------------------------------------------------- :     -
P.americana_CNS00108          : ---------------------------------------------------------------------------------------------- :     -
P.multistrita_CNS00142        : ---------------------------------------------------------------------------------------------- :     -
P.pungens_CNS00055            : ---------------------------------------------------------------------------------------------- :     -
P.multiseries_CNS00149        : ---------------------------------------------------------------------------------------------- :     -

                                7900         *       7920         *       7940         *       7960         *       7980         *
P.cuspidata_CNS00150          : ACGTGATTGGAAAAGTTGCCTTTTCGCACGAATTCAACGCATCCAACTTAATCAACGAGTGGGGCGAAAGTCCTGATAAGGAATTGGGAGAAGT :  7896
P.hainanensis_CNS00090        : ---------------------------------------------------------------------------------------------- :     -
P.delicatissima_CNS00130      : ---------------------------------------------------------------------------------------------- :     -
P.micropora_CNS00133          : ---------------------------------------------------------------------------------------------- :     -
Pseudo-nitzschia_sp._CNS00097 : ---------------------------------------------------------------------------------------------- :     -
P.galaxiae_CNS01037           : ---------------------------------------------------------------------------------------------- :     -
P.americana_CNS00108          : ---------------------------------------------------------------------------------------------- :     -
P.multistrita_CNS00142        : ---------------------------------------------------------------------------------------------- :     -
P.pungens_CNS00055            : ---------------------------------------------------------------------------------------------- :     -
P.multiseries_CNS00149        : ---------------------------------------------------------------------------------------------- :     -

                                      8000         *       8020         *       8040         *       8060         *       8080
P.cuspidata_CNS00150          : GGATGATCCGTTGCTTACATCCATCGGATCTGCCCTTTCCTCTGGCTATCTCAAGTTATTGCTAACTGTATTGAAGCTTCCGTGGCTCGAGAAG :  7990
P.hainanensis_CNS00090        : ----------------------------------------------------------------------------------TTTGTGAAAAGT :   749
P.delicatissima_CNS00130      : ----------------------------------------------------------------------------------TGTCAGAAATAA :   908
P.micropora_CNS00133          : ----------------------------------------------------------------------------------TGTCAGAAAACA :   870
Pseudo-nitzschia_sp._CNS00097 : ----------------------------------------------------------------------------------TGTCTCAACAAA :   986
P.galaxiae_CNS01037           : ----------------------------------------------------------------------------------TGTCTCGAAAAA :   795
P.americana_CNS00108          : -------------------------------------------------------------------------TGGGACCAGTGACAACAAAGC :   981
P.multistrita_CNS00142        : -----------------------------------------------------------------------CGGAGATCTGGTGCCTTAGAAAC :   970
P.pungens_CNS00055            : ------------------------------------------------------------------------------------TCGAGGGGAA :  1140
P.multiseries_CNS00149        : ---------------------------------------------------------------------------------ACGTCTT------ :  1041
                                                                                                  tg c

                                      *       8100         *       8120         *       8140         *       8160         *       81
P.cuspidata_CNS00150          : ------TACGTTAGTCCCTCGTTCAGGAATTCTCGCAACTTGCTCAACAAAGCCGCCGATGACATCGTACAAAATGCCAAGAACATCGATGATC :  8078
P.hainanensis_CNS00090        : ATGACATAC---ATTCCTTCATTCAAC------------------------------------------------------------------- :   773
P.delicatissima_CNS00130      : ------TAC--------TCGGTTTGGG---------AAGTAGTT-------------------------------------------------- :   929
P.micropora_CNS00133          : ACAGGCTAC--------TTGGTTTTGAATTTGCATCAAGTAGT--------------------------------------------------- :   905
Pseudo-nitzschia_sp._CNS00097 : AA----TAC--------TCCATACGAA------------------------------------------------------------------- :  1001
P.galaxiae_CNS01037           : ------TAC------TTTTTGTT----------------------------------------------------------------------- :   806
P.americana_CNS00108          : CAAACGAAC------------------------------------------------------------------------------------- :   990
P.multistrita_CNS00142        : ---------------------------------------------------------------------------------------------- :     -
P.pungens_CNS00055            : GGAAGTAAC----CTTACCGGTACGAAAGCTCCAAAAAC------------------------------------------------------- :  1175
P.multiseries_CNS00149        : ------TATGGTAATTACCCGCTAGTA------------------------------------------------------------------- :  1062
                                       ac

                                80         *       8200         *       8220         *       8240         *       8260         *
P.cuspidata_CNS00150          : CGAAGAGACGAAGTGTATTGAACCTCATGATGGAAGCGAAGGACGACGAATCTAGCAAGGCTCGCAATAAGTTGACGGATAGCGAGCTTCGCGA :  8172
P.hainanensis_CNS00090        : ---------------------------------------------------------------------------------------------- :     -
P.delicatissima_CNS00130      : ---------------------------------------------------------------------------------------------- :     -
P.micropora_CNS00133          : ---------------------------------------------------------------------------------------------- :     -
Pseudo-nitzschia_sp._CNS00097 : ---------------------------------------------------------------------------------------------- :     -
P.galaxiae_CNS01037           : ---------------------------------------------------------------------------------------------- :     -
P.americana_CNS00108          : ---------------------------------------------------------------------------------------------- :     -
P.multistrita_CNS00142        : ---------------------------------------------------------------------------------------------- :     -
P.pungens_CNS00055            : ---------------------------------------------------------------------------------------------- :     -
P.multiseries_CNS00149        : ---------------------------------------------------------------------------------------------- :     -
```

```
                                   8280         *      8300         *      8320         *      8340         *      8360
P.cuspidata_CNS00150          : TGAAGTCAAAACATTCCTCGTTGCTGGCCACGAAACGACCTCGACTTGGTGTCACTGGGCTTTCTATATGCTCGCGACCCGCCCTGATCTCCAA :  8266
P.hainanensis_CNS00090        : ---------------------------------------------------------------------------------------------- :     -
P.delicatissima_CNS00130      : ---------------------------------------------------------------------------------------------- :     -
P.micropora_CNS00133          : ---------------------------------------------------------------------------------------------- :     -
Pseudo-nitzschia_sp._CNS00097 : ---------------------------------------------------------------------------------------------- :     -
P.galaxiae_CNS01037           : ---------------------------------------------------------------------------------------------- :     -
P.americana_CNS00108          : ---------------------------------------------------------------------------------------------- :     -
P.multistrita_CNS00142        : ---------------------------------------------------------------------------------------------- :     -
P.pungens_CNS00055            : ---------------------------------------------------------------------------------------------- :     -
P.multiseries_CNS00149        : ---------------------------------------------------------------------------------------------- :     -

                                  *      8380         *      8400         *      8420         *      8440         *      8460
P.cuspidata_CNS00150          : GAAAAGGTATTTGCCGACGTCACCAAGTATGCACCTGGCAAGGATACTATCACTCTGGAACAAGCCGATCAAATGGAATATCTTTGGGCATTTA :  8360
P.hainanensis_CNS00090        : ---------------------------------------------------------------------------------------------- :     -
P.delicatissima_CNS00130      : -------------------------------------------------------------------------------------GTAAATCAT :   938
P.micropora_CNS00133          : ----------------------------------------------------------------------------------------ATTGGA :   911
Pseudo-nitzschia_sp._CNS00097 : -------------------------------------------------------------------------------------GCAAAGCAT :  1010
P.galaxiae_CNS01037           : ---------------------------------------------------------------------------------------------G :   807
P.americana_CNS00108          : ---------------------------------------------------------------------------------------------- :     -
P.multistrita_CNS00142        : ----------------------------------------------------------------------------------------AACCAG :   976
P.pungens_CNS00055            : ----------------------------------------------------------------------------------AAAAAAAATCTG :  1187
P.multiseries_CNS00149        : --------------------------------------------------------------------------------------CGAGTATG :  1070

                                        *      8480         *      8500         *      8520         *      8540         *
P.cuspidata_CNS00150          : TGACGGAAACCCTTCGTTTGTACCCGCCTATTGGCCTGATCTCGCGCGTCACGTACAAGGAAGAAAAATTCAAGGGATACACCATTCCAAAGGG :  8454
P.hainanensis_CNS00090        : ---------------------------------------------------------------------------------------------- :     -
P.delicatissima_CNS00130      : CGACG----------------------------------------------------------------------------------------- :   943
P.micropora_CNS00133          : TAACA----------------------------------------------------------------------------------------- :   916
Pseudo-nitzschia_sp._CNS00097 : TGATG----------------------------------------------------------------------------------------- :  1015
P.galaxiae_CNS01037           : TGACG----------------------------------------------------------------------------------------- :   812
P.americana_CNS00108          : ---CG----------------------------------------------------------------------------------------- :   992
P.multistrita_CNS00142        : AGACGGAGTCGAGT-------------------------------------------------------------------------------- :   990
P.pungens_CNS00055            : GAGCT----------------------------------------------------------------------------------------- :  1192
P.multiseries_CNS00149        : GGGAG----------------------------------------------------------------------------------------- :  1075

                                 8560         *      8580         *      8600         *      8620         *      8640
P.cuspidata_CNS00150          : TACGAAT--------TTAAGGATCCCAATCCACTTGATTCACAGACATCCCGATCATTGGAAGGACCCCGAAGAGTTCCAACCCGAGCGCTGGT :  8540
P.hainanensis_CNS00090        : -ATACAT--------TCCTTCATTCA-------------------------------------------------------------------- :   790
P.delicatissima_CNS00130      : -ATTGAT--------TGATGGATTC--------------------------------------------------------------------- :   959
P.micropora_CNS00133          : -ACCGAT--------TGATGAATTT--------------------------------------------------------------------- :   932
Pseudo-nitzschia_sp._CNS00097 : -ATCTAT--------TGATGGATTC--------------------------------------------------------------------- :  1031
P.galaxiae_CNS01037           : -ACGGAT--------TGATAGATTC--------------------------------------------------------------------- :   828
P.americana_CNS00108          : -ATCGAT--------TGATTGATTG--------------------------------------------------------------------- :  1008
P.multistrita_CNS00142        : -AACAAG--------TAACGCATCA--------------------------------------------------------------------- :  1006
P.pungens_CNS00055            : -GTACATGCTGTGCCTTACAGTTCC------------------------------------------------------------------AGT :  1219
P.multiseries_CNS00149        : -GTAACT--------TACCGTACCG--------------------------------------------------------------------- :  1091
                                 a   at        T a   at

                                *      8660         *      8680         *      8700         *      8720         *      8740
P.cuspidata_CNS00150          : TCGACAAGGAAGAATGCAGCAAGAGACACAAGTTCGCATACGTCCCCTTTTCTGCAGGTGGAAGAAACTGCATTGGCCAGCGTTTTGCTTCGAT :  8634
P.hainanensis_CNS00090        : ---------------------------------------------------------------------------------------------- :     -
P.delicatissima_CNS00130      : -----------GGATCTA---------------------------------------------------------------------------- :   966
P.micropora_CNS00133          : -----------GGAT------------------------------------------------------------------------------- :   936
Pseudo-nitzschia_sp._CNS00097 : -----------GGATGCA---------------------------------------------------------------------------- :  1038
P.galaxiae_CNS01037           : -----------GGATGCA---------------------------------------------------------------------------- :   835
P.americana_CNS00108          : --------GATGACTATAATAAT----------------------------------------------------------------------- :  1023
P.multistrita_CNS00142        : --------AAGGAAAACA---------------------------------------------------------------------------- :  1016
P.pungens_CNS00055            : ACGATAGTGGTAGGTGCG---------------------------------------------------------------------------- :  1237
P.multiseries_CNS00149        : -------TACAGGA-------------------------------------------------------------------------------- :  1098
                                           g

                                      *      8760         *      8780         *      8800         *      8820         *
P.cuspidata_CNS00150          : GGAGGCCAAGATCATCATCGCAAACGTTGCTAGGAATTTCAAAATTCATTTGGACGATTCCATGAAAGGAAAAGAAATTTCTTTTACCAGTTTC :  8728
P.hainanensis_CNS00090        : -------------------------------------------------------------------------------------ATCATCCTC :   799
P.delicatissima_CNS00130      : -------------------------------------------------------------------------------------ATCACC--- :   972
P.micropora_CNS00133          : -------------------------------------------------------------------------------------CTCATAGCC :   945
Pseudo-nitzschia_sp._CNS00097 : -------------------------------------------------------------------------------------ACCATC--- :  1044
P.galaxiae_CNS01037           : -------------------------------------------------------------------------------------ATCAGTGTC :   844
P.americana_CNS00108          : -------------------------------------------------------------------------------------ATCATATCC :  1032
P.multistrita_CNS00142        : -------------------------------------------------------------------------------------ACCAAATTC :  1025
P.pungens_CNS00055            : -------------------------------------------------------------------------------------ACTACCGGT :  1246
P.multiseries_CNS00149        : -------------------------------------------------------------------------------------ACCACCA-- :  1105
                                                                                                     a cA

                                8840        *      8860         *      8880         *      8900         *      8920         *
P.cuspidata_CNS00150          : ATTTCTCTCAAGTGCAAGCCTGATATTCTAATCCGTGTAGAACCACGAGACTAGGAATTTGAGGAAAGCCCATCGATGATACGAGGAACCCGCA :  8822
P.hainanensis_CNS00090        : GCT------------------------------------------------------------------------------------------- :   802
P.delicatissima_CNS00130      : ---------------------------------------------------------------------------------------------- :     -
P.micropora_CNS00133          : ---------------------------------------------------------------------------------------------- :     -
Pseudo-nitzschia_sp._CNS00097 : ---------------------------------------------------------------------------------------------- :     -
P.galaxiae_CNS01037           : AT-------------------------------------------------------------------------------------------- :   846
P.americana_CNS00108          : GACTTGCAATA----------------------------------------------------------------------------------- :  1043
P.multistrita_CNS00142        : A--------------------------------------------------------------------------------------------- :  1026
P.pungens_CNS00055            : AAGTCACTCGTACGAAGT---------------------------------------------------------------------------- :  1264
P.multiseries_CNS00149        : ---------------------------------------------------------------------------------------------- :     -

                                     8940         *      8960         *      8980         *      9000         *      9020
P.cuspidata_CNS00150          : GAATTTTTTTTAATCATTTACATAAACATAATTTTAGTTCATACTACTACATGAAATCCAGTGCACAAGATACGAAGATCGTTTCGCAACCCCC :  8916
P.hainanensis_CNS00090        : ---------------------------------------------------------------------------------------------- :     -
P.delicatissima_CNS00130      : ---------------------------------------------------------------------------------------------- :     -
P.micropora_CNS00133          : ---------------------------------------------------------------------------------------------- :     -
Pseudo-nitzschia_sp._CNS00097 : ---------------------------------------------------------------------------------------------- :     -
P.galaxiae_CNS01037           : ---------------------------------------------------------------------------------------------- :     -
P.americana_CNS00108          : ---------------------------------------------------------------------------------------------- :     -
P.multistrita_CNS00142        : ---------------------------------------------------------------------------------------------- :     -
P.pungens_CNS00055            : ---------------------------------------------------------------------------------------------- :     -
P.multiseries_CNS00149        : ---------------------------------------------------------------------------------------------- :     -
```

```
                                    *     9040        *     9060        *     9080        *     9100        *     91
P.cuspidata_CNS00150          : CGTTGTAAATCTGTAGTTTAAACCAATTCCCAAATGAATTTTAAAAACCGGAGTGTCGAAGATGGCTATTCTCTAAAAAAACATTCTATTTTTT : 9010
P.hainanensis_CNS00090        : ---------------------------------------------------------------------------------------------- :    -
P.delicatissima_CNS00130      : ---------------------------------------------------------------------------------------------- :    -
P.micropora_CNS00133          : ---------------------------------------------------------------------------------------------- :    -
Pseudo-nitzschia_sp._CNS00097 : ---------------------------------------------------------------------------------------------- :    -
P.galaxiae_CNS01037           : ---------------------------------------------------------------------------------------------- :    -
P.americana_CNS00108          : ---------------------------------------------------------------------------------------------- :    -
P.multistrita_CNS00142        : ---------------------------------------------------------------------------------------------- :    -
P.pungens_CNS00055            : ---------------------------------------------------------------------------------------------- :    -
P.multiseries_CNS00149        : ---------------------------------------------------------------------------------------------- :    -

                                20          *     9140        *     9160        *     9180        *     9200        *
P.cuspidata_CNS00150          : CCTGTGAATTTTTAGTATCATTCTATTTTTTCCTTTGAATTTTTAGGATCATTCTATTTGGTTGAATTTTGGTTTGCCAACGCCGTC-AAAGC : 9103
P.hainanensis_CNS00090        : -----------------------------------------------------------------------------------TGATC-AAATA :  812
P.delicatissima_CNS00130      : -----------------------------------------------------------------------------------TCATC-CAAGA :  982
P.micropora_CNS00133          : -----------------------------------------------------------------------------------CCTTCTCAAAA :  956
Pseudo-nitzschia_sp._CNS00097 : -----------------------------------------------------------------------------------CCATC-GGAAA : 1054
P.galaxiae_CNS01037           : -----------------------------------------------------------------------------------CCGTC-AAAAC :  856
P.americana_CNS00108          : -----------------------------------------------------------------------------------CCATTCCAAG- : 1053
P.multistrita_CNS00142        : -----------------------------------------------------------------------------------------AAAGG : 1031
P.pungens_CNS00055            : -----------------------------------------------------------------------------------TCATA-AAAAA : 1274
P.multiseries_CNS00149        : -----------------------------------------------------------------------------------CCACTAAGAAA : 1116
                                                                                                    c t  aA

                                    9220        *     9240        *     9260        *     9280        *     9300
P.cuspidata_CNS00150          : CACCAAAAGCGCTACCTACAACAAATTGAAATTTCCTTCCTAACGCACTGCACCATTGCATTATTCGATATTGGTAGTCGAGTCAAGGTTGCGG : 9197
P.hainanensis_CNS00090        : T----ATTGCG------------------------------------------------------AGGCTTCGATGATCAA------------- :  835
P.delicatissima_CNS00130      : G----AAAACG------------------------------------------------------AGTTGTCTATGATCAA------------- : 1005
P.micropora_CNS00133          : G----AAAACT------------------------------------------------------AATTGTCTATGACCAA------------- :  979
Pseudo-nitzschia_sp._CNS00097 : C----AAAACG------------------------------------------------------AAGTGTCTATGATCAG------------- : 1077
P.galaxiae_CNS01037           : C----AAAGCA-------------------------------------------------------GGTGTTTGTGATCAA------------- :  878
P.americana_CNS00108          : -----GAATCA--------------------------------------------------------GCTTTGGTGTGCCT------------- : 1073
P.multistrita_CNS00142        : C----AGCGCA----------------------------------------------------------GCTTGCGATT--------------- : 1048
P.pungens_CNS00055            : TAATAATATCG----------------------------------------------------------GTCTGTCGTGCG------------- : 1297
P.multiseries_CNS00149        : CTCCATAAGTA-------------------------------------------------------TCGGTCTGTCGTTCA------------- : 1142
                                     a a c                                                            t   t  t

                                   *     9320        *     9340        *     9360        *     9380        *     9400
P.cuspidata_CNS00150          : GTTGCGGGCTCGAAAAAGAGCCATCCGATCTCGCAATTTGTCGATACTCTATCGAAGTTCCCCGTGGTGGAATTTGGAAAGACAACCATATTTC : 9291
P.hainanensis_CNS00090        : ----AGAGGCT--------------------------------------------------------------------------------TTT :  845
P.delicatissima_CNS00130      : ----CGCTACC--------------------------------------------------------------------------------TGT : 1015
P.micropora_CNS00133          : ----CGCTAAC--------------------------------------------------------------------------------TAT :  989
Pseudo-nitzschia_sp._CNS00097 : ----CACATCC--------------------------------------------------------------------------------TGC : 1087
P.galaxiae_CNS01037           : ----CGCGCTC--------------------------------------------------------------------------------TTT :  888
P.americana_CNS00108          : ----CGACGTTATTA----------------------------------------------------------------------------TTC : 1087
P.multistrita_CNS00142        : ----CTGCATCATAAGT--------------------------------------------------------------------------TGT : 1064
P.pungens_CNS00055            : ----GCATTTCAAAAAAAAAAAAAAAAGTAAGGTTACTCGAAATAATT-------------------------------------------TTT : 1344
P.multiseries_CNS00149        : ----CAACATC-----------------------ACCTCAAAATTATT-------------------------------------------TTC : 1166
                                    c     c                                                                                T

                                      *     9420        *     9440        *     9460        *     9480        *
P.cuspidata_CNS00150          : CATCGACAGAGAATCCATATGCTATCATCGATACTGCCGGGCTTGTCGCAGTGTCGAATGAAGGTTTCCACAACCACCGAAACACTTTCGGATC : 9385
P.hainanensis_CNS00090        : CTTCAG---------------------------------------------------------------------------------------- :  851
P.delicatissima_CNS00130      : TGACAA---------------------------------------------------------------------------------------- : 1021
P.micropora_CNS00133          : TGACAA---------------------------------------------------------------------------------------- :  995
Pseudo-nitzschia_sp._CNS00097 : CCCCAG---------------------------------------------------------------------------------------- : 1093
P.galaxiae_CNS01037           : CGTCAA---------------------------------------------------------------------------------------- :  894
P.americana_CNS00108          : TTTCAC---------------------------------------------------------------------------------------- : 1093
P.multistrita_CNS00142        : TCTCCG---------------------------------------------------------------------------------------- : 1070
P.pungens_CNS00055            : CGTCATCACCGATCA------------------------------------------------------------------------------- : 1359
P.multiseries_CNS00149        : CGTCATCA-------------------------------------------------------------------------------------- : 1174
                                   Ca

                                  9500        *     9520        *     9540        *     9560        *     9580
P.cuspidata_CNS00150          : GCGGCATTGTGGAAACTATTCCGAAAGCTGCTATTCCGGTAGCAGCCCTGGCTTCCGCGAAAGGGTGGCCACCAATGGATGGATGGCAATCGAT : 9479
P.hainanensis_CNS00090        : -------------------ACTAGATACTA----------------------------------------------------------------- :  862
P.delicatissima_CNS00130      : -------------------GCCAGACACTA----------------------------------------------------------------- : 1032
P.micropora_CNS00133          : -------------------GCCAGACACTG----------------------------------------------------------------- : 1006
Pseudo-nitzschia_sp._CNS00097 : -------------------TCCAGACACTG----------------------------------------------------------------- : 1104
P.galaxiae_CNS01037           : -------------------TCCAGAAGTTG----------------------------------------------------------------- :  905
P.americana_CNS00108          : -------------------TGCAACTACTCGTAAT------------------------------------------------------------ : 1109
P.multistrita_CNS00142        : -------------------AACAAACACCA----------------------------------------------------------------- : 1081
P.pungens_CNS00055            : -------------------ACTATCAACGG----------------------------------------------------------------- : 1370
P.multiseries_CNS00149        : -------------------ATGATCAACGG----------------------------------------------------------------- : 1185
                                                      a   ac

                                *     9600        *     9620        *     9640        *     9660        *     9680
P.cuspidata_CNS00150          : TTCGGTGCCGGCACAAGGGTTTGCGACTCCACGAGGAGTGTCGTCCGAGCAGATCGACCGGAAGTGGCATTTTTGTAGCGGTTGCCACAATCGA : 9573
P.hainanensis_CNS00090        : ---------------------------------------------------------------------------------------------- :    -
P.delicatissima_CNS00130      : ---------------------------------------------------------------------------------------------- :    -
P.micropora_CNS00133          : ---------------------------------------------------------------------------------------------- :    -
Pseudo-nitzschia_sp._CNS00097 : ---------------------------------------------------------------------------------------------- :    -
P.galaxiae_CNS01037           : ---------------------------------------------------------------------------------------------- :    -
P.americana_CNS00108          : ---------------------------------------------------------------------------------------------- :    -
P.multistrita_CNS00142        : ---------------------------------------------------------------------------------------------- :    -
P.pungens_CNS00055            : ---------------------------------------------------------------------------------------------- :    -
P.multiseries_CNS00149        : ---------------------------------------------------------------------------------------------- :    -

                                      *     9700        *     9720        *     9740        *     9760        *
P.cuspidata_CNS00150          : CGCCAGTCCCATTGGGATGGATCCTTGGAGTGGCGTATCCATTTTGGTCACGATTTCACTTGTAGAATGCTCGGTGGGCGACGAATTCCAAGCC : 9667
P.hainanensis_CNS00090        : ---------------------------------------------------------------------------------------------- :    -
P.delicatissima_CNS00130      : ---------------------------------------------------------------------------------------------- :    -
P.micropora_CNS00133          : ---------------------------------------------------------------------------------------------- :    -
Pseudo-nitzschia_sp._CNS00097 : ---------------------------------------------------------------------------------------------- :    -
P.galaxiae_CNS01037           : ---------------------------------------------------------------------------------------------- :    -
P.americana_CNS00108          : ---------------------------------------------------------------------------------------------- :    -
P.multistrita_CNS00142        : ---------------------------------------------------------------------------------------------- :    -
P.pungens_CNS00055            : ---------------------------------------------------------------------------------------------- :    -
P.multiseries_CNS00149        : ---------------------------------------------------------------------------------------------- :    -
```

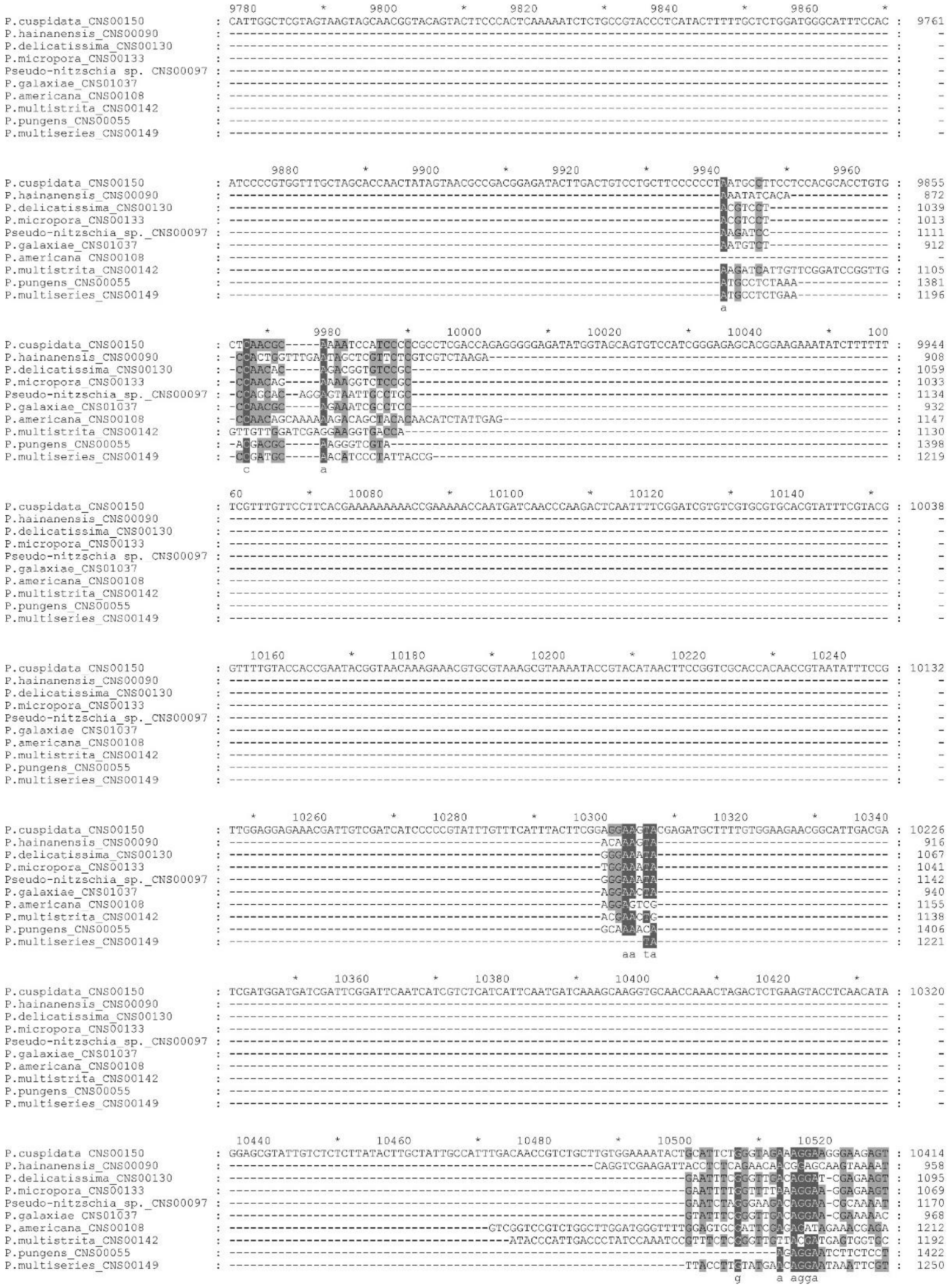

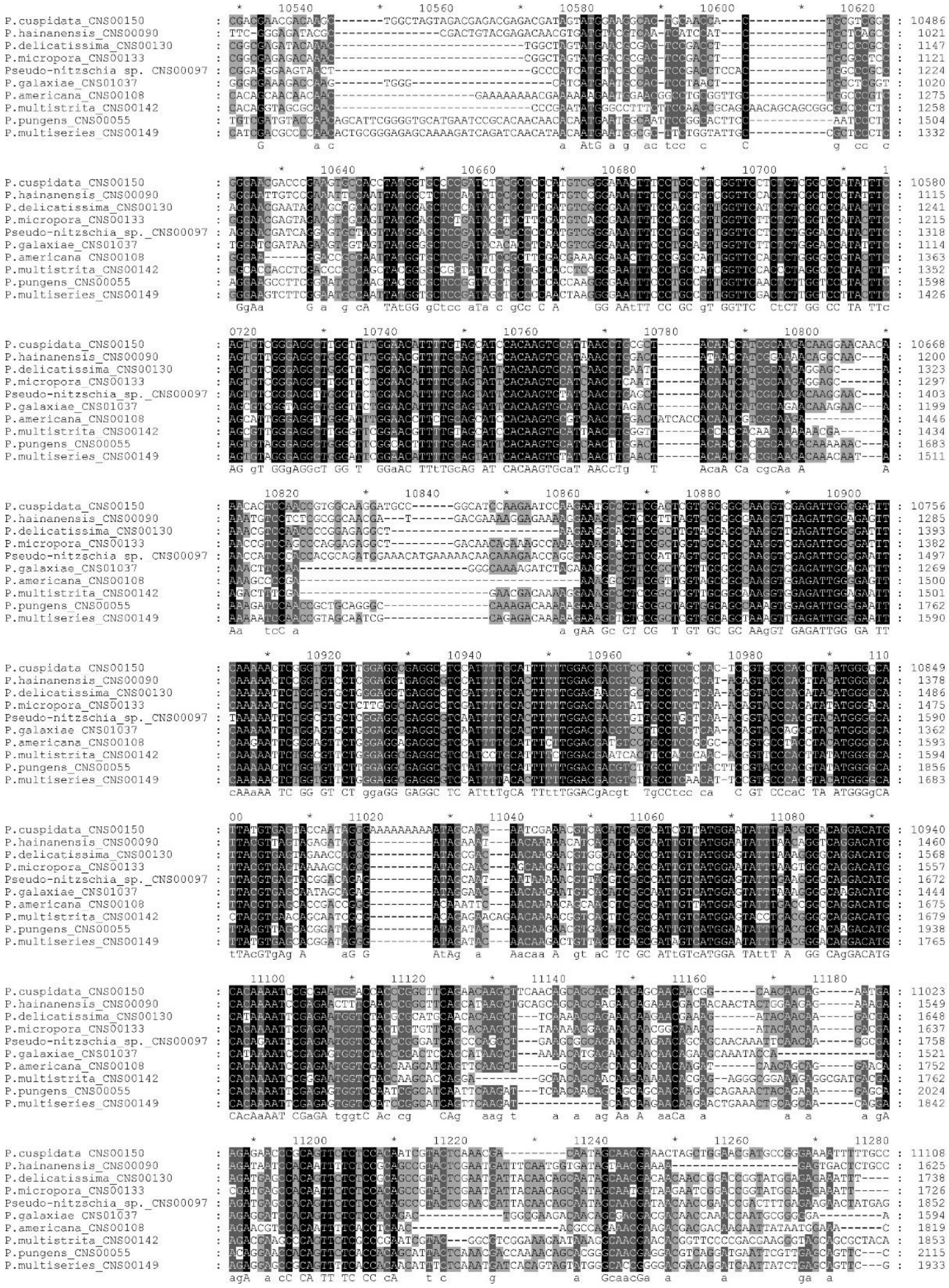

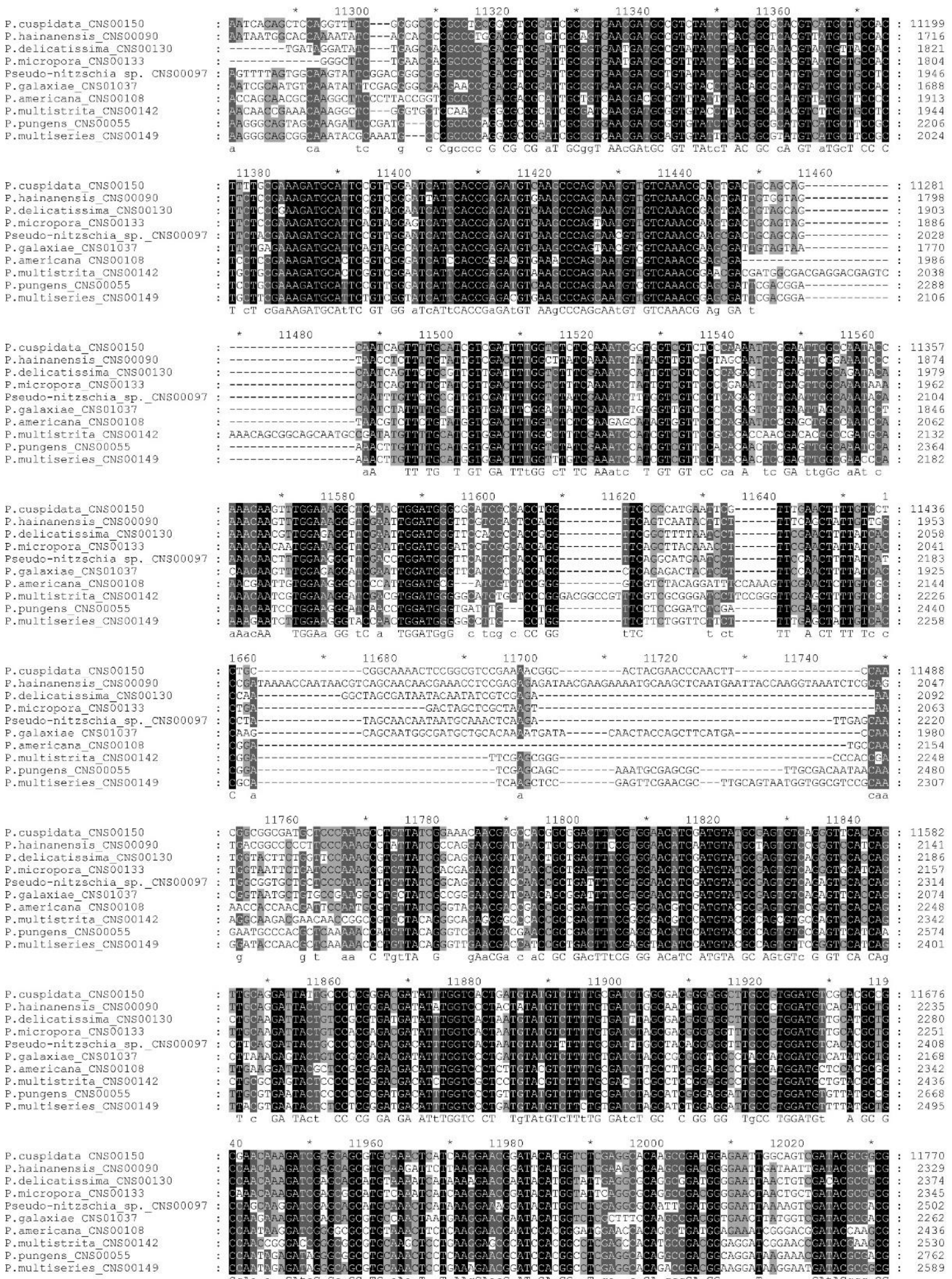

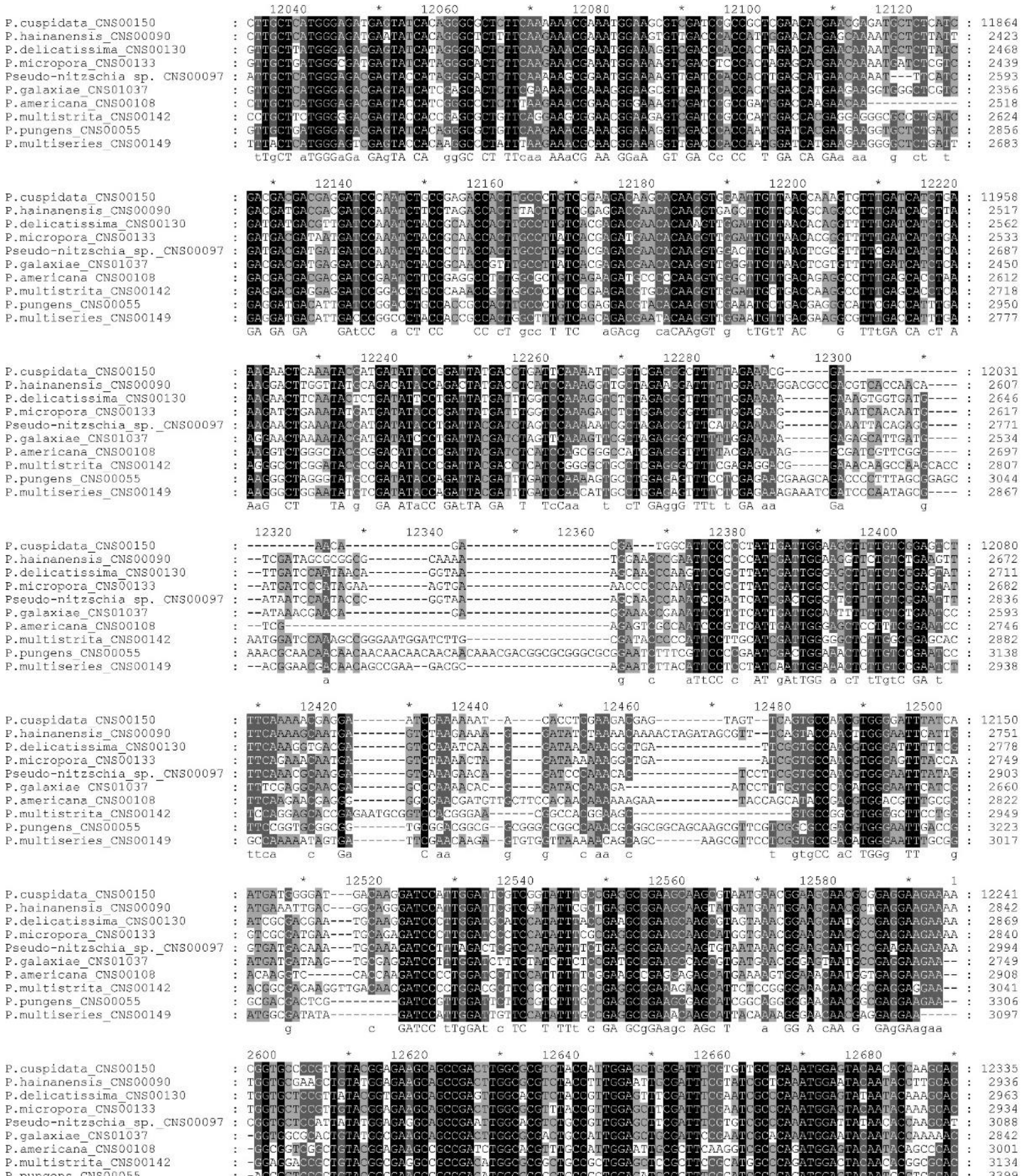
P.cuspidata_CNS00150
P.hainanensis_CNS00090
P.delicatissima_CNS00130
P.micropora_CNS00133
Pseudo-nitzschia_sp._CNS00097
P.galaxiae_CNS01037
P.americana_CNS00108
P.multistrita_CNS00142
P.pungens_CNS00055
P.multiseries_CNS00149

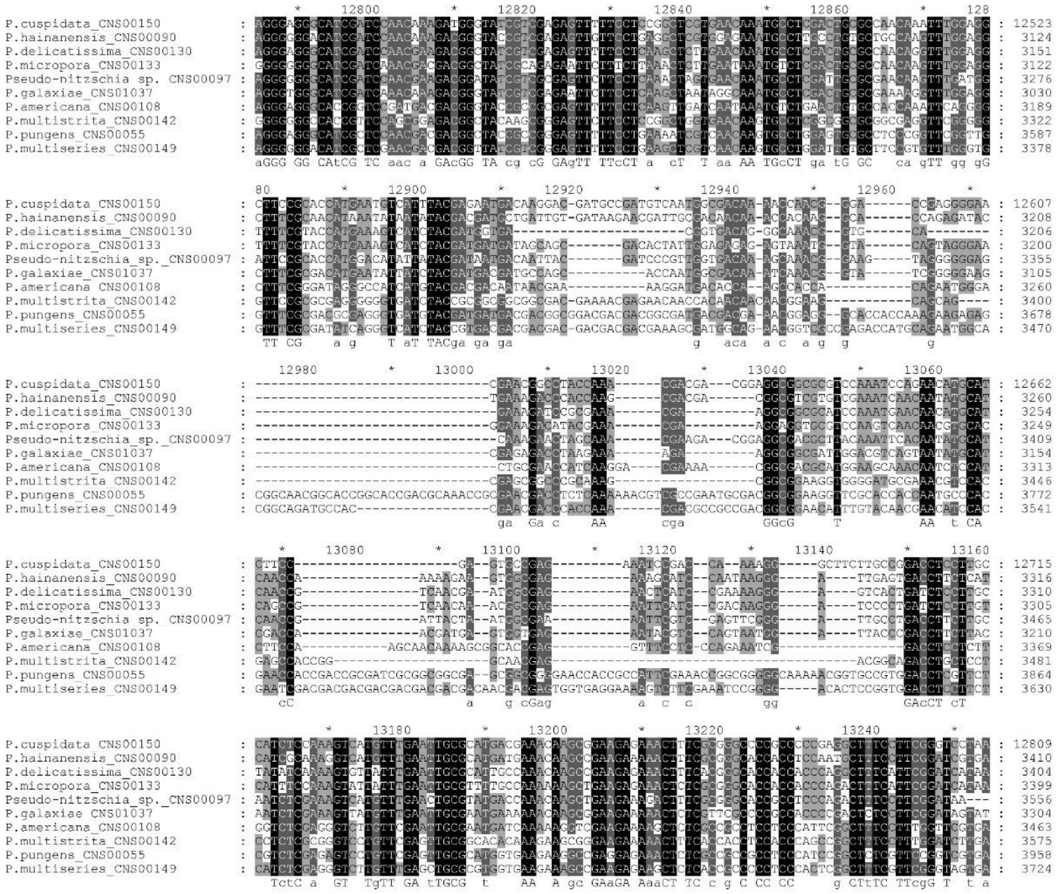

Figure S5. Comparative analysis of the original integration sites of the *dab* gene clusters in *Pseudo-nitzschia* specie

Table S1. PacBio DNA sequencing statistics

Species	Total_bases (Gb)	Total_reads	Average_Length (bp)	Max_Length (bp)	N50_Length (bp)
P. delicatissima	15.06	1,128,855	13,343	199,071	17,742
P. pungens	20.40	1,751,066	11,652	193,645	15,452
P. multiseries	31.87	2,333,202	13,659	177,725	20,759

Table S2. Genome survey analysis based on Illumina DNA sequencing results for chromosome-level genomes of three *Pseudo-nitzschia* species

Species	K-mer	Genome size (bp)	Repeat (%)	Heterozygous Ratio (%)
P. delicatissima	21	35,368,204	11.00	1.37
P. pungens	21	78,025,286	42.10	0.82
P. multiseries	21	26,694,703	71.00	0.87

Table S3. Illumina DNA sequencing results for Hi-C analysis

Species	Read Length (bp)	Total_reads	Raw Data (Gb)
P. delicatissima	150	53,992,150	16.20
P. pungens	150	128,592,643	38.58
P. multiseries	150	90,369,371	27.11

Table S4. List of chromosome lengths of assembled genomes

P. delicatissima		***P. pungens***		***P. multiseries***	
Chr	Length(bp)	Chr	Length(bp)	Chr	Length(bp)
PdeChr1	6,176,542	*Ppu_Chr1*	9,664,134	*PmuChr1*	39,424,003
PdeChr2	3,380,540	*Ppu_Chr2*	8,793,300	*PmuChr2*	31,079,748
PdeChr3	3,335,727	*Ppu_Chr3*	7,933,526	*PmuChr3*	29,406,015
PdeChr4	3,187,709	*Ppu_Chr4*	6,579,687	*PmuChr4*	28,641,012
PdeChr5	2,913,840	*Ppu_Chr5*	6,147,877	*PmuChr5*	27,622,669
PdeChr6	2,904,346	*Ppu_Chr6*	5,873,255	*PmuChr6*	18,650,752
PdeChr7	2,882,420	*Ppu_Chr7*	5,225,000	*PmuChr7*	18,465,182
PdeChr8	2,662,131	*Ppu_Chr8*	4,438,191	*PmuChr8*	17,104,344
PdeChr9	2,176,710	*Ppu_Chr9*	4,023,694	*PmuChr9*	14,554,712
PdeChr10	2,022,392	*Ppu_Chr1*	3,081,471	*PmuChr10*	14,529,497
PdeChr11	1,222,578	*Ppu_Chr11*	2,514,578	*PmuChr11*	11,523,998
		Ppu_Chr12	2,261,203		

Table S5. BUSCO analysis results

	P. delicatissima		*P. pungens*		*P. multiseries*	
**Type**	**Number**	**Percentage (%)**	**Number**	**Percentage (%)**	**Number**	**Percentage (%)**
**Assembly**						
Complete BUSCOs (C)	224	73.93%	244	80.53%	247	81.52%
Complete and single-copy BUSCOs (S)	217	71.62%	238	78.55%	236	77.89%
Complete and duplicated BUSCOs (D)	7	2.31%	6	1.98%	11	3.63%
Fragmented BUSCOs (F)	22	7.26%	9	2.97%	10	3.30%
Missing BUSCOs (M)	57	18.81%	50	16.50%	46	15.18%
Total BUSCO groups searched	303	100.00%	303	100.00%	303	100.00%
**Annotation**						
Complete BUSCOs (C)	215	70.96%	250	82.51%	254	83.83%
Complete and single-copy BUSCOs (S)	205	67.66%	242	79.87%	241	79.54%
Complete and duplicated BUSCOs (D)	10	3.30%	8	2.64%	13	4.29%
Fragmented BUSCOs (F)	28	9.24%	16	5.28%	18	5.94%
Missing BUSCOs (M)	29	9.57%	37	12.21%	31	10.23%
Total BUSCO groups searched	303	100.00%	303	100.00%	303	100.00%

Table S6. Summary of gene prediction results

	P. delicatissima	*P. pungens*	*P. multiseries*
Number	14375	15472	18648
Average mRNA length (bp)	1472	1935	1824
Average CDS length (bp)	1129	1522	1481

Average exon per gene	1.80	1.99	1.87
Average exon length (bp)	626	766	792
Average intron length (bp)	158	368	306

Table S7. Functional annotation of predicted genes

	P. delicatissima		***P. pungens***		***P. multiseries***	
	Number	Percentage	Number	Percentage	Number	Percentage
Total	14,375	-	15,472	-	18,648	-
Nr	12,895	89.70%	14,067	90.92%	15,612	83.72%
Swissprot	6044	42.05%	6,126	39.59%	6,573	35.25%
KEGG	5585	38.85%	5,743	37.12%	6,117	32.80%
KOG	5610	39.03%	5,680	36.71%	6,148	32.97%
TrEMBL	13,390	93.15%	7,742	50.04%	16,342	87.63%
Interpro	9349	65.04%	9,631	62.25%	11,064	59.33%
GO	5653	39.33%	5,872	37.95%	6,319	33.89%
Overall	13,488	93.83%	14,148	91.44%	16,502	88.49%

Table S8. Sampling information, DA analysis and *dab* gene cluster of *Pseudo-nitzschia* strains in this study.

Strain ID	Species	DA analysis	*dab* gene cluster	Sampling site	Sampling date	Longitude (°E)	Latitude (°N)
CNS00108	*P. americana*	-	Not found	Jiaozhou Bay, China	2019.10	120.52	36.21
CNS00138	*P. americana*	Not detected	Not found	Jiaozhou Bay, China	2019.10	120.34	36.16
CNS00151	*P. americana*	-	Not found	Jiaozhou Bay, China	2019.10	120.43	36.35
CNS01029	*P. brasiliana*	Not detected	Not found	Nan'ao Island, China	2021.6	117.11	23.48
CNS00150	*P. cuspidata*	-	Yes	East China Sea, China	2019.9	122.57	28.47
**CNS00130**	*P. delicatissima*	Not detected	Not found	Yellow Sea, China	2019.4	122.83	34.01
CNS00135	*P. delicatissima*	-	Not found	Jiaozhou Bay, China	2019.10	120.61	36.05
CNS00969	*P. galaxiae*	-	Not found	South China Sea, China	2021.5	112.50	20.00
CNS01037	*P. galaxiae*	-	Not found	South China Sea, China	2021.5	115.00	20.00
CNS01103	*P. galaxiae*	Not detected	Not found	South China Sea, China	2021.5	118.00	13.00
CNS00090	*P. hainanensis*	-	Not found	West Pacific	2019.6	140.15	10.75
CNS00133	*P. micropora*	Not detected	Not found	Jiaozhou Bay, China	2019.7	120.25	36.16
CNS01024	*P. micropora*	-	Not found	Nan'ao Island, China	2021.6	117.11	23.48
**CNS00149**	*P. multiseries*	-	Yes	Bohai Sea, China	2019.10	119.94	38.01
CNS00159	*P. multiseries*	0.26 fg/cell	Yes	Bohai Sea, China	2019.10	119.94	38.01
CNS00771	*P. multiseries*	-	Yes	Jiaozhou Bay, China	2020.12	120.33	36.15
CNS00107	*P. multistriata*	-	Yes	Jiaozhou Bay, China	2019.10	120.61	36.05
CNS00142	*P. multistriata*	Not detected	Yes	Bohai Sea, China	2019.10	120.18	38.33
CNS00781	*P. multistriata*	Not detected	Yes	Jiaozhou Bay, China	2020.11	120.33	36.15
CNS00965	*P. multistriata*	Not detected	Yes	Nan'ao Island, China	2021.6	117.11	23.48
CNS01237	*P. multistriata*	Not detected	Yes	Qinhuangdao, China	2021.9	119.86	39.90
CNS01424	*P. multistriata*	Not detected	Yes	Jiaozhou Bay, China	2021.10	120.25	36.10
CNS00043	*P. pungens*	-	Not found	East China Sea, China	2019.5	121.63	27.77
**CNS00055**	*P. pungens*	-	Not found	Jiaozhou Bay, China	2019.7	120.33	36.15
CNS00089	*P. pungens*	-	Not found	East China Sea, China	2019.5	122.28	28.63
CNS00110	*P. pungens*	-	Not found	Bohai Sea, China	2019.10	119.93	38.00
CNS00141	*P. pungens*	Not detected	Not found	Bohai Sea, China	2019.10	120.18	38.33
CNS00153	*P. pungens*	-	Not found	Bohai Sea, China	2019.10	119.93	38.00
CNS00154	*P. pungens*	-	Not found	Bohai Sea, China	2019.10	119.93	38.00

CNS00155	*P. pungens*	-	Not found	Bohai Sea, China	2019.10	119.93	38.00
CNS00156	*P. pungens*	-	Not found	Bohai Sea, China	2019.10	119.93	38.00
CNS00973	*P. pungens*	-	Not found	Qinzhou Bay, China	2021.6	108.68	21.64
CNS01028	*P. pungens*	-	Not found	Nan'ao Island, China	2021.6	117.11	23.48
CNS01042	*P. pungens*	-	Not found	Qinzhou Bay, China	2021.6	108.68	21.64
CNS01238	*P. pungens*	-	Not found	Qinhuangdao, China	2021.9	119.86	39.90
CNS00609	*P. sabit*	-	Not found	Jiaozhou Bay, China	2020.10	120.23	36.04
CNS00097	*Pseudo-nitzschia* sp.	-	Not found	West Pacific	2019.6	121.56	21.40
CNS01031	*Pseudo-nitzschia* sp.	Not detected	Not found	South China Sea, China	2021.5	116.44	14.50

Note: *Pseudo-nitzschia* strains used for whole-genome sequencing were marked in bold. *Pseudo-nitzschia* species were considered as toxic species were marked in red color. For DA analysis, "-" stands for not analyzed in this study, " Not detected " stands for DA were not detected through DA analysis. For *dab* gene cluster analysis, "Yes" stands for *dab* gene cluster was identified in the genome of this strain, “Not found” stands for no *dab* gene has been found in their genomes.

Table S9. Species identification of *Pseudo-nitzschia* strains analyzed in this study

Strain	Accession of ITS1-5.8S-ITS2 sequence	Species identification	Accession of closest sequence	Alignment length (bp)	PID
CNS00108	OR978379	*P. americana*	EU523099	768	100.00%
CNS00151	OR978381	*P. americana*	MZ267621	797	99.87%
CNS01029	OR978390	*P. brasiliana*	KX572957.1	781	100.00%
CNS00969	OR978386	*P. galaxiae*	DQ336158	703	99.57%
CNS01037	OR978392	*P. galaxiae*	MT039947	607	99.51%
CNS01103	OR978394	*P. galaxiae*	MT039947	607	99.51%
CNS01024	OR978388	*P. micropora*	MZ267620	716	100%
CNS00149	OR978380	*P. multiseries*	LC636534.1	702	100.00%
CNS00771	OR978383	*P. multiseries*	MZ267625	737	100%
CNS00107	OR978378	*P. multistriata*	MT039977	786	100%
CNS00781	OR978384	*P. multistriata*	KT247441	873	99.43%
CNS00965	OR978385	*P. multistriata*	MK411975	750	100%
CNS01237	OR978395	*P. multistriata*	MK411975	750	100%
CNS01424	OR978397	*P. multistriata*	MT039991	788	100%
CNS00973	OR978387	*P. pungens*	MZ267622	753	100%
CNS01028	OR978389	*P. pungens*	MZ267622	753	100%
CNS01042	OR978393	*P. pungens*	MZ267622	753	100%
CNS01238	OR978396	*P. pungens*	KT247433	781	100%
CNS00609	OR978382	*P. sabit*	MT040008	686	100.00%
CNS01031	OR978391	*Pseudo-nitzschia* sp.	MZ267627	617	97.24%

Table S10. Statistics of sequencing data and draft genomes of different *Pseudo-nitzschia* strains.

Strain ID	Species	Raw base (G)	Clean base (G)	Bacteria proportion (%)	Estimation Genome Size (Mb)	Genome sequencing depth (X)	Scaffold N50 (kb)	Assembly Genome Size (Mb)
CNS00108	*P. americana*	7.55	7.53	20.71			28.79	83.72
CNS00151	*P. americana*	5.66	5.63	0.20	53.37	106	15.18	60.48
CNS00138	*P. americana*	6.36	6.33	7.24	65.28	90	22.26	75.78
CNS01029	*P. brasiliana*	10.28	10.18	0.21	40.48	253	14.81	47.79
CNS00150	*P. cuspidata*	5.30	5.28	0.28	48.37	109	3.64	73.96
CNS00135	*P. delicatissima*	6.44	6.41	17.81			13.93	63.02
CNS01103	*P. galaxiae*	12.31	12.22	0.13	28.64	429	3.64	45.27
CNS01037	*P. galaxiae*	10.84	10.82	0.19	33.94	319	10.43	69.28
CNS00969	*P. galaxiae*	10.46	10.44	6.57	36.72	266	5.09	51.71
CNS00090	*P. hainanensis*	7.27	7.24	0.32	58.67	124	6.64	76.85
CNS01024	*P. micropora*	10.72	10.70	3.51	38.36	270	24.44	44.87
CNS00133	*P. micropora*	5.91	5.90	7.24	36.64	150	14.39	46.01
CNS00159	*P. multiseries*	3.78	3.74	0.04	252.80	15	5.92	287.00
CNS00771	*P. multiseries*	10.21	10.16	9.17	235.46	39	6.24	329.86
CNS00107	*P. multistriata*	7.83	7.82	0.21	70.55	111	8.73	76.86
CNS00781	*P. multistriata*	10.83	10.78	0.08	65.87	164	8.17	74.84
CNS01424	*P. multistriata*	28.07	28.02	0.10	64.45	435	806	100.76
CNS00142	*P. multistriata*	7.17	7.10	0.16	68.37	105	9.80	69.94
CNS01237	*P. multistriata*	11.50	11.41	0.17	63.96	179	10.29	71.33
CNS00965	*P. multistriata*	10.77	10.69	11.35			10.47	79.98
CNS00043	*P. pungens*	6.09	5.82	37.45			7.96	123.17
CNS00089	*P. pungens*	6.37	6.36	19.88			19.57	85.97
CNS00110	*P. pungens*	4.73	4.72	0.22	94.07	50	8.66	100.49
CNS00153	*P. pungens*	5.97	5.86	0.29	75.20	79	7.60	108.94
CNS00154	*P. pungens*	8.36	8.18	0.33	74.16	112	7.41	131.00
CNS00155	*P. pungens*	6.86	6.61	0.32	78.41	87	5.70	156.40
CNS00156	*P. pungens*	6.76	6.63	0.34	80.18	84	6.71	146.10

CNS01042	*P. pungens*	10.20	10.13	0.12	61.34	166	21.32	73.04
CNS00973	*P. pungens*	10.70	10.62	0.17	61.55	174	14.76	74.48
CNS01028	*P. pungens*	10.53	10.46	0.17	62.48	168	11.61	77.39
CNS01238	*P. pungens*	11.11	10.85	0.18	72.56	153	9.52	94.44
CNS00141	*P. pungens*	6.90	6.84	0.21	64.96	106	11.79	75.32
CNS00609	*P. sabit*	5.84	5.83	23.84			119.45	55.89
CNS01031	*Pseudo-nitzschia* sp.	10.32	10.30	1.86	59.82	169	15.47	71.48
CNS00097	*Pseudo-nitzschia* sp.	7.33	7.32	58.78			6.39	64.51

Note: Sequencing data with bacteria proportion >= 20% are not used to calculated estimation genome size and genome sequencing depth. Scaffold N50 statistics are based on contigs of size >= 500 bp. Assembly genome size statistics are based on contigs of size >= 0 bp. Sequencing data with bacteria proportion more than 10% are not used for analyzing the estimation of genome size.

Table S11. Illumina DNA sequencing statistics for chromosome-level genomes of three *Pseudo-nitzschia* species

Species	Insert Size (bp)	Read Length (bp)	Raw Data (Gb)	Clean Data (Gb)
P. delicatissima	350	150	27.85	26.97
P. pungens	350	150	44.24	38.8
P. multiseries	350	150	27.98	27.73
